# IDR-induced CAR condensation improves the cytotoxicity of CAR-Ts against low-antigen cancers

**DOI:** 10.1038/s41589-025-02031-x

**Published:** 2025-09-29

**Authors:** Xinyan Zhang, Qian Xiao, Longhui Zeng, Fawzaan Hashmi, Kazuki Sato, Tobias Max Philipp Hartwich, Miranda Mansolf, Yang Yang-Hartwich, Xiaolei Su

**Affiliations:** 1 Department of Cell Biology, Yale School of Medicine, New Haven, CT, USA.; 2 Yale College, New Haven, CT, USA.; 3 Department of Obstetrics, Gynecology, and Reproductive Sciences, Yale School of Medicine, New Haven, CT, USA.; 4 Yale Cancer Center, New Haven, CT, USA.; 5 Yale Center for Immuno-Oncology, New Haven, CT, USA.; 6 Yale Center for systems and Engineering Immunology, New Haven, CT, USA.; 7 Yale Stem Cell Center, New Haven, CT, USA.; 8 Present address: Institute of Modern Biology, Nanjing University, Nanjing, China.

## Abstract

Chimeric antigen receptor (CAR)-T cell therapies have shown remarkable efficacies for treating otherwise intractable cancers. However, current clinically approved CAR-T therapies are limited by low antigen sensitivity, impeding their efficacy against cancers with low antigen expression. Here, to address this issue, we engineered CARs targeting CD19, CD22 and HER2 by including intrinsically disordered regions (IDRs) that promote signaling condensation. We discovered that the CAR fused with an IDR from FUS, EWS or TAF15 promoted the formation of CAR-T conjugation with cancer targets, the mechanical strength of CAR-T synapses and membrane-proximal signaling, which led to an increased release of cytotoxic factors and a higher killing activity toward low-antigen-expressing cancer cells in vitro. Moreover, the FUS IDR CAR-T induced improved antitumor effects in both blood cancer and solid tumor models. No spontaneous activation in the absence of antigen was observed. Together, our work demonstrates IDRs as a new toolset for improving CAR-T function through inducing biomolecular condensation.

T cells engineered with the chimeric antigen receptor (CAR) emerge as a versatile and effective toolset for cancer immunotherapy. CAR-Ts have been implemented in combating a variety of cancers and have achieved unprecedented success in treating blood malignancy^1–4^. These achievements encouraged further development of CAR-Ts targeting an expanding pool of cancerous, infectious, autoimmune and fibrotic diseases^5–10^.

Meanwhile, CAR-T therapy is still constrained by a few major obstacles, one of which is limited signaling efficiency. Although derived from the T cell receptor (TCR), CAR displays a much lower antigen sensitivity than TCR: a few hundred or more antigen molecules are required to activate a CAR-T cell^11–13^, whereas a single peptide-loaded major histocompatibility complex (MHC) molecule is sufficient to trigger the activation of a normal T cell^14,15^. This low antigen sensitivity not only narrows the targets of CAR-Ts to high-antigen-expressing cancers but also brings a challenge in maintaining sustained CAR-T activity against tumors; CAR-T-treated patients experience an up to 60% relapse, which is mostly caused by antigen loss^16–18^. Therefore, there is a critical need to develop CAR-Ts that can respond to low-antigen-expressing cancer cells.

Mechanistically, the cause for low antigen sensitivity of CAR remains incompletely understood. The binding of antigen to CAR is much tighter (with a *K*_d_ of nM to pM) than the binding of pMHC to TCR (with a *K*_d_ of μM); therefore, the inability of CAR-Ts to be activated by low-antigen-expressing cells more likely results from the signal processing mechanism downstream CAR rather than the binding of CAR to antigen. Previous studies suggested that CARs insufficiently induce membrane-proximal signaling, including ZAP70 activation and LAT (linker for activation of T cells) phosphorylation, and LAT is weakly engaged in the CAR pathway^13,19,20^. LAT enhances TCR signal transduction by promoting the macromolecular complex assembly and condensation of TCR signaling molecules^21–24^. These suggested an opportunity to engage condensation to improve CAR-T signaling, especially on its response to low-antigen-expressing cancers.

Intrinsically disordered regions (IDRs) have received increasing attention because of their ability to form biomolecular condensates. IDRs do not typically fold into a well-defined three-dimensional structure. Instead, they form condensates through weak intermolecular and intramolecular interactions^25,26^. These condensates display unique biochemical activities by enriching and organizing effector molecules to promote cell signaling^27,28^. Here we decided to induce CAR condensation by constructing CAR–IDR fusion proteins. We identified a few IDRs, including those from FUS, EWS and TAF15, that promote the membrane-proximal signaling, cytotoxic factor production and killing of CAR-Ts against multiple cancer cells expressing low CD19 or HER2. Moreover, no spontaneous activation in the absence of antigen was observed in IDR CAR-Ts, suggesting a difference in signaling outcomes between IDR-induced condensation and previously reported CAR aggregation^29,30^. Together, these results demonstrate that IDRs, although not originally linked to T cell signaling, can serve as a new modular motif to improve the antitumor effect of CAR-Ts. Our work expands the toolkit for CAR engineering, which is traditionally derived from proteins involved in T cell activation.

## Results

### IDRs promote CAR condensation

**Extended Data Fig. 1 |:**
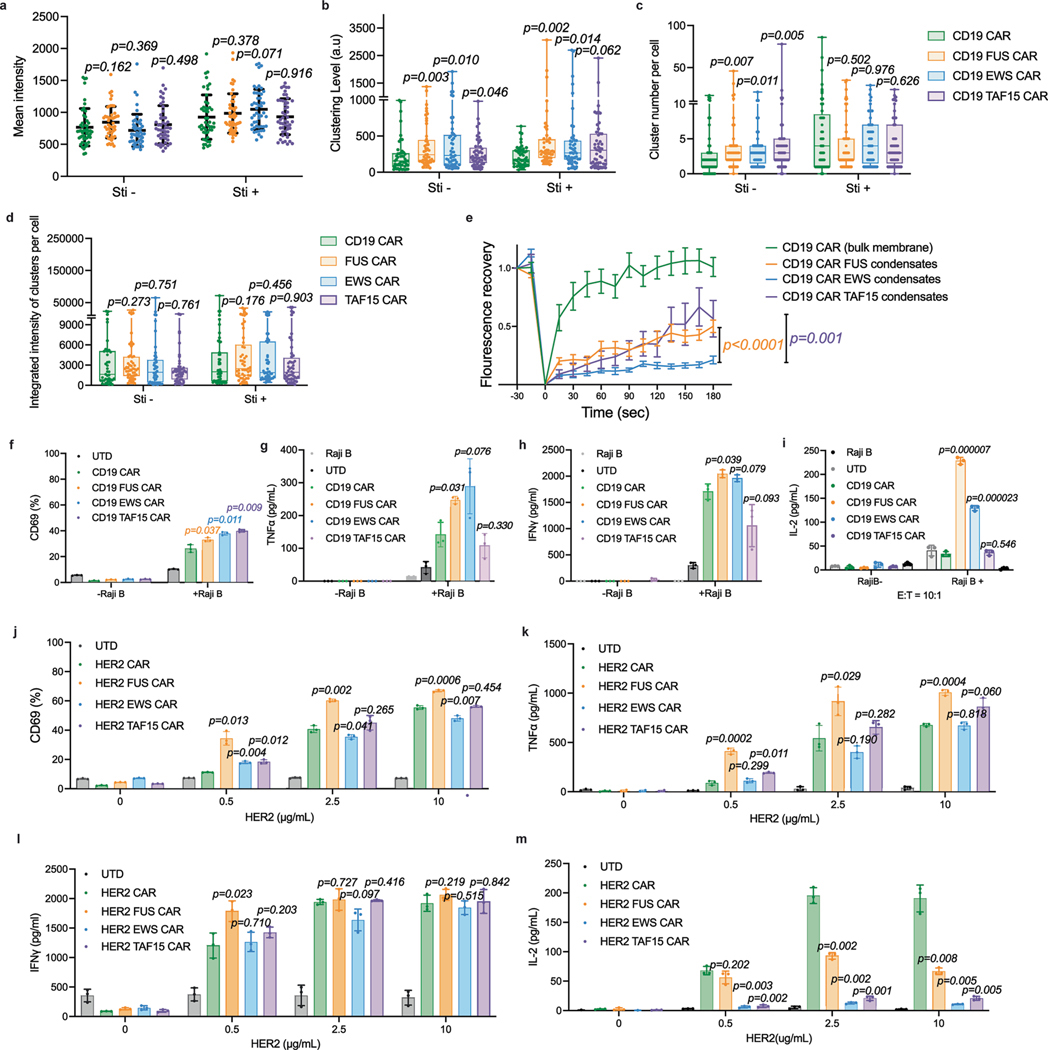
IDR did not trigger CAR-T activation in the absence of antigen. **a**, CAR mean intensity in each cell that was quantified for the following clustering feature. n = 49 cells generated from two donors different than the one in Fig. 1. Shown are mean ± std. All comparisons in this figure were made between individual IDR CARs and the control CAR (*p* value by unpaired two-sided Student’s t test). **b-d**, CAR clustering features. **b**, Clustering level by normalized variance. **c**, CAR cluster number in each cell. **d**, CAR fluorescence per cluster, averaged in each cell. N = 49 cells generated from two donors different than the one in Fig. 1. Shown are box plot including box (25^th^ to 75^th^ percentile). Line inside box is median. Whiskers are min to max within 1.5 IQR. Dots outside whiskers are outliers (values outside 1.5IQR). All comparisons were made between individual IDR CARs and the control CAR (*p* value by unpaired two-sided Mann-Whitney U test). **e**, Fluorescence recovery after photobleaching of CAR-GFP overexpressed in HEK293T cells. Shown are mean ± SEM. N = 10 cells. Comparisons were made between FUS or TAF15 CAR with EWS CAR (*p* value by two-way ANOVA). **f-i**, Activation of CD19 CAR-T in the presence and absence of antigen by Raji B cells at E:T = 10:1. Expression of CD69 was examined by flow cytometry. TNFα, IFNγ and IL-2 release was measured by ELISA. N = 3 technical replicates. Shown are mean ± std. (*p* value by unpaired two-sided Student’s t test). **j-m**, Activation of HER2 CAR-T in the presence and absence of antigen by plate-coated HER2 protein for 1 day. Expression of CD69 was examined by flow cytometry. TNFα, IFNγ and IL-2 release was measured by ELISA. N = 3 technical replicates. Shown are mean ± std. (*p* value by unpaired two-sided Student’s t test).

**Fig. 1 |:**
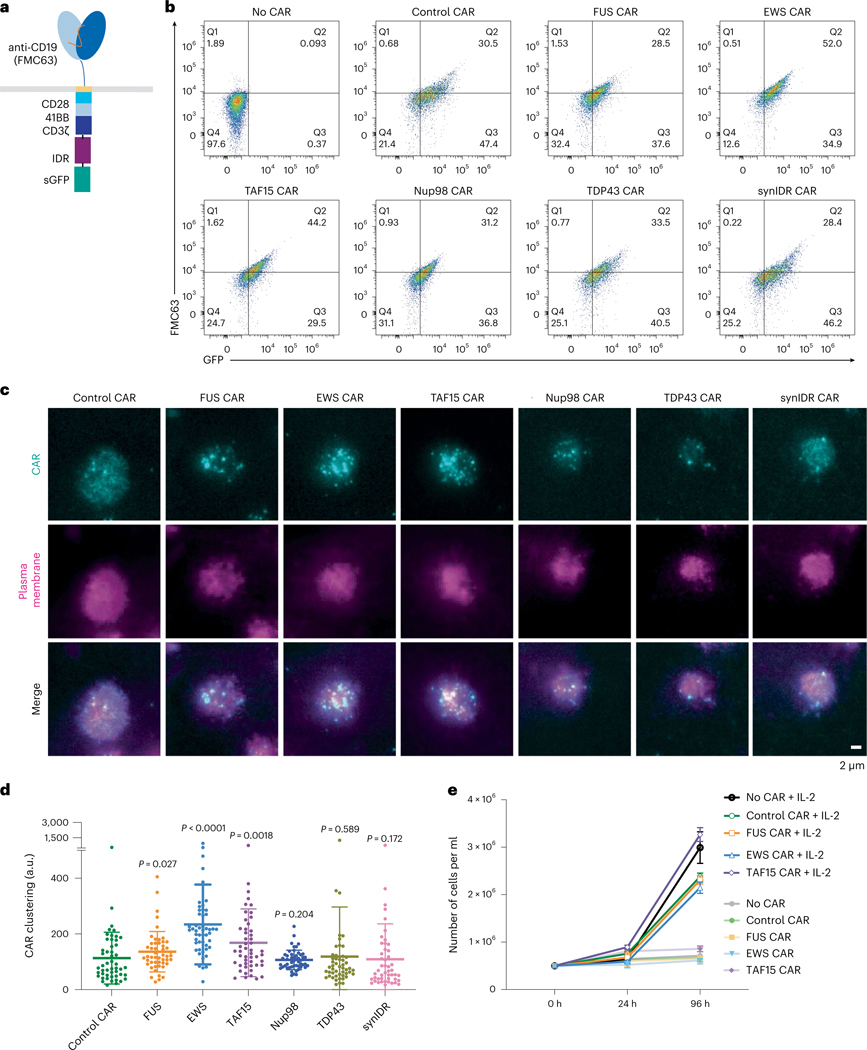
IDRs promote CAR condensation on the T cell surface. **a**, Schematics of the CD19 CAR used in this study. **b**, Expression of control and IDR CARs measured by flow cytometry. The *x* axis displays total CAR expression using a GFP fusion. The *y* axis displays surface expression of CAR by an anti-FMC63 antibody. **c**, Condensation of CAR on the plasma membrane imaged by TIRF microscopy. CAR is tagged by GFP (cyan). The plasma membrane is labeled by a CellMask deep red dye (magenta). **d**, Quantification of CAR clustering by normalized variance (*n* = 50 cells for control, FUS, EWS, TAF15, Nup98 and TDP43 CAR; *n* = 43 for synIDR CAR). Shown are the means ± s.d. All comparisons were made between individual IDR CARs and the control CAR (*P* values determined using a Mann–Whitney *U*-test). **e**, CAR-T cell proliferation with or without IL-2. Quantification of the absolute cell number in the culture with or without IL-2 (*n* = 3 technical replicates). Shown are the means ± s.d.

IDRs contain diverse sequence and structure features. To determine which IDR promotes the condensation of CAR, we selected candidates from six well-characterized IDRs that were previously shown to induce condensation in a cellular environment. These include IDRs from FUS, EWS, TAF15, Nup98, TDP43 and a synthetic IDR (synIDR)^31–37^. We chose CD19 CAR, which is commonly used in research and clinical practice, as an initial model. This CAR is composed of a single-chain variable fragment (scFv, FMC63) that targets CD19, a stalk and transmembrane domain from CD8α and cytosolic signaling domains from 41BB, CD28 and CD3ζ. The IDR was fused to the C terminus of CD3ζ. A superfolder GFP tag, which promotes the folding of fused client proteins^38^, was further attached on the C terminus of IDR for visualization of CAR condensation (Fig. 1a). The superfolder GFP tag enables live-cell imaging, which avoids potential fixation-induced artifacts in characterizing IDR condensation^39^. The DNA fragment encoding the control or IDR CAR was packaged into lentivirus and introduced into primary T cells purified from the human peripheral blood mononuclear cells (PBMCs). Flow cytometry revealed the total cellular expression (by GFP) versus cell surface localization (by FMC63) of individual CARs (Fig. 1b), demonstrating that fusion with IDR did not affect the trafficking of CAR to the cell surface. To visualize the condensation of CAR on the cell surface, we stained live CAR-T cells with a plasma membrane dye CellMask deep red and performed total internal reflection fluorescence (TIRF) microscopy, which effectively reduces the cytosolic background of fluorescence. We found that the CAR fused with FUS, EWS or TAF15 displayed enhanced condensation as compared to the control CAR (Fig. 1c). This is demonstrated by the overall clustering level as quantified by normalized variance under both resting (Fig. 1d) and stimulation conditions (Extended Data Fig. 1b). We scored cells with similar CAR surface expressions to minimize the influence from CAR expression on condensation (Extended Data Fig. 1a). The cluster number per cell was higher in these three IDR CARs as compared to the control CAR in resting but not stimulated cells (Extended Data Fig. 1c), suggesting that clusters might undergo fusion during CAR stimulation. The average fluorescence intensity of cluster per cell was higher in stimulated cells when comparing FUS and EWS CARs to control CAR (Extended Data Fig. 1d). However, because of large variance in the sample, this was not statistically significant. We also compared the mobility of CAR in IDR-promoted condensates by fluorescence recovery after photobleaching. This was performed in HEK293T cells because of their robust CAR expression that is more suitable for high-frequency time-lapse imaging. We found that EWS condensates showed slower recovery than FUS or TAF15 condensates (Extended Data Fig. 1e), suggesting that EWS condensates were more stable. Together, these data demonstrate that FUS, EWS and TAF15 promoted CAR condensation. Therefore, we focused on these three CARs in the subsequent functional assays.

Previous work showed that aggregation of CARs targeting GD2 or CSPG4 induces spontaneous activation in the absence of antigen^29,30^. Therefore, we assessed whether IDR-induced CAR condensation causes a similar spontaneous activation. We found that IDR CAR-Ts did not proliferate in the absence of interleukin 2 (IL-2) (Fig. 1e). The expression of CD69, a T cell activation marker, was similar between the control and IDR CAR-Ts in the absence of antigen (Extended Data Fig. 1f). Moreover, IDR CAR-Ts did not secret detectable tumor necrosis factor alpha (TNFα), interferon-γ (IFNγ) and IL-2 in the absence of antigen (Extended Data Fig. 1g–i). Together, assessed from cell proliferation, activation marker and cytokine production, IDR-induced CAR condensation did not trigger spontaneous signaling.

### IDR from FUS enhances the cytotoxicity of CD19 CAR-T

**Extended Data Fig. 2 |:**
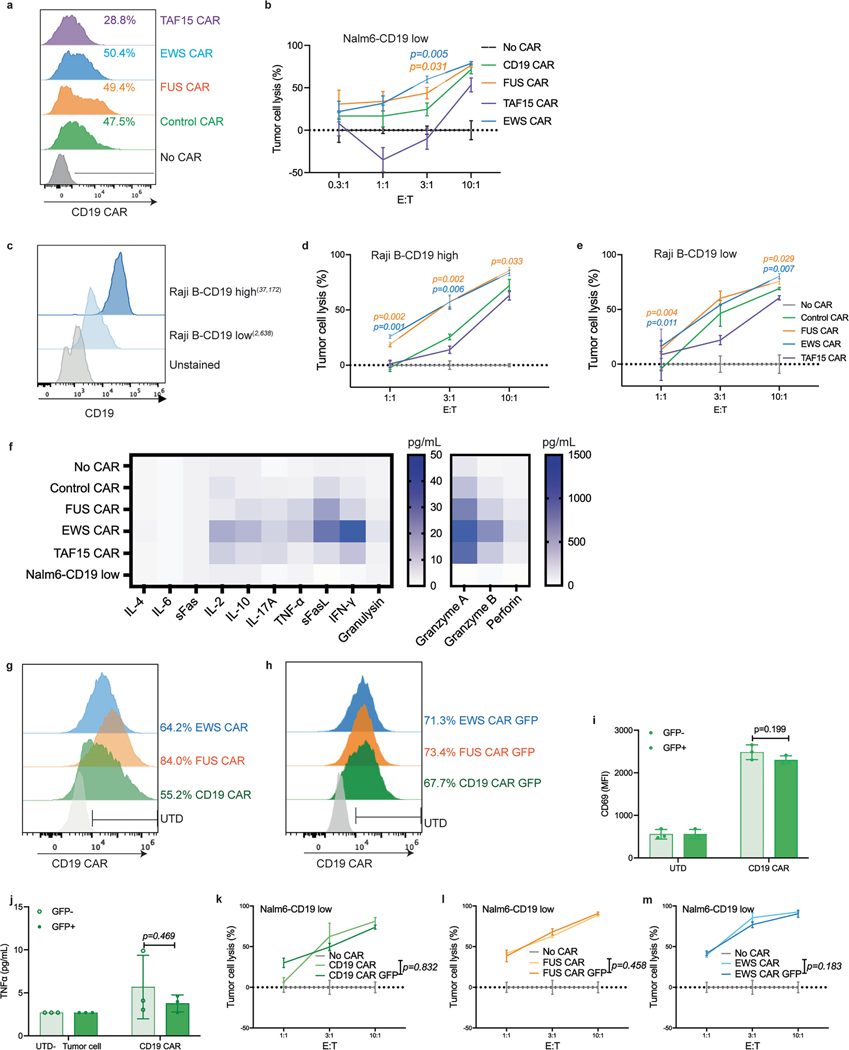
Characterization of IDR CAR-Ts targeting CD19. **a**, Expression of the control or IDR CAR targeting CD19 (scFv FMC63) in human primary T cells from a donor different than that in Fig. 2a measured by flow cytometry. **b**, Cytotoxicity of CD19 CAR-T in vitro (a different donor as that in Fig. 2d) targeting CD19-low Nalm6 cells. N = 3 technical replicates. Shown are mean ± std. (*p* value by unpaired two-sided Student’s t test). **c**, Expression of CD19 in Raji B cells expressing high (37,172 molecules per cell) or low (2,638 molecules per cell) level of CD19 by flow cytometry. **d-e**, Cytotoxicity of CD19 CAR-T against Raji B cells. N = 3 technical replicates. Shown are mean ± std. (*p* value at various E:T ratio *by* unpaired two-sided Student’s t test). **f**, Representative heat map illustrating cytotoxic factors released by CD19 CAR-Ts at an E:T of 3:1 with one-day co-culture. N = 3 technical replicates. Shown are mean in the heatmap. **g-h**, Expression of CD19 control and IDR CARs with and without GFP measured by flow cytometry. **i**, Comparison of CD69 expression induced by control CAR with and without GFP. N = 3 technical replicates. Shown are mean ± std. (*p* value by unpaired two-sided Student’s t test). **j**, Production of TNFα induced by CD19 CAR-T with and without GFP measured by flow cytometry-based ELISA. N = 3 technical replicates. Shown are mean ± std. (*p* value by unpaired two-sided Student’s t test). **k-m**, Comparison of cytotoxicity induced by CAR with and without GFP against Nalm6 cells. N = 3 technical replicates. Shown are mean ± std (*p* value by two-way ANOVA).

**Fig. 2 |:**
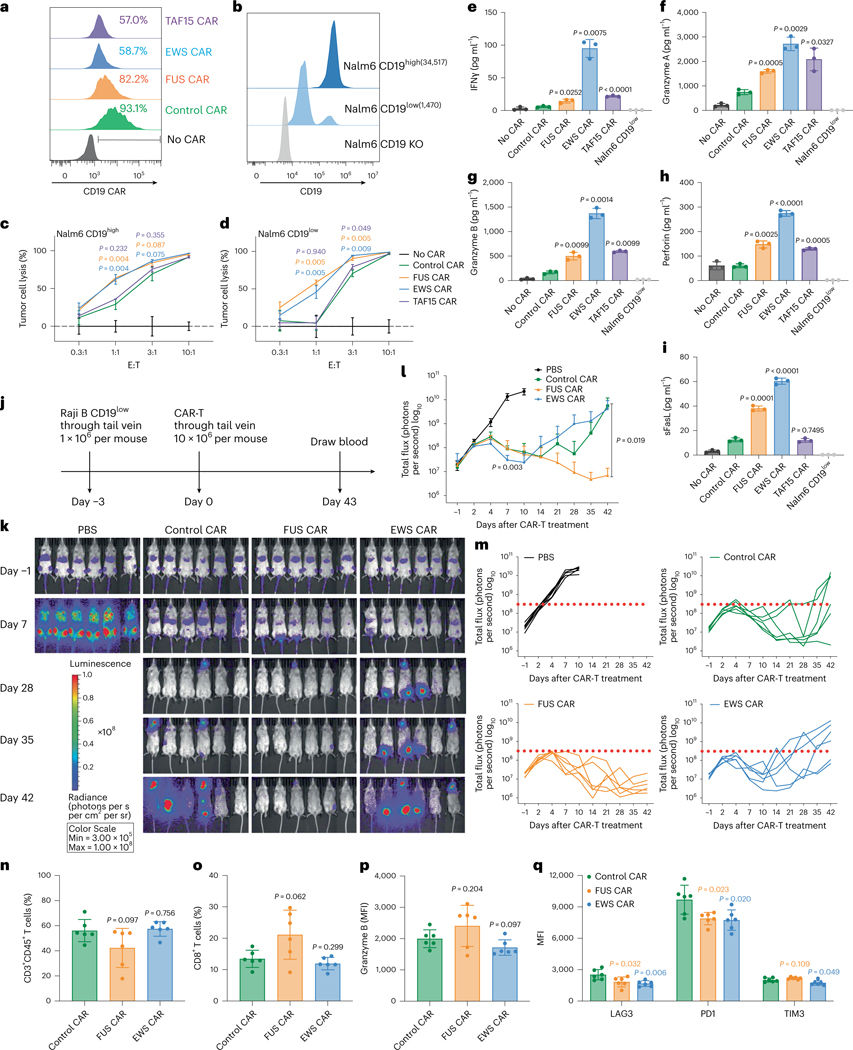
IDRs enhance the cytotoxicity of CD19 CAR-T in vitro and in vivo. **a**, Expression of the control or IDR CAR targeting CD19 (scFv FMC63) in human primary T cells measured by flow cytometry using an anti-FMC63 antibody. **b**, Expression of high (34,517 molecules per cell) or low (1,470 molecules per cell) CD19 in Nalm6 cells. The average number of CD19 antigens on each cell was determined by flow cytometry. **c**,**d**, Cytotoxicity of CD19 CAR-T targeting CD19^high^ or CD19^low^ Nalm6 cells in vitro (*n* = 3 technical replicates). Shown are the means ± s.d. (*P* values at various E:T ratios determined using an unpaired two-sided Student’s *t*-test). **e**–**i**, Production of cytotoxic factors by CD19 CAR-T cocultured with CD19^low^ Nalm6 cells for 1 day at an E:T of 3:1 measured by flow cytometry (*n* = 3 technical replicates). Shown are the means ± s.d. (*P* values determined using an unpaired two-sided Student’s *t*-test). **j**, Timeline of examination of the antitumor effect of CD19 CAR-Ts in a CD19^low^ Raji B-derived xenograft model. **k**–**m**, Tumor progression (rainbow color) quantified by bioluminescent imaging. Both the averaged and individual traces are shown (*n* = 6 mice). Shown are the means ± s.d. A two-way ANOVA was used to compare IDR CAR and control CAR groups over time. An unpaired two-sided Student’s *t*-test was used to compare two groups at specific time points. **n**–**q**, Quantification of pan-T cell abundance, CD8^+^ T percentage, granzyme B expression and T cell exhaustion markers including TIM3, LAG3 and PD1 in total T cells in mouse blood on day 43 after CAR-T infusion analyzed by flow cytometry (*n* = 6 mice). MFI, median fluorescence intensity. Shown are the means ± s.d. (*P* values determined using an unpaired two-sided Student’s *t*-test).

To determine how IDRs affect the cytotoxicity of CAR-Ts against cancer cells, we cocultured the control or IDR CAR-Ts (Fig. 2a) with modified Nalm6, a B cell leukemia line that expresses either high or low CD19 (Fig. 2b). The Nalm6 cells express a luciferase reporter that enables the quantification of cytotoxicity by the luciferase assay. The FUS and EWS but not TAF15 CAR displayed a substantially higher cytotoxicity toward both CD19^high^ and CD19^low^ Nalm6 cells (Fig. 2c, d and Extended Data Fig. 2a,b). This result was recapitulated using another B cell line Raji as the target (Extended Data Fig. 2c–e). The higher cytotoxicity of FUS and EWS CAR could be explained by their higher secretion of cytotoxic factors including granzyme A, granzyme B, perforin, FasL and IFNγ, when CAR-Ts were engaged with CD19^low^ Nalm6 cells (Fig. 2e–i and Extended Data Fig. 2f). Because superfolder GFP was included in the control CAR to balance the protein size increase in the IDR CARs (CAR expression is generally reduced with increased protein sizes), we tested whether superfolder GFP affects CAR-T function. We titrated the lentivirus concentrations to achieve a comparable CAR expression with and without GFP (Extended Data Fig. 2g,h). We found that the inclusion of GFP to the control CAR did not affect the expression of CD69 and the release of TNFα (Extended Data Fig. 2i, j). We also confirmed that the inclusion of GFP did not affect the cytotoxicity of the control or IDR CAR-Ts (Extended Data Fig. 2k–m). Together, these data showed that the FUS and EWS IDRs enhanced the cytotoxicity of CAR-Ts against CD19^low^ cells, which was accompanied with a higher secretion of cytotoxic factors.

**Extended Data Fig. 3 |:**
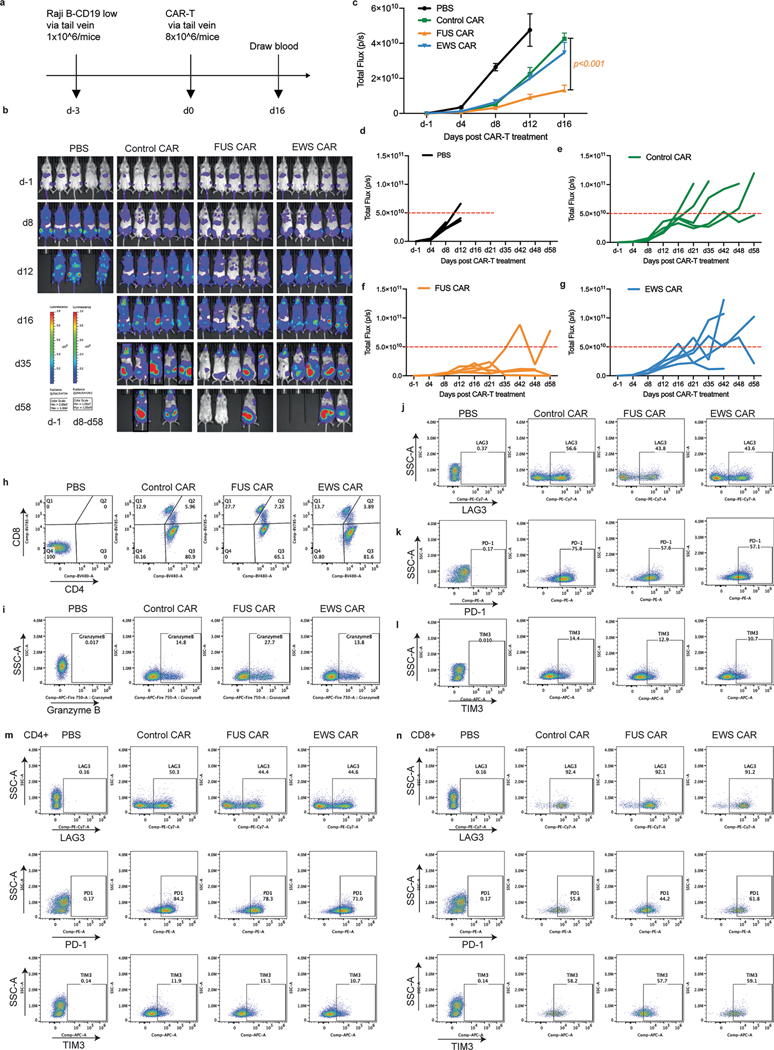
Characterization of IDR CAR-Ts targeting CD19. **a**, Examination of the anti-tumor effect of CD19 IDR CAR-Ts in CD19-low Raji B-derived mouse xenograft model. T cells were derived from a donor different than that in Fig. 2k. **b-g**, Tumor progression (rainbow color) quantified by bioluminescent imaging. Both the individual and averaged traces were shown. N = 5 mice. Shown are mean ± std. (*p* value by two-way ANOVA). **h-l**, Representative flow cytometry plots showing CD4 and CD8 positive T cells, granzyme B expression, and T cell exhaustion markers LAG3, PD1 and TIM3 in total T cells in the blood at day 43 post CAR-T infusion, corresponding to Fig. 2o, p and q. **m-n**. Representative flow cytometry plots showing the expression of exhaustion markers including LAG3, PD1 and TIM3 in CD4 and CD8 T cells at day 43 post CAR-T infusion.

**Extended Data Fig. 4 |:**
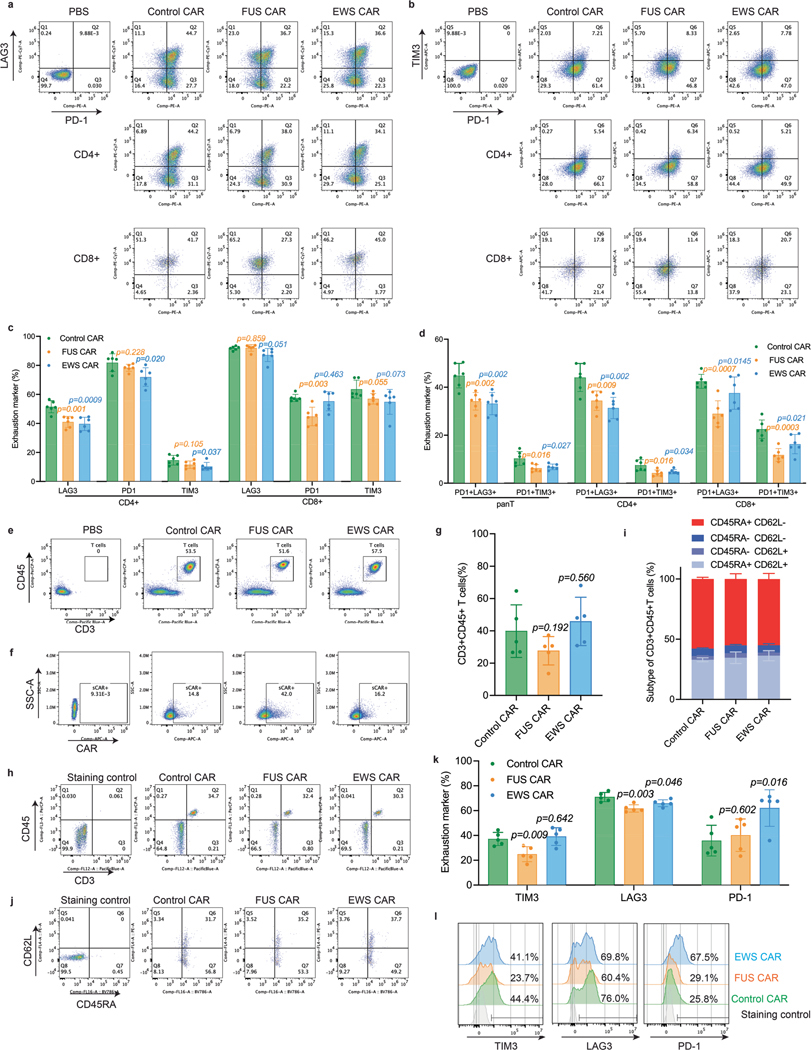
Replicates of the anti-tumor effects of IDR CAR-Ts targeting CD19. **a**. Representative flow cytometry plots showing T cell exhaustion markers defined by PD-1 + LAG3+ double positive in pan, CD4 + , and CD8 + T cell population at day 43 post CAR-T infusion. **b**. Representative flow cytometry plots showing T cell exhaustion markers defined by PD-1 + TIM3+ double positive in pan, CD4 + , and CD8 + T cell population at day 43 post CAR-T infusion. **c**. Quantification of T cell exhaustion markers in CD4+ and CD8 + T cell population corresponding to Extended Data Fig. 3f-g. N = 6 mice. Shown are mean ± std. (*p* value by unpaired two-sided Student’s t test). **d**. Quantification of double positive T cell exhaustion markers PD-1 + LAG3+ and PD-1 + TIM3+ in pan-T, CD4+ and CD8 + T cell population corresponding to Extended Data Fig. 3h-i. N = 6 mice. Shown are mean ± std. (*p* value by unpaired two-sided Student’s t test). **e-f**, Representative flow cytometry plots showing total T cell population, CAR+ population in T cells in the blood at day 43 post CAR-T infusion, corresponding to Fig. 2n. **g**, Quantification of T cell number in the blood on day 16 post CAR-T infusion by flow cytometry in the experiment described in Extended Data Fig. 3a. N = 5 mice. Shown are mean ± std. *(p* value by unpaired two-sided Student’s t test). **h**, Representative flow cytometry plots of Extended Data Fig. 4g. **i**, Quantification of T cell differentiation defined by CD45RA and CD62L in blood at day 16 post CAR-T infusion in the experiment described in Extended Data Fig. 3a. N = 5 mice. Shown are mean ± std. **j**, Representative flow cytometry plots of Extended Data Fig. 4i. **k**, Expression of T cell exhaustion markers during cancer progression on day 16 post CAR-T infusion by flow cytometry in the experiment described in Extended Data Fig. 3a. N = 5 mice. Shown are mean ± std. (*p* value by unpaired two-sided Student’s t test). **l**, Representative flow cytometry plots of Extended Data Fig. 4k.

**Extended Data Fig. 5 |:**
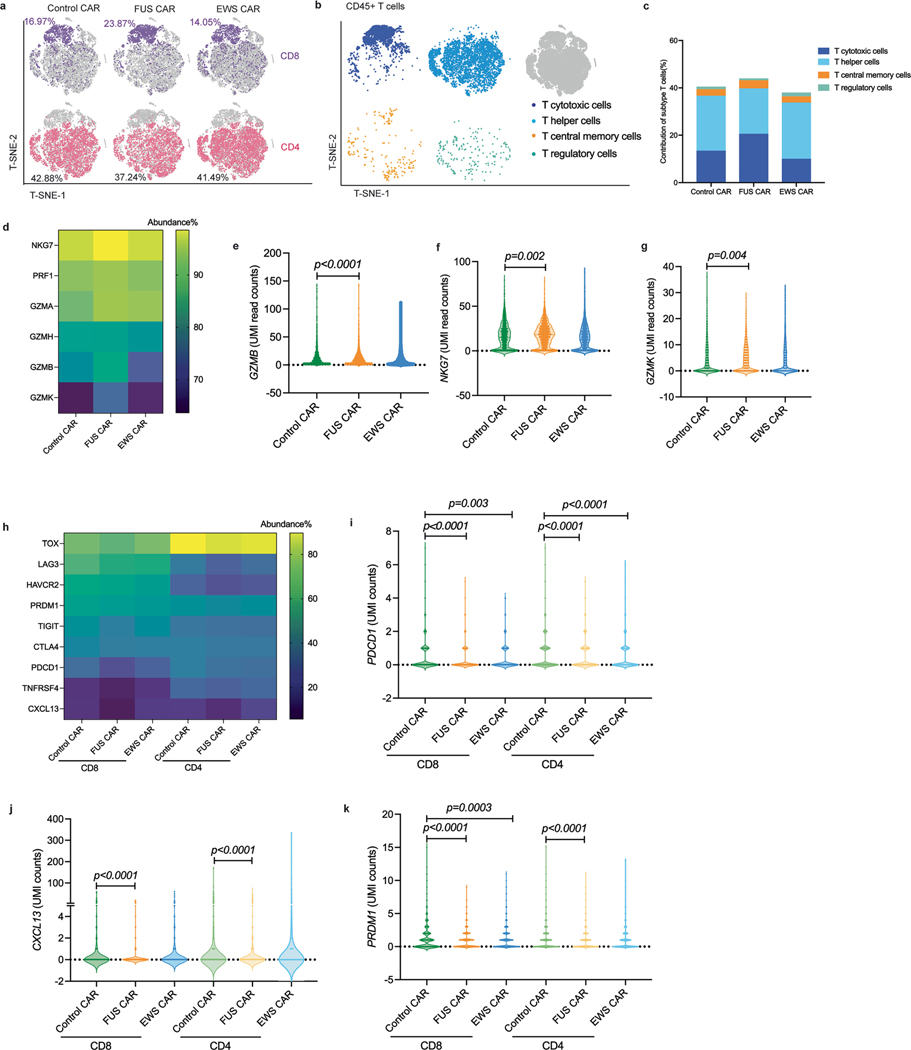
Transcriptomic profiling of T cells in the CD19 cancer model. **a**, T-SNE embedding of CD8^+^T and CD4^+^T cells. T cells were freshly isolated from mouse blood 43 days post CAR-T infusion and subjected to library preparation. See methods for details. **b**, T-SNE embedding of major subtype of T cells. Markers used to define different subtype of T cells are as following: T cytotoxic cells (CD45, CD3, CD8A, GZMB), T helper cells (CD45, CD3, CD4, NFKB1), T central memory cells (CD45, CD3, CCR7, SELL), and T regulatory cells (CD45, CD3, CD4, IL2RA, CTLA4). **c**, Quantification of the abundance of T cell subtypes. Shown are percentage in CD3 and CD45 double positive population. **d**, Heatmap of the abundance of major cytotoxic mediators in CD8 T cells. **e-g**, Read counts of cytotoxic mediators including *GZMB* in total T cell, *NKG7*, and *GZNK* in CD8 T cells. Shown are violin plot including all points (*p* value by two-way ANOVA). **h**, Heatmap of the abundance of major exhaustion markers in the CD8 and CD4 T cells. **i-k**, Read counts of exhaustion-related genes including *PDCD1*, *CXCL13, and PRDM1* in CD8 and CD4 T cells. Shown are violin plot including all points (*p* value by two-way ANOVA).

To assess the tumor-killing effect of IDR CAR-Ts toward CD19^low^ cancer cells in vivo, CD19^low^ Raji B cells expressing a luciferase reporter were injected into the immune-deficient NSG (NOD.Cg-*Prkdc*^*scid*^ *Il2rg*^*tm1Wjl*^/SzJ) mice intravenously. Three days later, the control, FUS or EWS CAR-T cells were infused through the tail vein. The cancer progression was monitored by bioluminescence imaging (Fig. 2j). We found that FUS CAR-T inhibited cancer proliferation better than the control CAR-T (Fig. 2k–m). This effect was repeated using T cells generated from a different donor (Extended Data Fig. 3a–g). EWS CAR-T, though displaying enhanced cytotoxicity in vitro (Fig. 2d and Extended Data Fig. 2b,e) and better tumor control at early time points in vivo (day 7; Fig. 2l), did not achieve a sustained antitumor effect (Fig. 2l). We will discuss reasons later. The enhanced antitumor effect of FUS CAR was accompanied by a mildly (not statistically significant) enhanced percentage of CD8^+^ T cells (Fig. 2o and Extended Data Fig. 3h) and granzyme B expression (Fig. 2p and Extended Data Fig. 3i) but reduced expression of exhaustion markers including LAG3 (mainly contributed by CD4^+^ T cells) and PD1 (mainly contributed by CD8^+^ T cells) (Fig. 2q and Extended Data Figs. 3j–n and 4a–d,k–l). No significant increase was detected in the T cell number (Fig. 2n and Extended Data Fig. 4e–h) or memory phenotype (Extended Data Fig. 4i, j). To further investigate the mechanism of improved antitumor efficacy in FUS CAR-T, we performed single-cell RNA sequencing (scRNA-seq) experiments on T cells isolated from the mouse blood. We found that cytotoxic T cells were more highly represented in the FUS group, as compared to the control or EWS group (Extended Data Fig. 5a–c). The abundance of cells expressing cytotoxic factors (GZMB, GZMK and NKG7) was also higher in the FUS group (Extended Data Fig. 5d–g). Moreover, the expressions of exhaustion markers including PD1 (PDCD1 as the gene name), CXCL13 and PRDM1 were lower in the FUS group (Extended Data Fig. 5h–k). Together, these data suggest that FUS promoted cytotoxicity but reduced exhaustion of CD19 CAR-T, which explains its enhanced antitumor activity in the blood cancer model.

### IDRs from FUS and TAF15 enhance cytotoxicity of HER2 CAR-T

**Extended Data Fig. 6 |:**
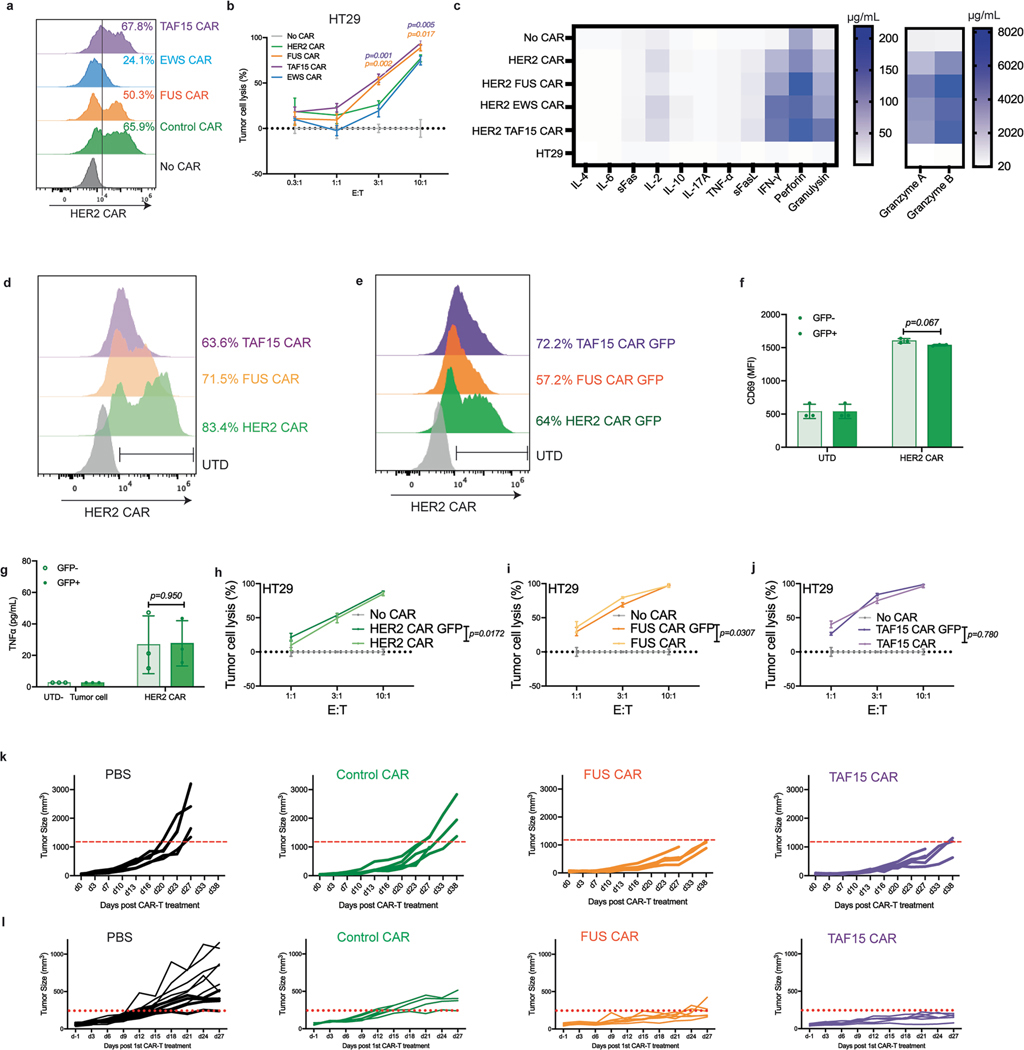
Characterization of IDR CAR-Ts targeting HER2. **a**, Expression of the control or IDR CAR targeting HER2 in human primary T cells from a donor different than that in Fig. 3b measured by flow. **b**, Cytotoxicity of HER2 CAR-T in vitro (a different donor as that in Fig. 3h) targeting HT29 tumor cells. N = 3 technical replicates. Shown are mean ± std. (*p* value by unpaired two-sided Student’s t test). **c**, Heat map of cytotoxic factors released by HER2 CAR-Ts co-cultured with HT29 cells for 1 day at an E:T of 3:1 and quantified by flow cytometry. N = 3 technical replicates. Shown are mean. **d-e**, Expression of HER2 control and IDR CARs with and without GFP. CAR surface expression was stained with AF647 anti-myc antibody and measured by flow cytometry. **f**, Comparison of CD69 expression for control CAR with and without GFP co-cultured with HT29 for 24 hours at the E:T ratio of 3:1. N = 3 technical replicates. Shown are mean ± std. (*p value* by unpaired two-sided Student’s t test). **g**, Production of TNFα induced by HER2 CAR-T with and without GFP co-cultured with HT29 cells for 1 day at an E:T of 3:1. N = 3 technical replicates. Shown are mean ± std. (*p value* by unpaired two-sided Student’s t test). **h-j**. Comparison of cytotoxicity induced by CAR with and without GFP against HT29 cells. N = 3 technical replicates. Shown are mean ± std. (*p* value two-way ANOVA). **k**, Individual traces of cancer progression, corresponding to Fig. 3o. **l**, Individual traces of cancer progression, corresponding to Fig. 3q.

**Fig. 3 |:**
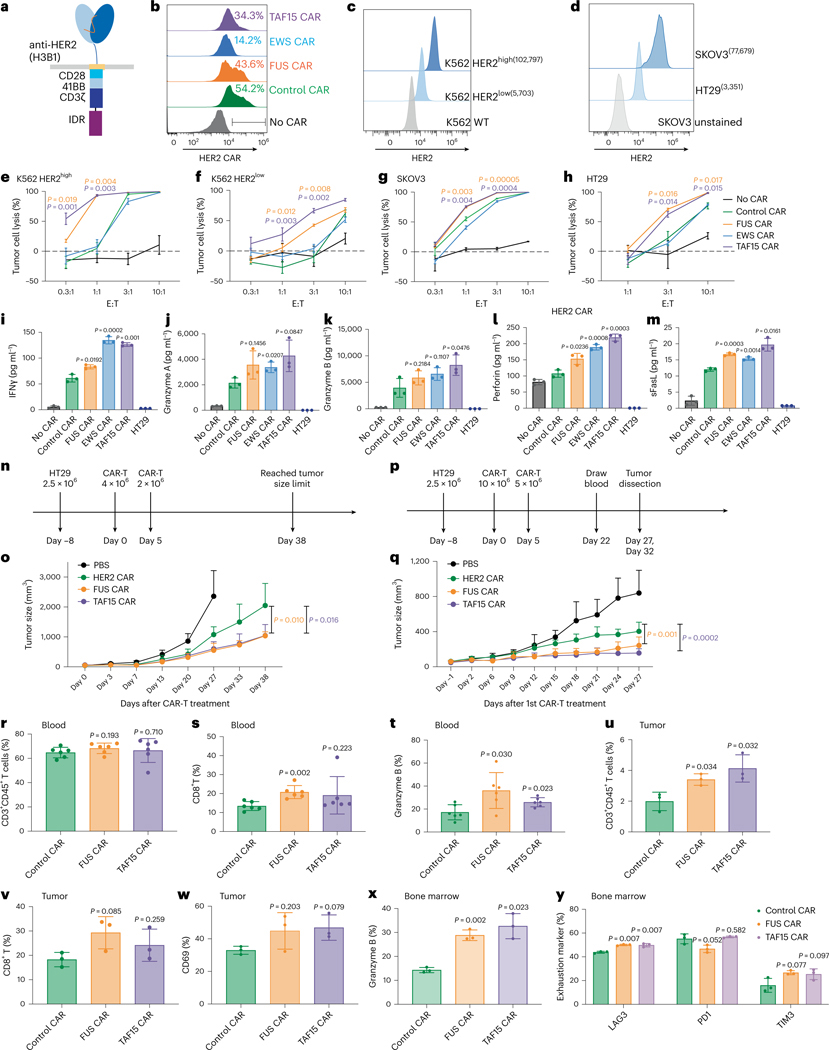
IDRs enhance the cytotoxicity of HER2 CAR-T in vitro and in vivo. **a**, Schematics of the HER2 CAR used in this study. The scFv targeting HER2 is H3B1. **b**, Expression of the control or IDR CAR targeting HER2 in human primary T cells by flow cytometry. WT, wild type. **c**,**d**, Quantification of the HER2 level in K562, SKOV3 and HT29 cells. **e**–**h**, Cytotoxicity of HER2 CAR-T in vitro targeting HER2^high^ or HER2^low^ K562 cells, SKOV3 and HT29 cells (*n* = 3 technical replicates). Shown are the means ± s.d. (*P* values at various E:T ratios determined using an unpaired two-sided Student’s *t*-test). **i**–**m**, Production of cytotoxic factors by CAR-T cocultured with HT29 cells for 1 day at an E:T of 3:1 (*n* = 3 technical replicates). Shown are the means ± s.d. (*P* values determined using an unpaired two-sided Student’s *t*-test). **n**, Timeline of examination of the antitumor effect of HER2 IDR CAR-Ts in subcutaneously engrafted HT29 tumor model. Low doses of CAR-T were infused intravenously. **o**, Quantification of tumor progression in vivo by measuring the tumor size using an electronic digital caliper (*n* = 5 mice). Shown are the means ± s.d. (*P* values determined using a two-way ANOVA). **p**, HT29 tumor was treated with high doses of CAR-T injected intravenously. The CAR-T cells were generated from a donor different than the one in **n**,**o**. **q**, Quantification of tumor progression (*n* = 6 mice). Shown are the means ± s.d. (*P* values determined using a two-way ANOVA). **r**–**t**, Quantification of total T cell and CD8^+^ T cell abundance and granzyme B expression in blood on day 22 after CAR-T infusion by flow cytometry (*n* = 6 mice). Shown are the means ± s.d. (*P* values determined using an unpaired two-sided Student’s *t*-test). **u**–**w**, Quantification of tumor-infiltrated T cells, CD8 percentage and CD69 expression in tumor-infiltrated T cells on day 27 after CAR-T infusion by flow cytometry (*n* = 3 mice). Shown are the means ± s.d. (*P* values determined using an unpaired two-sided Student’s *t*-test). **x**,**y**, Quantification of expression of granzyme B and exhaustion markers including LAG3, PD1 and TIM3 in bone marrow T cells on day 27 after CAR-T infusion (*n* = 3 mice). Shown are the means ± s.d. (*P* values determined using an unpaired two-sided Student’s *t*-test).

To determine whether the effect of IDR in promoting cytotoxicity applied to CARs beyond CD19, we constructed IDR CARs targeting HER2 (Fig. 3a), an antigen commonly overexpressed in multiple solid tumors including breast, ovarian, lung and colorectal cancers. Human primary T cells were infected with lentivirus encoding the control, FUS, TAF15 or EWS CAR (Fig. 3b). Similar to the case of CD19 CAR, IDR did not trigger spontaneous activation (without antigen) of HER2 CAR as assessed by CD69 expression and the release of TNFα, IFNγ and IL-2 (Extended Data Fig. 1j–m). Next, we selected multiple target cell lines for testing cytotoxicity: the lymphoblast K562 cell line ectopically expressing high or low HER2 (Fig. 3c), the ovarian cancer cell line SKOV3 expressing high HER2 and the colon cancer cell line HT29 expressing low HER2 (Fig. 3d). These target cells were cocultured with the control or IDR CAR-Ts. We found that FUS and TAF15 CAR-Ts displayed higher cytotoxicity toward all four cell lines tested, as compared to the control CAR-T (Fig. 3e–h and Extended Data Fig. 6a,b). Consistent with that, FUS and TAF15 CAR-Ts secreted a higher level of cytotoxic factors, including IFNγ, perforin and FasL, than the control CAR-T (Fig. 3i–m and Extended Data Fig. 6c). When comparing HER2 CAR with and without superfolder GFP, we did not find a significant difference in CD69 expression, TNFα production or cytotoxicity (Extended Data Fig. 6d–j), suggesting that the inclusion of superfolder GFP did not significantly affect HER2 CAR-T function. Together, these data suggest that FUS and TAF15 enhanced the cytotoxicity of both HER2^high^ and HER2^low^ CAR-Ts in vitro.

**Extended Data Fig. 7 |:**
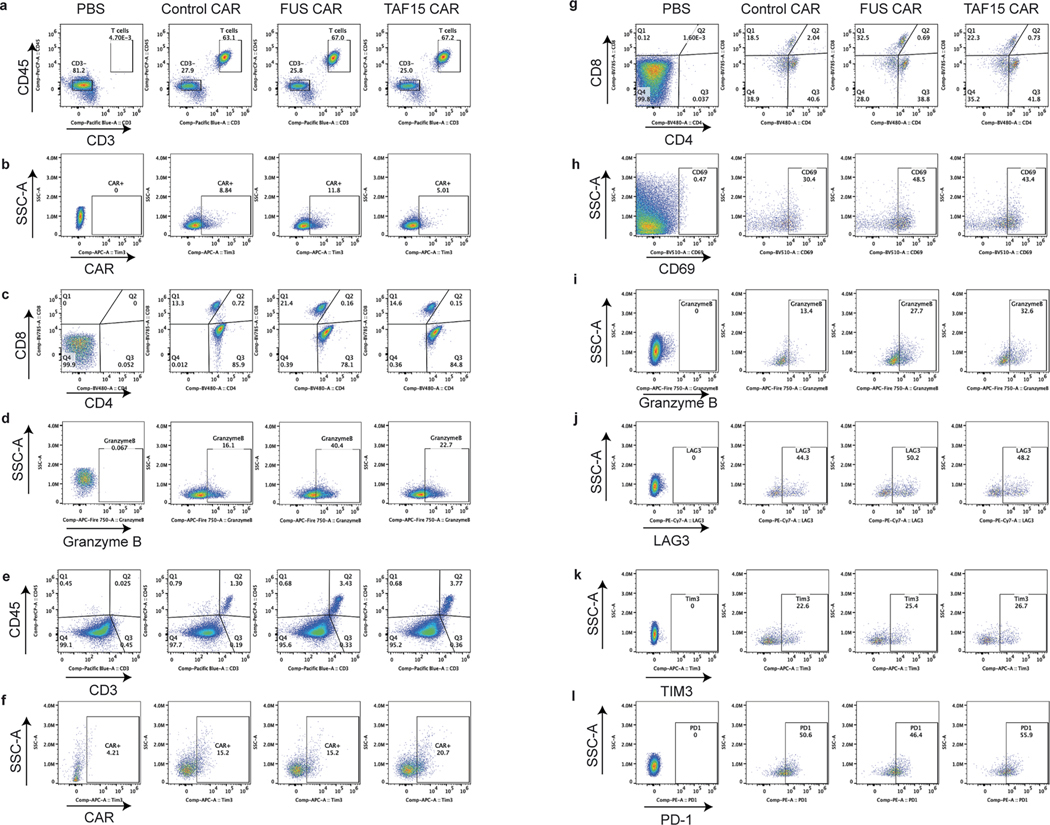
Characterization of IDR CAR-Ts targeting HER2. **a-d**, Representative flow cytometry plots showing blood T cell population, CAR + T cells, CD8 + T cells, and granzyme B expression, corresponding to Fig. 3r-t. **e-h**, Representative flow cytometry plots showing tumor infiltered T cell population, CAR + T cells, CD8 + T and CD69 expression, corresponding to Fig. 3u-w. **i-l**, Representative flow cytometry plots showing bone marrow T cell granzyme B expression, and exhaustion marker expression including LAG3, PD1 and TIM3, corresponding to Fig. 3x-y.

To determine the antitumor effect of IDR CAR-Ts toward HER2^low^ cells in vivo, HT29 cells were injected into the immune-deficient NSG mice subcutaneously. Eight days later, the control or IDR CAR-T cells were infused through the tail vein. A second dose was administered 5 days later. The tumor progression was monitored by an electronic caliper over 6 weeks (Fig. 3n). We found that FUS and TAF15 CAR-T inhibited tumor growth better than the control CAR-T (Fig. 3o and Extended Data Fig. 6k), which is consistent with the in vitro killing results. To characterize T cell phenotypes, we repeated the experiment with two modifications: (1) by increasing the infused CAR-T cell number so that sufficient tumor-infiltrating T cells could be isolated for analysis and (2) by terminating the experiment at an earlier time point when tumors did not undergo necrosis and T cells were not entirely exhausted (Fig. 3p). During the new trial, we observed that FUS and TAF15 slowed tumor progression (Fig. 3q and Extended Data Fig. 6l), which is consistent with the result from previous experiments. When analyzing circulating T cells, we observed a higher percentage of CD8^+^ T cells in the FUS group (Fig. 3r,s and Extended Data Fig. 7a–c) and a higher expression of granzyme B in both the FUS and TAF15 group (Fig. 3t and Extended Data Fig. 7d). By isolating tumor-infiltrating T cells, we found that the FUS and TAF15 group showed an increased number of tumor-infiltrating T cells (Fig. 3u and Extended Data Fig. 7e,f), although the T cell numbers in blood were comparable across the control, FUS and TAF15 groups (Fig. 3r and Extended Data Fig. 7a,b). An increase in CD8^+^ population and CD69 expression was also observed, albeit not statistically significant, which is potentially because of the large variance in samples (Fig. 3v,w and Extended Data Fig. 7g,h). For the bone marrow T cells, we also observed a higher granzyme B expression in the FUS and TAF15 group (Fig. 3x and Extended Data Fig. 7i). No significantly lower expression was found in the exhaustion makers (Fig. 3y and Extended Data Fig. 7j–l). Together, these data suggest that FUS and TAF15 promoted a CD8/cytotoxic signature in vivo, which is consistent with the in vitro data showing that they displayed a higher cytotoxicity.

### IDRs from FUS and EWS enhance cytotoxicity of CD22 CAR-T

**Extended Data Fig. 8 |:**
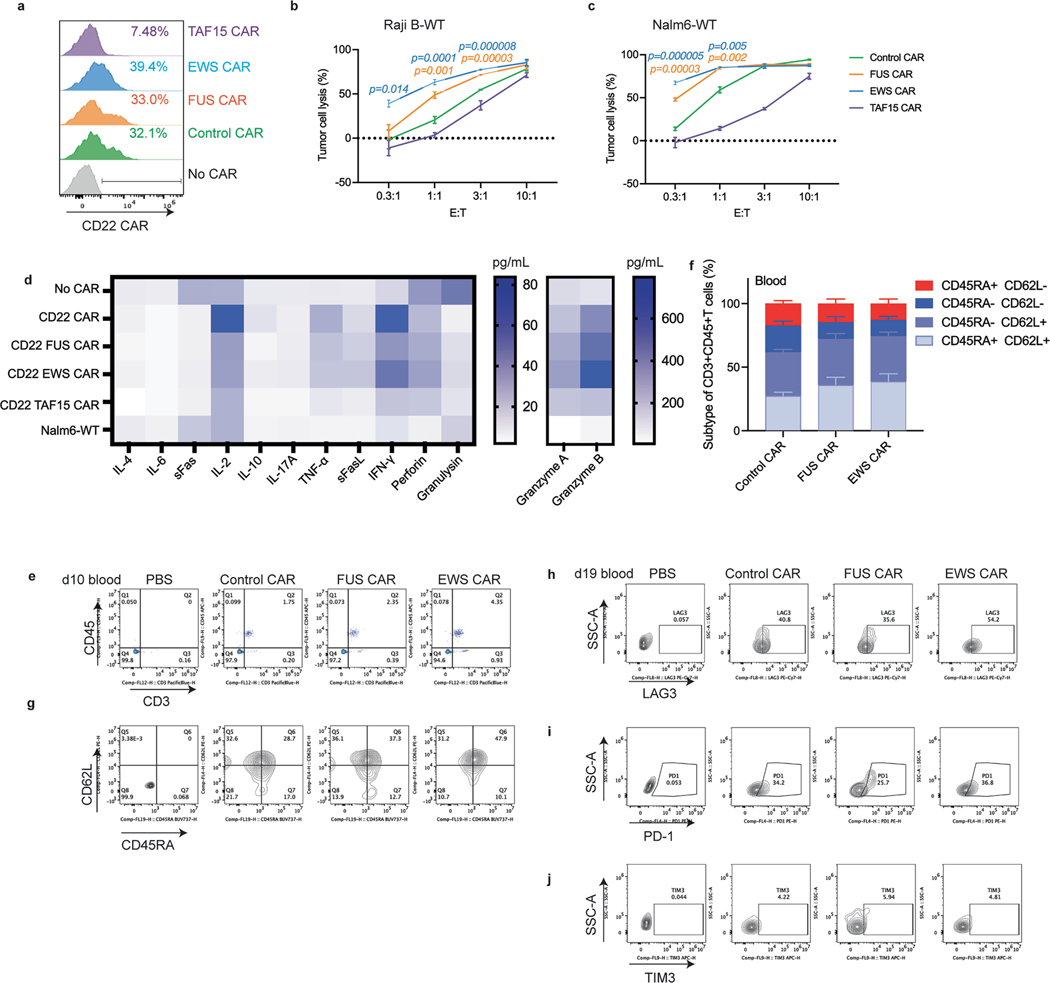
Characterization of IDR CAR-Ts targeting CD22. **a**, Expression of the control or IDR CAR targeting CD22 in human primary T cells from a donor different than that shown in Fig. 4b. **b-c**, Cytotoxicity of CD22 CAR-T generated from a donor different from the one shown in Fig. 4d-e targeting Raji B or Nalm6 cells for 3 or 1 day, respectively, with an effector to target (E:T) ratio from 0.3:1 to 10:1. N = 3 technical replicates. Shown are mean ± std. (*p* value at various E:T ratio by unpaired two-sided Student’s t test). **d**, Representative heat map illustrating cytotoxic factors released by CD22 CAR-Ts co-cultured with the wild-type Nalm6 cells for 1 day at an E:T of 1:1. N = 3 technical replicates. Shown are mean. **e**, Representative flow cytometry plots showing T cell population in the blood at day 10 post CAR-T infusion, corresponding to Fig. 4n. **f**, Quantification of T cell differentiation defined by CD45RA and CD62L in blood at day 10 post CAR-T infusion. N = 5 mice. Shown are mean ± std. **g**, Representative flow cytometry plots and quantification of T cell memory subtypes defined by CD45RA and CD62L at day 10 post CAR-T infusion in Extended Data Fig. 8f. **h-j**, Representative flow cytometry plots showing T cell exhaustion markers including TIM3, LAG3, and PD-1 at day 19 post CAR-T infusion, corresponding Fig. 4o

**Fig. 4 |:**
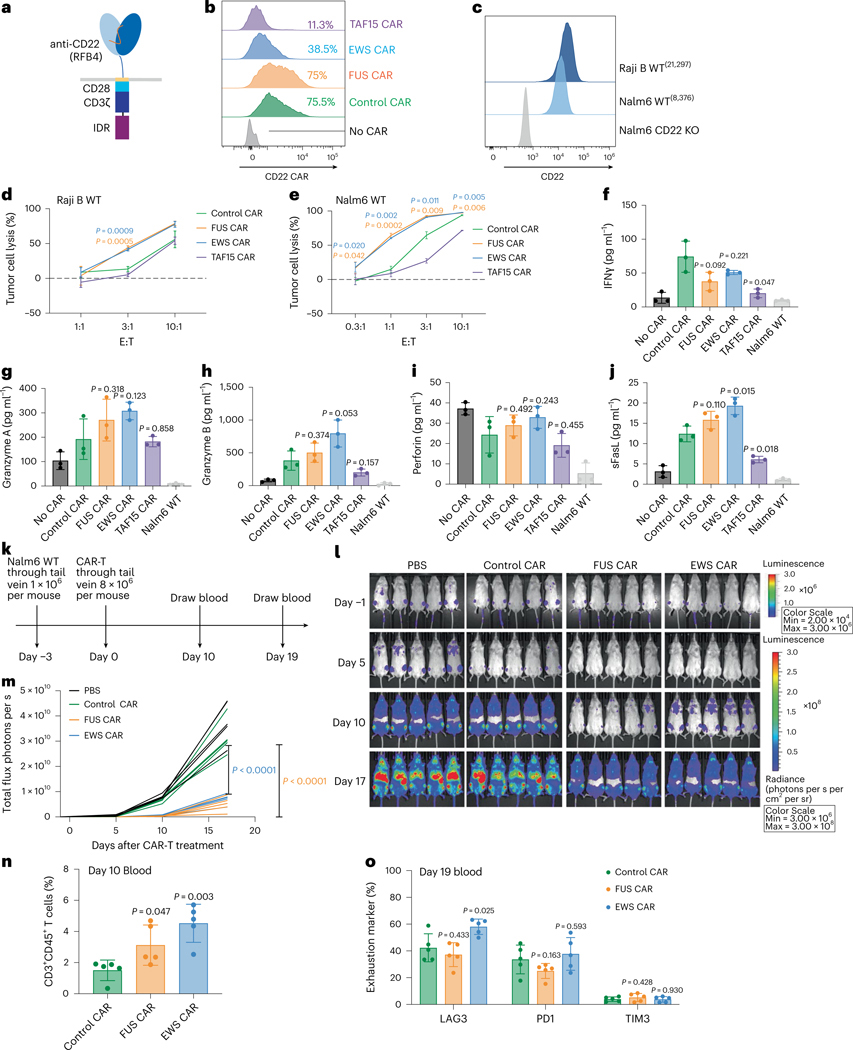
IDRs enhance the cytotoxicity of CD22 CAR-T in vitro and in vivo. **a**, Schematics of the CD22 CAR used in this study. The scFv targeting CD22 is RFB4. **b**, Expression of the control or IDR CAR targeting CD22 in human primary T cells. CAR expression was detected by recombinant CD22 proteins and measured by flow cytometry. **c**, Quantification of the CD22 level in the wild-type Raji B and Nalm6 by flow cytometry. **d**,**e**, Cytotoxicity of CD22 CAR-T in vitro targeting Raji B or Nalm6 cells cocultured for 3 days (**d**) or 1 day (**e**) (*n* = 3 technical replicates). Shown are the means ± s.d. (*P* values at various E:T ratios determined using an unpaired two-sided Student’s *t*-test). **f**–**j**, Production of cytotoxic factors by CAR-T cocultured with the wild-type Nalm6 cells for 1 day at an E:T of 1:1 measured by flow cytometry (*n* = 3 technical replicates). Shown are the mean ± s.d. (*P* values determined using an unpaired two-sided Student’s *t*-test). **k**, Timeline of the antitumor effect of CD22 IDR CAR-Ts in wild-type Nalm6-derived tumor xenograft model. **l**,**m**, Tumor progression (rainbow color) quantified by bioluminescent imaging (*n* = 5 mice). Shown are the means ± s.d. (*P* values determined using a two-way ANOVA). **n**, Quantification of T cells in the blood on day 10 after CAR-T infusion by flow cytometry (*n* = 5 mice). Shown are the means ± s.d. (*P* values determined using an unpaired two-sided Student’s *t*-test). **o**, Expression of T cell exhaustion markers including TIM3, LAG3 and PD1 during cancer progression on day 19 after CAR-T infusion by flow cytometry (*n* = 5 mice). Shown are the means ± s.d. (*P* values determined using an unpaired two-sided Student’s *t*-test).

In addition to the CD19 and HER2 CAR, we also tested how IDRs affect the cytotoxicity of an CD22 CAR (RFB4), which showed very low signaling efficiency because it targets the membrane-distal epitope position on CD22 (refs. 40,41). Using a similar design strategy to the CD19 CAR, we constructed the IDR CARs targeting CD22 by fusing the IDR on the C terminus (Fig. 4a). Human primary T cells were infected with lentivirus encoding the control, FUS, EWS or TAF15 CAR (Fig. 4b). The wild-type Raji B or Nalm6 cells, which express a medium level of CD22 (Fig. 4c), were cocultured with CAR-Ts. We found that FUS and EWS CAR-T displayed a higher cytotoxicity as compared to the control CAR-T when cocultured with either Raji B or Nalm6 cells (Fig. 4d,e and Extended Data Fig. 8a–c). We also measured the release of cytotoxic factors and found that FUS and EWS CAR-Ts released slightly higher granzymes A and B and FasL than the control CAR-T, albeit not statistically significant (Fig. 4f–j and Extended Data Fig. 8d). Together, these data show that FUS and EWS enhanced the cytotoxicity of a low-signaling CD22 CAR-T.

To determine whether IDRs improve the antitumor efficacy of the above low-signaling CD22 CAR in vivo, wild-type Nalm6 cells were injected into NSG mice intravenously. Three days later, the control, FUS or EWS CAR-T cells were infused through the tail vein. The cancer progression was monitored by bioluminescence imaging (Fig. 4k). Similar to the in vitro killing result, FUS and EWS CAR-T inhibited tumor growth better than the control CAR-T (Fig. 4l, m). The circulating T cell number was higher in the FUS and EWS group than the control group (Fig. 4n and Extended Data Fig. 8e). The expression of markers for T cell differentiation (Extended Data Fig. 8f,g) or exhaustion (Fig. 4o and Extended Data Fig. 8h–j) was not significantly different between the control and IDR CAR-Ts. Together, these data suggest that IDRs from FUS and EWS promoted the antitumor effect of a low-signaling CD22 CAR.

### IDRs promote the formation and signaling of CAR-T synapse

**Fig. 5 |:**
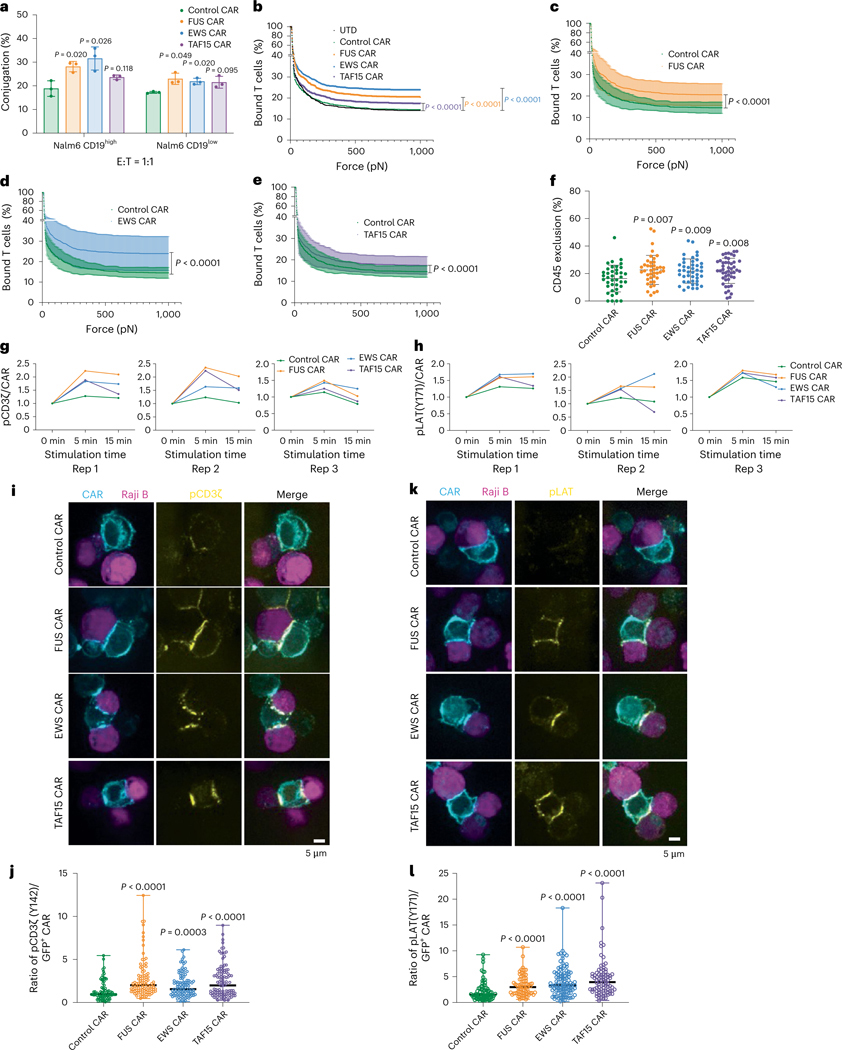
IDRs promote the formation, mechanical strength and signaling of the CAR-T synapse. **a**, Cell–cell conjugation percentage of CD19 control and IDR CAR-Ts with Nalm6 CD19^high^ or CD19^low^ cells (*n* = 3 technical replicates). Shown are the means ± s.d. (*P* values determined using an unpaired two-sided Student’s *t*-test). **b**, Rupture forces to detach CD19 control and IDR CAR-T from Nalm6 CD19^low^ cells by z-Movi from Lumicks. CAR-T cells were generated from *n* = 3 donors. Shown are the means. The mean ranges (mean ± s.d.) are shown in **c**–**e** (*P* values determined using a two-way ANOVA). **f**, CD45 exclusion in the synapse formed between CD19 CAR-T with Raji B CD19^low^ cells was imaged by confocal microscopy. CD19 CAR, *n* = 39 cells; FUS CAR, *n* = 41 cells; EWS CAR, *n* = 43 cells; TAF15 CAR, *n* = 47 cells. CAR-T cells were generated from *n* = 3 donors. Shown are the means ± s.d. (*P* values determined using an unpaired two-sided Student’s *t*-test). **g**,**h**, Phosphorylation kinetics of CD3ζ (pY142) and LAT (pY171). Displayed are traces for three independent experiments using CAR-T cells generated from *n* = 3 donors. **i**,**j**, Phosphorylation of CD3ζ at the CAR-T synapse imaged by confocal microscopy and quantification of pCD3ζ at the CAR-T synapse by ImageJ. The cyan and magenta colors represent CAR-T and tumor cells; the yellow color represents pCD3ζ. CD19 CAR, *n* = 77 conjugates; FUS CAR, *n* = 100 conjugates; EWS and TAF15 CAR, *n* = 107 conjugates (*P* values determined using an unpaired two-sided Mann–Whitney *U*-test). **k**,**l**, Phosphorylation of LAT at the CAR-T synapse imaged by confocal microscopy and quantification of pLAT at the CAR-T synapse by ImageJ. The cyan and magenta colors represent CAR-T and tumor cells; the yellow color represents pLAT. CD19 CAR, *n* = 79 conjugates; FUS CAR, *n* = 81 conjugates; EWS CAR, *n* = 105 conjugates; TAF15 CAR, *n* = 86 conjugates (*P* values determined using an unpaired two-sided Mann–Whitney *U*-test).

Next, we investigated the molecular mechanism by which CAR condensation increases T cell activation. CAR condensation is expected to promote multivalent interactions with antigens, thereby increasing the binding between CAR-T cells and cancer cells. Indeed, all three IDR CAR-Ts formed a higher percentage of cell–cell conjugation with CD19^+^ Nalm6 cells as compared to the control CAR-T (Fig. 5a). To probe the mechanical strength of the cell–cell conjugates, we exploited the z-Movi ‘cell avidity’ instrument by Lumicks^42,43^. Nalm6 cells were preseeded on the chip, followed by CAR-T addition to form the cell–cell conjugates. An increasing acoustic force was applied and the detachment of CAR-Ts from Nalm6 was monitored over time. We found that the force to disassemble the synapse was higher in IDR CAR-T as compared to the control CAR-T (Fig. 5b–e), suggesting that the cell– cell interaction was stronger in the IDR CAR-T group as compared to the control CAR-T group. Next, we investigated the localization of a key signaling protein, CD45, in the synapse. After cell–cell conjugates formed, CD45, a phosphatase that dephosphorylates CAR, was excluded from the synapse because of its large extracellular domain^44^. Our previous work showed that CD45 exclusion is mediated by CAR–antigen interaction and the level of CD45 exclusion influences CAR signaling^40^. Therefore, we examined CD45 exclusion in the synapse and found that CD45 was excluded to a higher level in the IDR CAR group as compared to the control CAR group (Fig. 5f). Together, these data suggest that IDRs promoted the formation, mechanical strength and CD45 exclusion of the immunological synapse formed between CAR-T and cancer cells.

**Extended Data Fig. 9 |:**
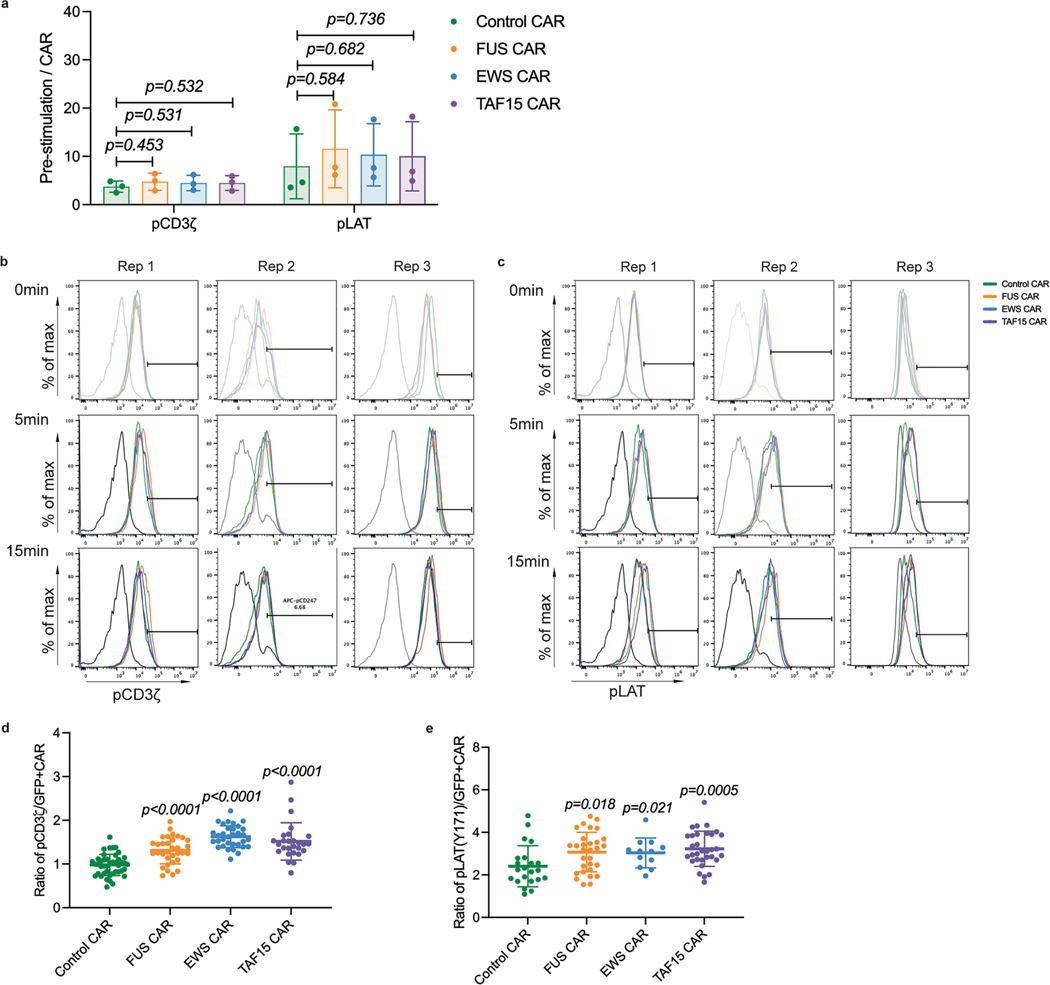
Signaling analysis of the IDR CAR-T. **a**, Phosphorylation of CD3ζ (pY142) and LAT (pY171) at pre-stimulation state. Relative pCD3ζ and pLAT was calculated by dividing the geometric mean of pCD3ζ or pLAT to CARGFP at 0 minute. n = 3 donors. Shown are mean + std (*p value* by unpaired two-sided Student’s t test). **b-c**, Individual flow histograms are shown the expression kinetics of phosphorylated CD3ζ and LAT for CD19 CAR-T with 0, 5 or15 min stimulation of CD19-low Raji B cells. **d-e**, Quantification of pCD3ζ and pLAT at the CAR-T synapse. CAR-T cells were generated from a donor different from the one used in Fig. 5i-l. For pCD3ζ CD19 CAR n = 43 conjugates, FUS and EWS CAR n = 33 conjugates, and TAF15 CAR n = 27 conjugates. For pLAT CD19 CAR n = 22 conjugates, FUS CAR n = 30 conjugates, EWS CAR n = 12 conjugates and TAF15 CAR n= 32conjugates. Shown are mean ± std. (*p value by* unpaired two-sided Mann-Whitney U test).

**Extended Data Fig. 10 |:**
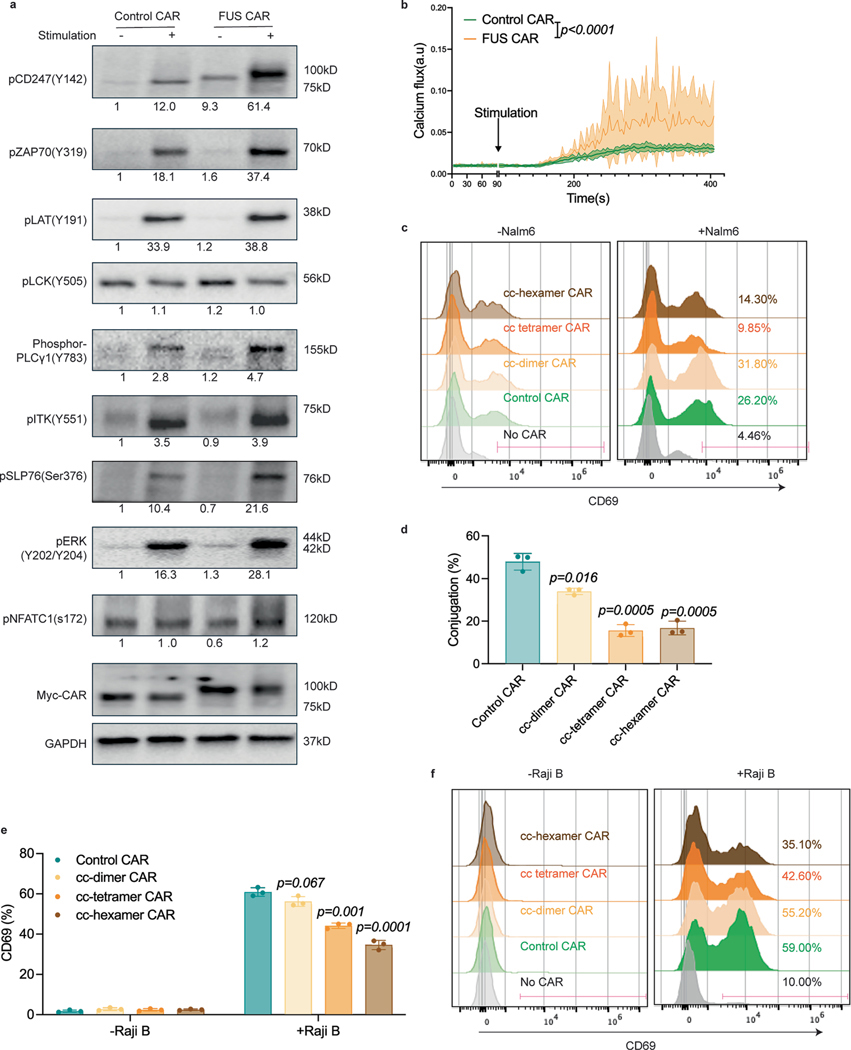
Oligomerization by coiled-coil domain reduced CAR-T activation. **a**, Signaling of activated CAR-T. Phosphorylation of CD3ζ, ZAP70, LCK, LAT, PLCγ1, SLP76, ITK, ERK, and NFATC1, together with the expression of CAR and GAPDH, were detected by Western Blot with the stimulation of anti-idiotype (Monoclonal Anti-FMC63 Antibody, 1:50) and cross-linking antibody (1.6 μg/mL). Representative blots of CAR-T generated from one donor are shown. The fold change after normalized by control CAR and corresponding CAR expression were quantified. **b**, Calcium influx in activated CAR-T was detected by flow cytometry n = 4 from 2 donors. (*p* < *0.0001* by two-way ANOVA). **c**, Representative flow cytometry plots showing CD69 expression in coiled-coil CAR-Ts. CAR-Ts were co-cultured with Nalm6 for 1 day at an E:T = 1:1. **d**, Cell-cell conjugation between CAR-T and Raji B cells with an E:T = 1:1 imaged by confocal microscopy. N = 3 technical replicates. Shown are mean ± std. (*p* value by unpaired two-sided Student’s t test). **e-f**, CD69 expression on coiled-coil CAR-Ts when engaged with Raji B cells. CAR-Ts were co-cultured with CD19-high Raji B cells for 1 day at an E:T = 10:1. CD69 expression was revealed by flow cytometry. N = 3 technical replicates. Shown on are mean ± std. (*p* value by unpaired two-sided Student’s t test). Representative flow cytometry plots were displayed.

Next, we monitored membrane-proximal signaling in CAR-Ts upon engaging with cancer cells. Following a CAR–antigen engagement, the CD3ζ domain on CAR is phosphorylated, recruiting kinase ZAP70 which further phosphorylates LAT, a key adaptor protein nucleating multiple cytosolic effectors. We cocultured CAR-Ts with CD19^low^ Raji B cells and monitored the antigen-dependent signaling kinetics by flow cytometry. With comparable phosphorylation of CD3ζ and LAT in a resting state (Extended Data Fig. 9a), we found that the FUS, EWS and TAF15 IDRs enhanced the phosphorylation of CD3ζ and LAT following CAR-T’s binding to Raji B (Fig. 5g, h and Extended Data Fig. 9b, c). Because CD3ζ and LAT are two transmembrane proteins enriched in the CAR-T synapse, we examined their phosphorylation level in the synapse by confocal microscopy. Consistent with the flow cytometry measurement, all three IDRs enhanced the phosphorylation of CD3ζ and LAT in the synapse (Fig. 5i–l and Extended Data Fig. 9d, e). It should be noted that the above assays were based on microscopy and flow cytometry, enabling us to score CAR-positive cells only to avoid the influence from variance in CAR expression on the signaling readout. However, in the cytotoxicity and cytokine production assay (Fig. 2c–i), the readouts were contributed from all cells; therefore, the variance in CAR expression had a significant influence on the readouts and needs to be taken into account when interpreting the results. In addition to CD3ζ and LAT, we compared the activation of other membrane-proximal signaling proteins between the control and FUS CAR-Ts using western blot. We found a higher phosphorylation (>1.5-fold) in ZAP70, PLCγ1, SLP76 and ERK (Extended Data Fig. 10a). Consistently, we also observed a higher calcium influx in the FUS CAR-T as compared to the control CAR-T (Extended Data Fig. 10b). Together, these data suggest that IDRs promote CAR-T activation by promoting membrane-proximal signaling pathways.

### Coiled-coil-mediated oligomerization reduces CAR-T activation

**Fig. 6 |:**
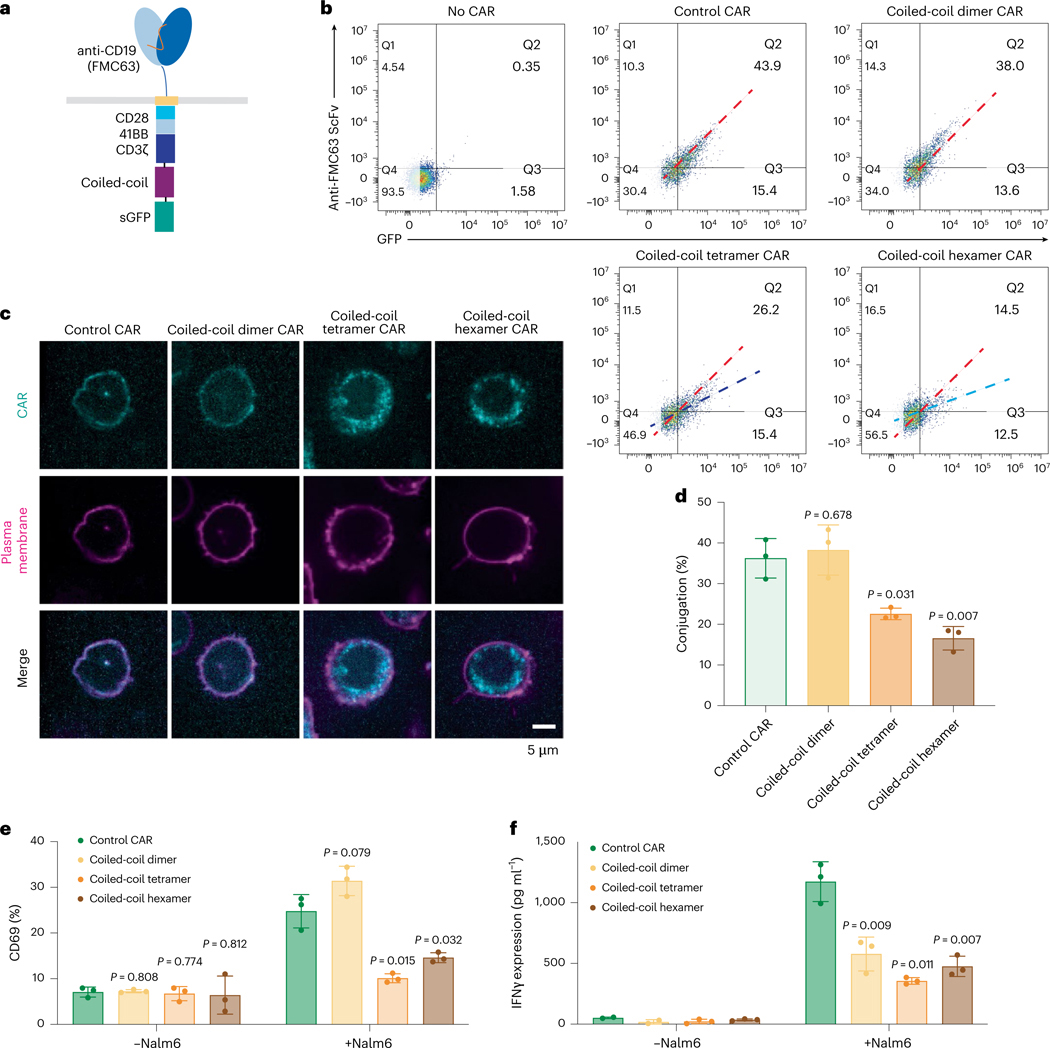
Oligomerization by coiled-coil domain reduced CAR surface localization and CAR-T activation. **a**, Schematics of the coiled-coil CD19 CAR. It contained an scFv targeting CD19 (FMC63), a CD8 hinge, a LAT transmembrane domain, an intracellular signaling domain composed of CD28, 41BB and CD3ζ and a coiledcoil domain. **b**, Expression of coiled-coil CARs detected by flow cytometry using an anti-FMC63 antibody. A GFP tag was fused on the C terminus of each CAR to monitor the total expression of CAR. The red dashed line indicates the slope of the total versus surface CAR expression of the control CAR. The dark-blue and light-blue lines indicate the slope for the expression of the coiled-coil tetramer and coiled-coil hexamer CAR, respectively. **c**, Confocal microscopy revealing the cellular localization of coiled-coil CAR shown as a cyan color. The plasma membrane is labeled by a CellMask deep red dye shown as magenta in the image. **d**, Cell–cell conjugation between CAR-T and Nalm6 cells with E:T = 1:1 imaged by confocal microscopy (*n* = 3 technical replicates). Shown are the means ± s.d. (*P* values determined using an unpaired two-sided Student’s *t*-test). **e**,**f**, Activation of coiled-coil CAR-Ts assessed by CD69 expression and IFNγ release cocultured with Nalm6 for 1 day at E:T = 1:1 (*n* = 3 technical replicates). Shown are the means ± s.d. (*P* values determined using an unpaired two-sided Student’s *t*-test).

In addition to IDR-mediated protein condensation, the coiled-coil domain is a commonly used tool to induce oligomerization of protein of interest. To test whether coiled-coil domains could promote CAR-T activation, we fused a coiled-coil domain that mediate dimerization, tetramerization or hexamerization^45,46^ to the C terminus of a CD19 CAR (Fig. 6a) and introduced these coiled-coil CARs into human primary T cells. Interestingly, whereas the coiled-coil dimer maintained a similar cell surface expression level as compared to the control CAR, the coiled-coil tetramer and hexamer CARs showed a dramatic reduction in the cell surface localization (Fig. 6b, c). Consequently, the cell–cell conjugation percentage between CAR-T and Nalm6 cells was significantly reduced in the coiled-coil tetramer and hexamer as compared to the control CAR (Fig. 6d). Consistent with this, CAR-T activation, as evaluated by CD69 expression (Fig. 6e and Extended Data Fig. 10c) and IFNγ secretion (Fig. 6f), was significantly reduced in the coiled-coil tetramer or hexamer. The reductions in cell–cell conjugation and CAR-T activation were recapitulated when using Raji B as a target cell (Extended Data Fig. 10d–f). Similar to CAR, the oligomerization-induced receptor internalization was frequently observed in other transmembrane receptors including the epidermal and fibroblast growth factor receptors^47,48^. The fact that IDRs did not affect the cell surface expression of CARs suggests that IDRs present a unique advantage to promote CAR clustering without causing enhanced receptor internalization.

## Discussion

We demonstrated that the IDRs from FUS, EWS and TAF15 promoted CAR condensation and membrane-proximal signaling without causing spontaneous activation in the absence of antigen. The FUS IDR consistently promoted antitumor effects in all three tumor models (CD19, HER2 and CD22). EWS and TAF15 promoted tumor killing in some models and their ineffectiveness in other models can be explained by a reduction in CAR expression or hyperactive signaling-induced loss of function, as discussed below.

Biomolecular condensation has been demonstrated to regulate diverse cellular signaling processes^28,49,50^. Previous work showed that condensation of TCR signaling molecules promotes membrane-proximal signaling, including LAT phosphorylation, RAS activation, actin polymerization and ERK activation^21–24,51–53^. Very recent work showed that the inclusion of a modified CD3ɛ chain into CARs promoted CAR condensation and antitumor effects^54^. Our work revealed that IDRs not previously reported to be involved in T cell signaling can promote CAR condensation and enhance the elimination of low-antigen tumors. This serves as a new strategy to improve the function of CAR-T. Importantly, these IDRs enhance the cytotoxicity of CAR-Ts without inducing signaling in the absence of antigen. Because IDRs are present in about 50% of the human proteins and display diverse chemical and material features, it is likely that we only revealed the tip of the iceberg with respect to IDRs that can be used for engineering CARs; many more IDRs are expected to be revealed to modulate the condensation, antigen binding and conformation of CARs.

In this work, we demonstrated that several well-characterized IDRs including those from FUS, EWS and TAF15, promote the condensation of CARs on the T cell membranes, which leads to enhanced cytotoxicity toward low-antigen-expressing cancer cells. Because none of these three IDR-containing proteins have been reported with a function in T cell signaling or cytotoxicity, our strategy can serve as an new way to enhance the low signaling efficiency of CAR-T, in combination with other strategies, including selecting specific transmembrane or cosignaling domains^12,55,56^, adding new signaling binding motifs^19^, replacing the intracellular part with that from TCR (HIT)^57^, dual targeting by CAR together with chimeric costimulatory receptors^43^ or implementing other signaling pathways^58^, to achieve synergistic effects. It is worth mentioning that, although we focused on the condensation function of IDRs, these IDRs also bind many other intracellular factors^36^; therefore, we do not exclude the possibility that IDRs can recruit cytosolic factors to modulate T cell signaling.

Although all the three IDRs that promoted CAR condensation enhanced membrane-proximal signaling including pCD3ζ and pLAT, this beneficial effect in initial signaling was not always translated into enhanced cytotoxicity. For example, the TAF15 CD19 CAR-T did not show an enhanced cytotoxicity in vitro. We reasoned that this was because of the lower expression of TAF15 CD19 CAR-T as compared to the control CD19 CAR-T. It is worth noting that, when quantifying pCD3ζ and pLAT, we only scored these CAR-positive cells to minimize the influence from the variance from CAR expression. On the other hand, TAF15 HER2 CAR-T was expressed at a similar level to the control HER CAR-T and the TAF15 HER2 CAR-T induced a higher cytotoxicity than the control CAR. This suggests that TAF15 per se promotes cytotoxicity but the level of penetrance of initial signaling benefits into cytotoxicity is influenced by CAR expression level.

It is worth noting that the three IDRs displayed different features to affect CAR-T function. FUS mildly promoted condensation and signaling, which resulted in a consistent improvement in antitumor effects in all tumor models tested. TAF15 would bring a benefit if a decent expression level of CAR could be obtained. EWS, which contains the highest number of tyrosines, strongly promoted condensation and signaling and is, thus, suitable for improving CARs with weak signaling (such as the CD22 CAR) but not CARs with efficient signaling (such as the CD19 CAR). This could be explained by a bell-shaped relationship between the signaling strength and antitumor effects as depicted in the ‘peak model’ (ref. 30). According to this model, superstrong signaling could lead to premature exhaustion and loss of function of CAR-T. Indeed, we observed that EWS CAR-T enhanced the tumor control at early time points (day 7) but this effect was lost at later time points (after day 14) (Fig. 2l). The above knowledge is instructional for choosing IDRs to design future CARs.

Lastly, we revealed an interesting difference between the outcomes of different ways to induce CAR clusters. IDR-induced CAR condensation enhanced CAR phosphorylation and CAR-T activation, whereas coiled-coil domains reduced cell surface expression of CAR and suppressed CAR-T activation. One of the major differences between IDRs and coiled-coils is that IDRs promote self-assembly of CAR-Ts through weak interactions and the condensates maintain liquid-like or gel-like properties. In contrast, coiled-coil domains induced stable oligomerization, which could either affect the trafficking of CAR to the cell surface or trigger instant receptor internalization. Our work suggests that IDRs present a unique advantage in promoting the assembly of CAR into a higher-order structure without compromising their cell surface localization.

### Online content

Any methods, additional references, Nature Portfolio reporting summaries, source data, extended data, supplementary information, acknowledgements, peer review information; details of author contributions and competing interests; and statements of data and code availability are available at https://doi.org/10.1038/s41589–025-02031-x.

## Methods

A list of reagents used in this study can be found in Supplementary Table 1.

### Plasmids and lentivirus

DNA fragments encoding CAR or antigen molecules were inserted into a pHR lentiviral vector with an SFFV promoter and a WPRE terminator. HEK293T cells, maintained in DMEM medium supplemented with 10% FBS and a glutamine–penicillin–streptomycin mix, were cotransfected with the pHR plasmids and second-generation lentiviral packaging plasmids pMD2.G and psPAX2 (Addgene, plasmids 12259 and 12260) using Genejuice transfection reagent (EMD Millipore, 70967–3). Then, 48 h after transfection, cell culture media containing viral particles were harvested, centrifuged and filtered through 0.45-μm-pore-size filters.

### Generation of CAR-T cells

Pan T cells were isolated from PBMCs (Zenbio, SER-PBMC-200) from healthy donors using EasySep human T cell isolation kit (Stem Cell, 17951). T cell proliferation was stimulated with Human T activator CD3/CD28 Dynabeads (Thermo Fisher, 11161D). Cells were cultured in RPMI-1640 supplemented with 10% FBS, 50 nM 2-mercaptoethanol, 300 U per ml IL-2 (PeproTech, 200–02). Then, 2 days after stimulation, the cells were infected with fresh lentivirus by spinoculation at 800*g* for 90 min at 32 °C. Half of the cell culture media were changed with fresh T cell culture medium 24 h after infection. Then, 5 days after infection, Dynabeads were removed and CAR-T cells were resuspended in a fresh culture medium. The medium was exchanged every 2 days thereafter.

### Generation of cancer cell lines

The Nalm6, Raji B and K562 cells were cultured in RPMI-1640 supplemented with 10% FBS and a glutamine–penicillin–streptomycin mix. HT29 cells were cultured in DMEM supplemented with 10% FBS and a glutamine–penicillin–streptomycin mix. The Nalm6 CD19^high^ and CD19^low^ cell lines expressing GFP and luciferase were kindly provided by the R. Majzner lab at Stanford University. The Raji B CD19^high^ and CD19^low^ cells were generated by sorting the wild-type Raji B cells for high and low CD19 expression level. The K562 HER2^high^ and HER^low^ cells were generated by infecting K562 cells with lentivirus encoding the wild-type HER2, followed by single-cell sorting. The number of antigen molecules per cell was quantified using the BD Quantibrite PE phycoerythrin fluorescence quantitation kit (BD Bioscience, 340495) by flow cytometry. Luciferase-expressing cells were generated by infecting cancer cells with a lentiviral plasmid expressing luciferase–GFP or luciferase–mCherry, followed by fluorescence-activated cell sorting to generate stable cell lines.

### In vitro cytotoxicity assay

Cytotoxicity was measured by luciferase assay. Cancer cells expressing luciferase were resuspended in RPMI-1640 medium supplemented with 10% FBS and mixed with CAR-T cells at an effector-to-target (E:T) ratio of 0.3:1 to 10:1. After 24 h of incubation at 37 °C, cells were collected and washed with PBS. Cell pellets were lysed in a lysis reagent (Promega, 1531). The luminescence of lysates was detected by the luciferase assay system (Promega, E1500) and analyzed using a plate spectrophotometer. The spontaneous release control was set up using cancer cells alone. Cell lysis (%) = (1 − (experimental readout − spontaneous readout)/spontaneous readout) × 100.

### Cytokine production

CAR-T cells and cancer cells were cocultured at indicated E:T ratios for 24 h at 37 °C. The supernatant was collected for cytokine measurement using ELISA kits (IL-2 ELISA kit, BioLegend, 431801; IFNγ ELISA kit, BioLegend, 430101; TNF ELISA kit, BioLegend, 430204) or using a LEGENDplex human CD8/NK panel kit (741186), according to the manufacturer’s instructions.

### Flow cytometry

To determine the cell surface expression, cells were collected and blocked with an anti-human Fc receptor-binding inhibitory antibody in the staining buffer (PBS with 2% FBS and 1 mM EDTA) for 15 min at 4 °C and then further incubated with individual antibodies in the staining buffer for 30 min on ice. The stained cells were washed twice with the staining buffer before sending for flow cytometry analysis. To determine the intracellular expression of targets of interest, cells were collected and fixed with fixation/permeabilization solution (554714) for 15 min on ice and blocked in the staining buffer for 30 min on ice, followed by antibody staining. To characterize T cells in the mice blood, blood was drawn from a tail cut and diluted into PBS supplemented with 3 mM EDTA. Red blood cells were lysed with a red blood cell lysis buffer. The remaining cells were stained as described above, followed by flow cytometry. To characterize tumor-infiltrating T cells, tumors were dissected and digested with RPMI-1640 medium containing 0.5 mg ml^−1^ Collagenase P and 1 μg ml^−1^ DNase per 100 mg of tumor tissues for 30 min at 37 °C on a shaker. The digested tumor tissues were further homogenized and passed through a 40-μm strainer, followed by centrifugation to collect cell samples. Cells were further stained and analyzed by flow cytometry as described above.

### Cell conjugation, mechanical strength and CD45 exclusion

To determine cell–cell conjugation percentage, Nalm6 CD19^high^ cells expressing mCherry were cocultured at a 1:1 ratio with primary CAR-T cells in RPMI-1640 medium supplemented with 20 mM HEPES (pH 7.4) for 15 min at 37 °C. Cells were fixed in 4% paraformaldehyde (Santa Cruz Biotechnology, 30525–89-4) for 15 min at room temperature. The cells were washed with PBS and resuspended in RPMI-1640 medium supplemented with 20 mM HEPES and imaged by confocal microscopy. Cell conjugation percentage was calculated by dividing the number of tumor-cell-associated GFP^+^ CAR-T cells by the number of total GFP^+^ CAR-T cells.

The mechanical strength of the synapse was measured by the Lumicks Z-movi following the manufacturer’s instructions. Briefly, Z-movi-compatible acoustofluidic chips were coated with poly(L-lysine) for 1 h at 37 °C to attach a monolayer of Nalm6 CD19^low^ cells. Cell Trace far-red-labeled CAR-T cells were incubated on the tumor cell monolayer for 10 min, followed by the application of a ramping acoustic force. Cell detachment was analyzed using ImageJ and R and the bound cell percentage was calculated.

To determine CD45 exclusion from the synapse, Raji B CD19^low^ cells expressing mCherry were cocultured at a 1:1 ratio with primary CAR-T cells in RPMI-1640 medium supplemented with 20 mM HEPES (pH 7.4) for 15 min at 37 °C. Cells were fixed in 4% paraformaldehyde (Santa Cruz Biotechnology, 30525–89-4) for 15 min at room temperature. Cells were stained with an anti-CD45–allophycocyanin antibody (BioLegend, 304012) for 30 min on ice. Then, the cells were washed with PBS and resuspended in RPMI-1640 medium supplemented with 20 mM HEPES and imaged by confocal microscopy. To quantify CD45 exclusion in the synapse, the line scan function was used to measure the intensities of CD45 in the synapse between CAR-T and target tumor cells. Exclusion percentage (%) = (1 − intensity of CD45 in CAR zone/intensity of CD45 out of CAR zone) × 100.

### Calcium influx assay

CAR-T cells were labeled with indo-1 (2 μM) for 30 min at 37 °C and washed with PBS for another 30 min at 37 °C. CAR-T cells were stimulated with anti-idiotype (monoclonal anti-FMC63 antibody, 1:50) and crosslinking antibody (1.6 μg ml^−1^) at 37 °C for 2 min and calcium concentration was measured as an emission ratio (390 nm:475 nm) emission ratio every 1 s using the flow cytometer Cytek Aurora. Calcium signal was calculated as the fluorescence at 390 nm (Ca^2+^-bound) divided by the fluorescence at 475 nm (Ca^2+^-free). The data were smoothened by averaging three constitutive time points before being plotted.

### CAR signaling characterization

For microscopy based CAR signaling, CAR-T cells were resuspend in image medium (RPMI-1640, no phenol red supplementation, with 20 mM HEPES pH 7.4) and cocultured with cancer cells at E:T = 1:1 for the indicated time at 37 °C, followed by fixation and permeabilization on ice for 15 min and staining with individual phosphorylated antibodies before sending for flow cytometry analysis or confocal microscopy. A single focal plane that aligns with the maximum of cell–cell conjugates was imaged to minimize photobleaching from *z*-stack capturing.

For immunoblotting based CAR signaling, CAR-T cells were stimulated with anti-idiotype (monoclonal anti-FMC63 antibody, 1:50) and crosslinking antibody (1.6 μg ml^−1^) at 37 °C for 2 min. After stimulation, total protein was extracted with protein extraction buffer (Millipore, 70584–3). Cell lysates were heated for 10 min at 95 °C with 4× Laemmli sample buffer (Bio-Rad, 1610747). The supernatants were processed for SDS–PAGE, followed by a transfer onto a polyvinylidene difluoride membrane (Bio-Rad, 1620177). The membrane was blocked with Tris-buffered saline with Tween-20 containing 5% nonfat milk for 1 h at room temperature and blotted with indicated primary antibodies overnight at 4 °C. The next day, the membrane was further blotted with horseradish peroxidase (HRP)-conjugated secondary antibody for 1 h at room temperature. Target proteins were detected with a chemiluminescent HRP substrate (Thermo Fisher Scientific, 34577) and visualized by a Bio-Rad ChemiDoc imaging system (Bio-Rad). Images were quantified by ImageJ.

### Mouse tumor xenograft

Mice were housed in pathogen-free conditions and cared for in accordance with US National Institutes of Health (NIH) guidelines and all procedures were approved by the Yale University Animal Care and Use Committee. To generate liquid cancer models, 1 million Raji B or Naml6 cells in 100 μl of PBS were intravenously injected into NSG mice(~6 weeks old) through the tail vein. Then, 3 days later, 8 million CAR-T cells in 100 μl of PBS were injected through the tail vein. In vivo imaging of bioluminescence was performed to monitor tumor growth. Blood was drawn at indicated time points after CAR-T treatment for flow cytometry analysis of CAR-T. To generate solid tumor models, 2.5 million HT29 cells in 100 μl of PBS were subcutaneously injected into the right flank of NSG mice. Then, 8 days later, when the tumor became palpable, 6 or 12 million CAR-T cells in 100 μl of PBS were injected through the tail vein, which was followed by a second-dose treatment of the same number of CAR-T cells after 5 days. Tumor growth was measured with calipers every 2 days and the size was calculated as one half of the product of perpendicular length and square width in cubic millimeters (volume = 1/2 × L × W × W). Mice were killed when the tumor size exceeded 2,000 mm^3^. Blood was collected and tumors were dissected to quantify tumor-infiltrating T cells.

### Microscopy

TIRF and confocal microscopy was performed on a Nikon Ti2-E inverted motorized microscope stand equipped with a motorized stage with a stage-top Piezo, Nikon H-TIRF, Yokogawa CSU-X1 spinning disk confocal, Agilent laser combiner with four lines (405, 488, 561 and 640 nm) and scientific complementary metal–oxide–semiconductor camera Photometrics Prime 95B. Images were acquired using Nikon Elements software.

### Image analysis

Microscopy images were analyzed in Fiji (ImageJ). The same brightness and contrast were applied to images within the same panels for the same batch. CAR condensation was quantified as normalized variance, which is equal to the square of s.d. divided by the mean with an s.d. threshold of 3–10 depending on the CAR expression level of each batch. Cell conjugation percentage was calculated by dividing the number of tumor-cell-associated GFP^+^ CAR-T cells by the number of total GFP^+^ CAR-T cells. CAR activation was calculated by dividing the intensities of pCD3ζ (Y142) or pLAT (Y171) in the synapse by the intensities of CAR–GFP.

### Single-cell RNA-seq

In the CD19 CAR-T cancer model, mouse blood was collected from the tail vein on day 43 after CAR-T cell treatment. Mouse (m)PBMCs were isolated after red blood cells were lysed. Anti-human CD3 and CD45 antibodies were used to identify the T cell population. T cells freshly sorted from mPBMCs were subjected to RNA-seq library preparation by Chromium next-GEM (gel bead-in emulsion) single-cell 3′ reagent kits v3.1 (dual index). Briefly, in step 1, after preparing the fresh master mix, single-cell suspension (10,000 cells per sample) and gel beads, GEMs were generated by combining the prepared master mix containing cells, gel beads and partitioning oil onto chromium next-GEM chip G in the chromium controller. Immediately after GEM generation, GEMs were transferred to PCR tubes to run GEM-RT incubation. In step 2, after GEM-RT, cleanup was performed with Dynabeads, which was followed by complementary DNA (cDNA) amplification and cDNA cleanup with SPRlselect. The purified cDNA was quantified using BioAnalyzer. In step 3, cDNA amplicon size was further optimized by enzymatic fragmentation and size selection. P5, P7, i7 and i5 sample indices and TruSeq read 2 were added by end pair, poly(A) tailing, adaptor ligation and PCR. A quality control step was run after library construction. In step 4, the chromium single-cell 3′ gene expression dual-index library containing standard Illumina paired-end constructs (beginning and ending with P5 and P7) were sequenced by a Hiseq 2500 RapidRun. The 16-bp 10x barcode and 12-bp unique molecular identifier were encoded in read 1. Read 2 was used to sequence the cDNA fragment. The i7 and i5 index sequences were incorporated as the sample index reads.

Single-cell RNA-seq reads were processed using Cell Ranger software (10x Genomics, v.7.2.0) with standard parameters and aligned to the human reference transcriptome (GRCh38). Gene expression comparison was performed using Loupe Browser (10x Genomics, v.7.0.0) and cluster annotation was performed manually on the basis of marker gene expression.

### Statistical analysis

An unpaired two-sided Student’s *t*-test or Mann–Whitney *U*-test was used to assess significance. A two-way analysis of variance (ANOVA) was used for comparison analysis when evaluating more than two groups or more than one effect. A *P* value < 0.05 was considered significant. Data were analyzed using GraphPad Prism. The statistical details for each experiment are provided in the associated figure legends.

## Extended Data

## Supplementary Material

Table S1

**Supplementary information** The online version contains supplementary material available at https://doi.org/10.1038/s41589-025-02031-x.

## Data Availability

The data supporting the findings of this study are available in the paper and its Supplementary Information. The single-cell sequencing data can be anonymously accessed from the National Center for Biotechnology Information Gene Expression Omnibus under accession number GSE272224. Raw flow cytometry data and raw image data are available through the Yale Dataverse repository (https://doi.org/10.60600/YU/8UUAHW, https://doi.org/10.60600/YU/REY5VG, https://doi.org/10.60600/YU/H2SZ02 and https://doi.org/10.60600/YU/E6ZDOY). Source data are provided with this paper.
