# Supplementary material for: IDR-induced CAR condensation improves the cytotoxicity of CAR-Ts against low-antigen cancers
Source: Nat Chem Biol. 2025 Sep 29;22(3):379–91. doi: 10.1038/s41589-025-02031-x (PMC12825998; doi:10.1038/s41589-025-02031-x)
Supplement: Supplementary file 1 — Supplementary figures showing individual datasets for the corresponding figures and Extended Data figures. Reagents table including cell lines, plasmids, IDR sequence, antibodies and other reagents. [file 41589_2025_2031_MOESM1_ESM.pdf]

nature chemical biology

Supplementary information https://doi.org/10.1038/s41589-025-02031-x

IDR-induced CAR condensation improves the cytotoxicity of CAR-Ts against low-antigen cancers

In the format provided by the authors and unedited

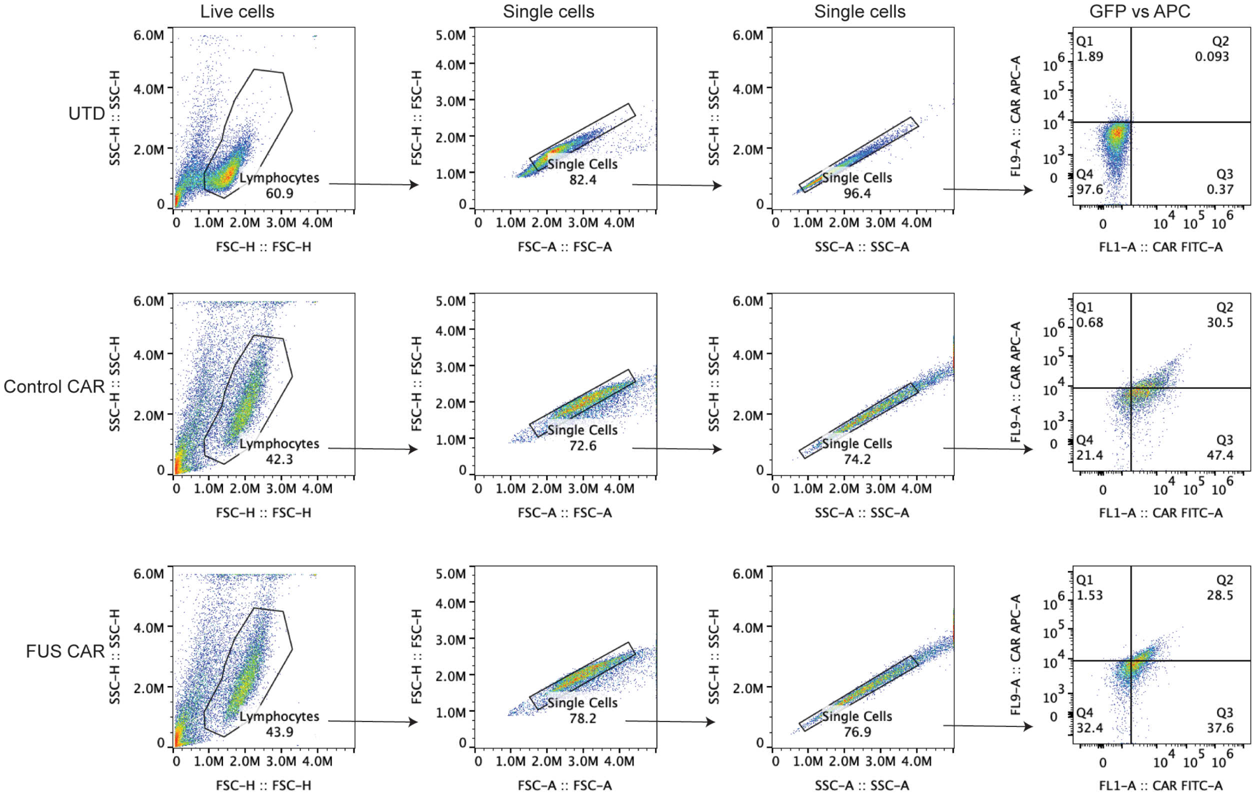

Supplementary Figure 1 | Flow cytometry gating strategy (Figure 1b)

The gating strategy starts from gating alive cells based on cell size (X-axis is FSC-H and Y-axis is SSC-H), followed by single cell gating based on the major population in the plot of FSC-A (X-axis) vs FSC-H (Y-axis), the second time single cell gating based on the major population in the plot of SSC-A (X-axis) vs SSC-H(Y-axis). Total CAR and surface CAR double positive population is FITC (GFP, X-axis) vs APC (Y-axis).

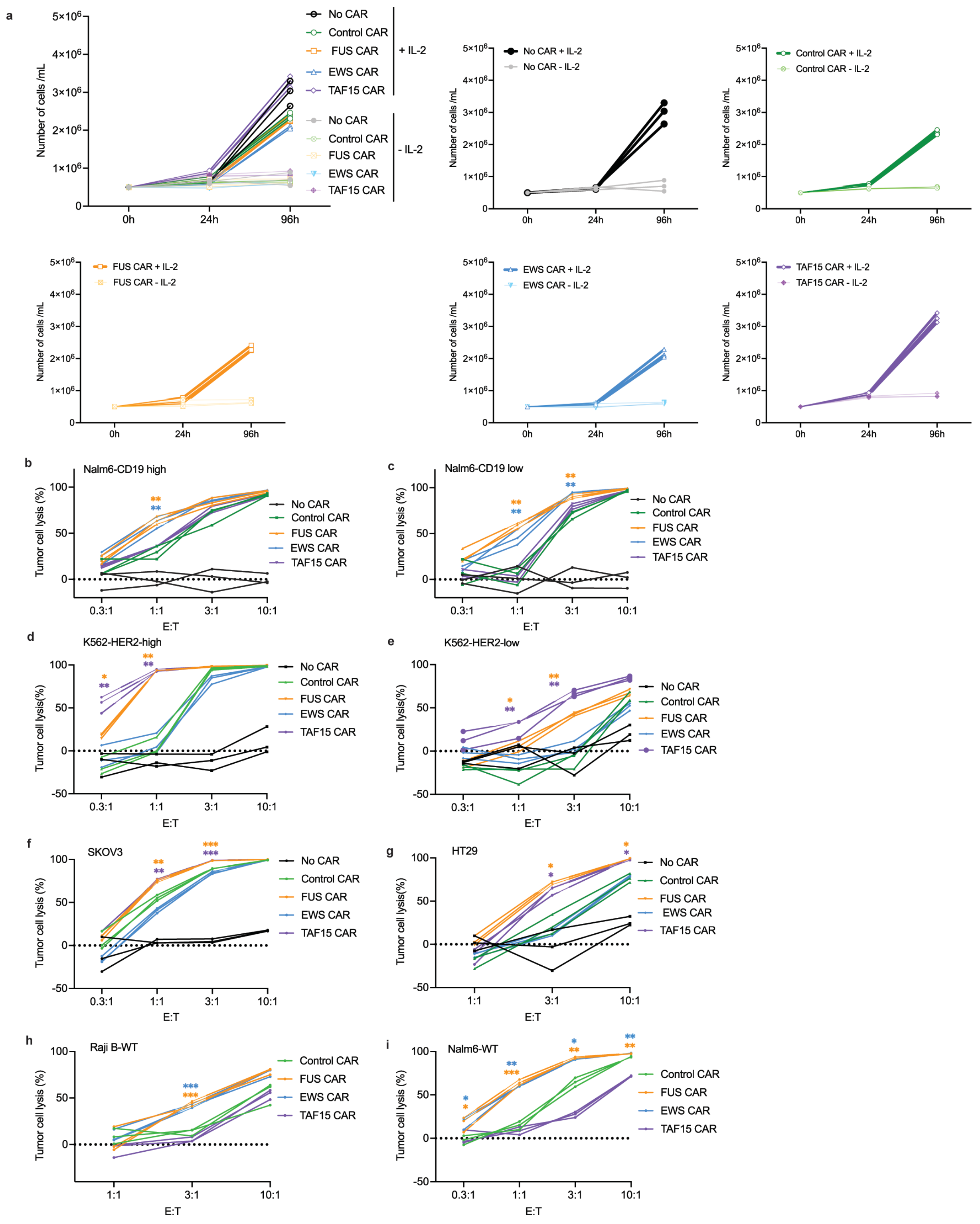

Supplementary Figure 2 | Individual proliferation and cytotoxicity curve

a, Individual proliferation curve for Figure 1e.

b-c, Individual cytotoxicity curve for Figure 2c-2d.

d-g, Individual cytotoxicity curve for Figure 3e-3h.

h-i, Individual cytotoxicity curve for Figure 4d-4e.

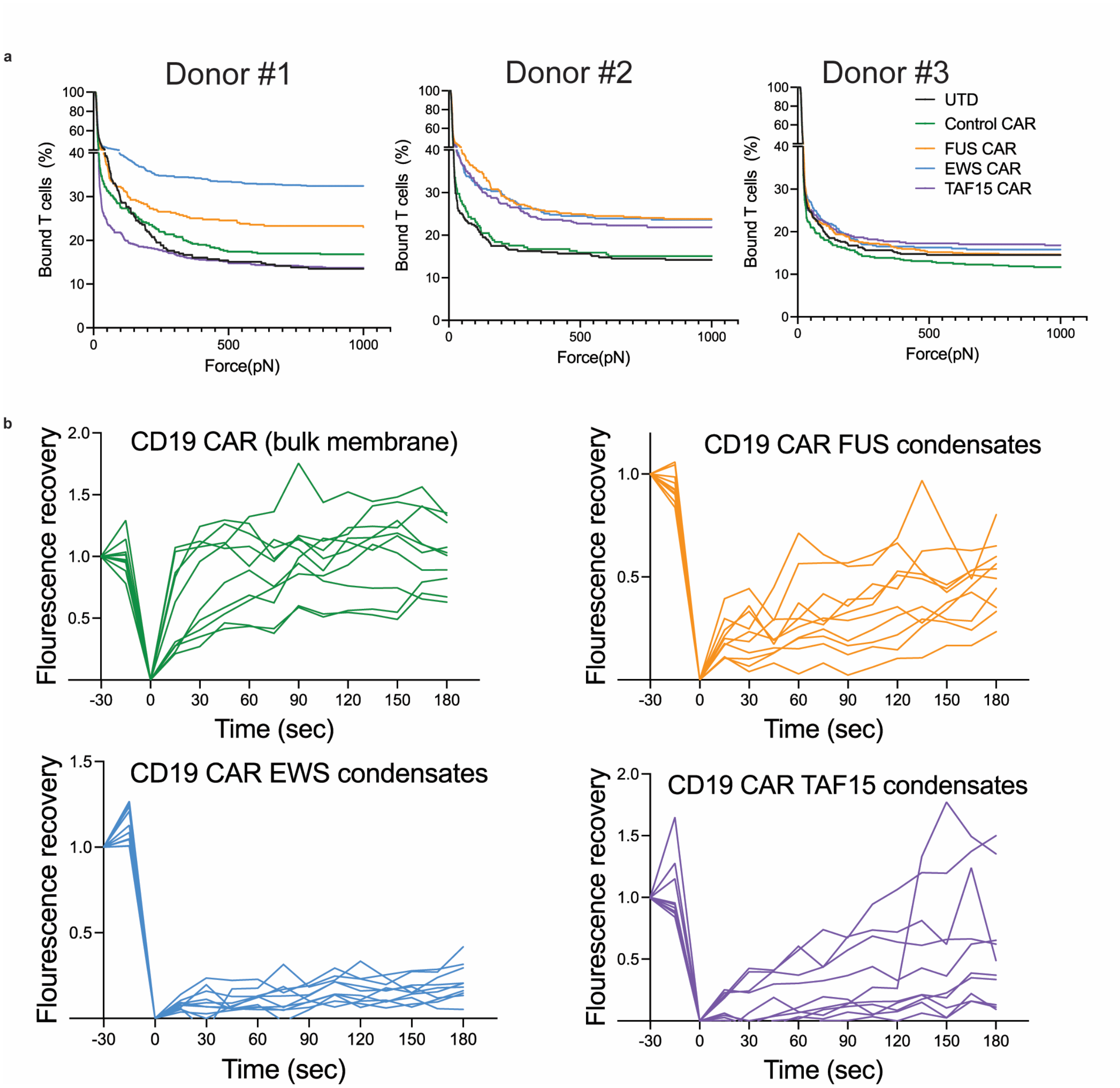

Supplementary Figure 3 | Individual cell-cell interaction curve and FRAP dataset
a, Individual dataset for rupture forces to detach CD19 control and IDR CAR-T from Nalm6-CD19 low cells by z-movi from Lumicks for Figure 5b.
b, Individual FRAP dataset for Extended Data Figure 1e.

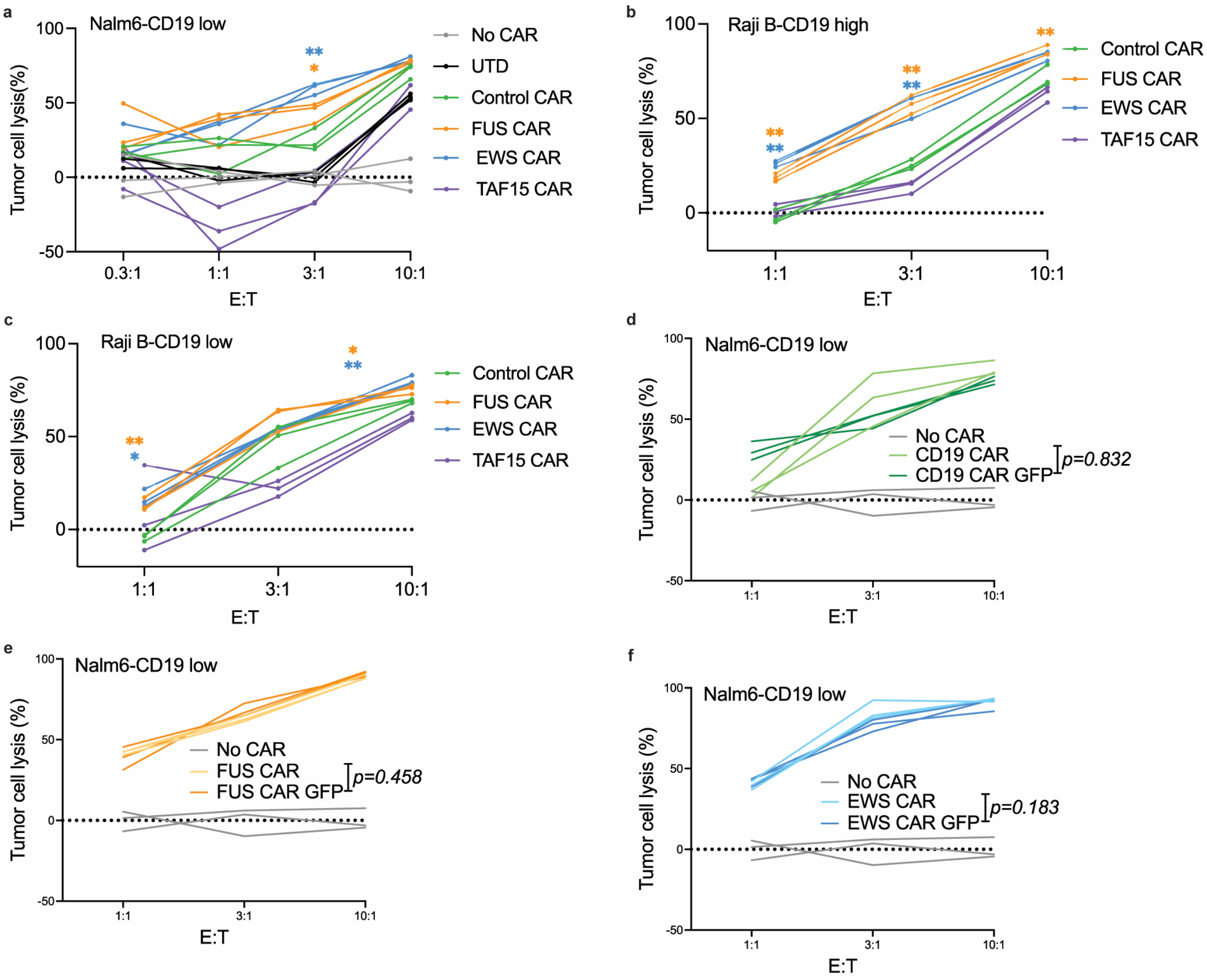

Supplementary Figure 4 | Individual cytotoxicity curve of CD19 CAR

a, Individual cytotoxicity curve for Extended Data Figure 2b.

b-c, Individual cytotoxicity curve for Extended Data Figure 2d-2e.

d-f, Individual cytotoxicity curve for Extended Data Figure 2k-2m.

g, Individual cytokine panel data for Extended Data Figure 2f.

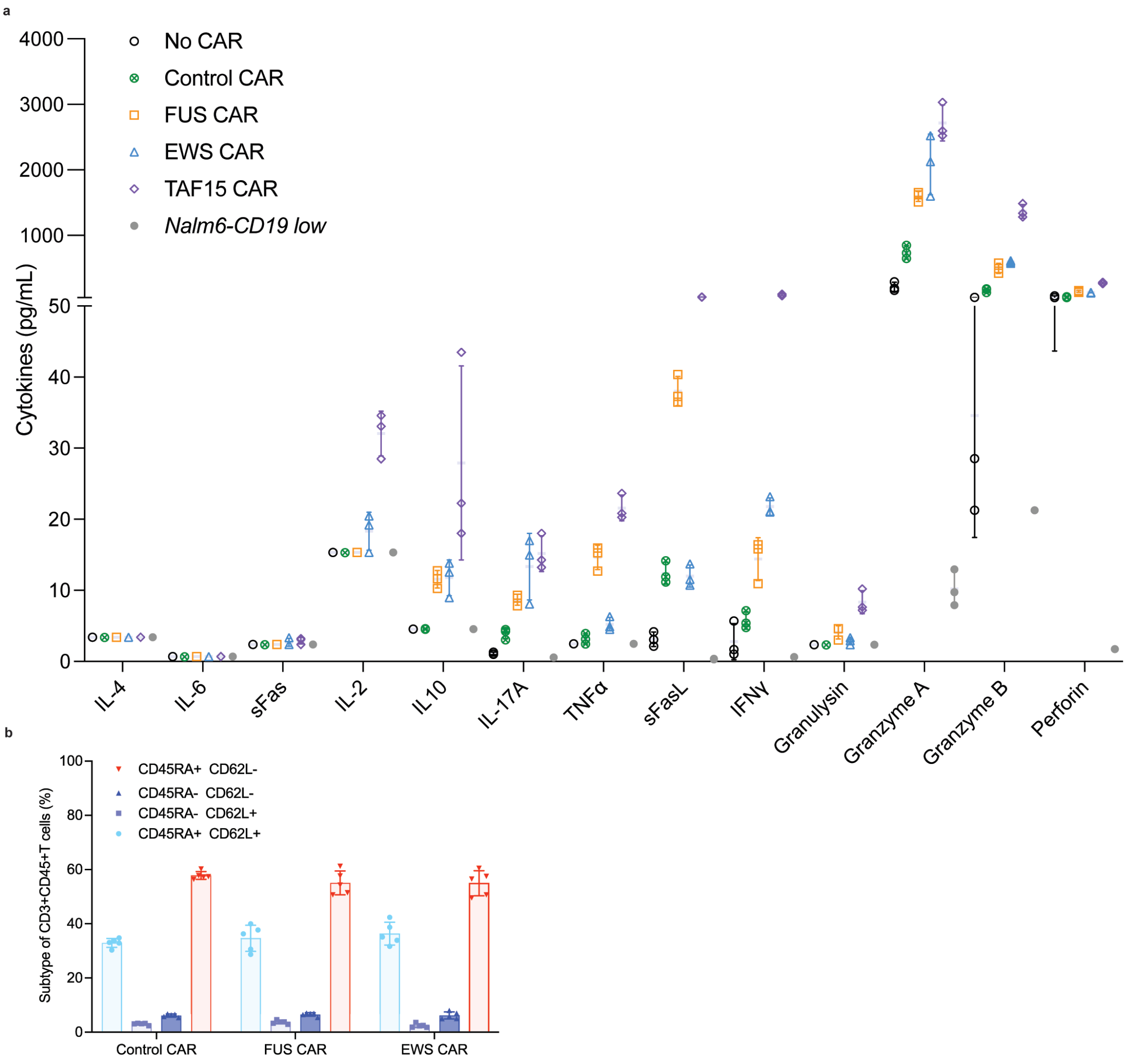

Supplementary Figure 5 | Individual CD19 CAR cytokine panel and in vivo T cell subtype dataset

a, Individual cytokine panel data for Extended Data Figure 2f.

b, Individual in vivo T cell differentiation for Extended Data Figure 4i.

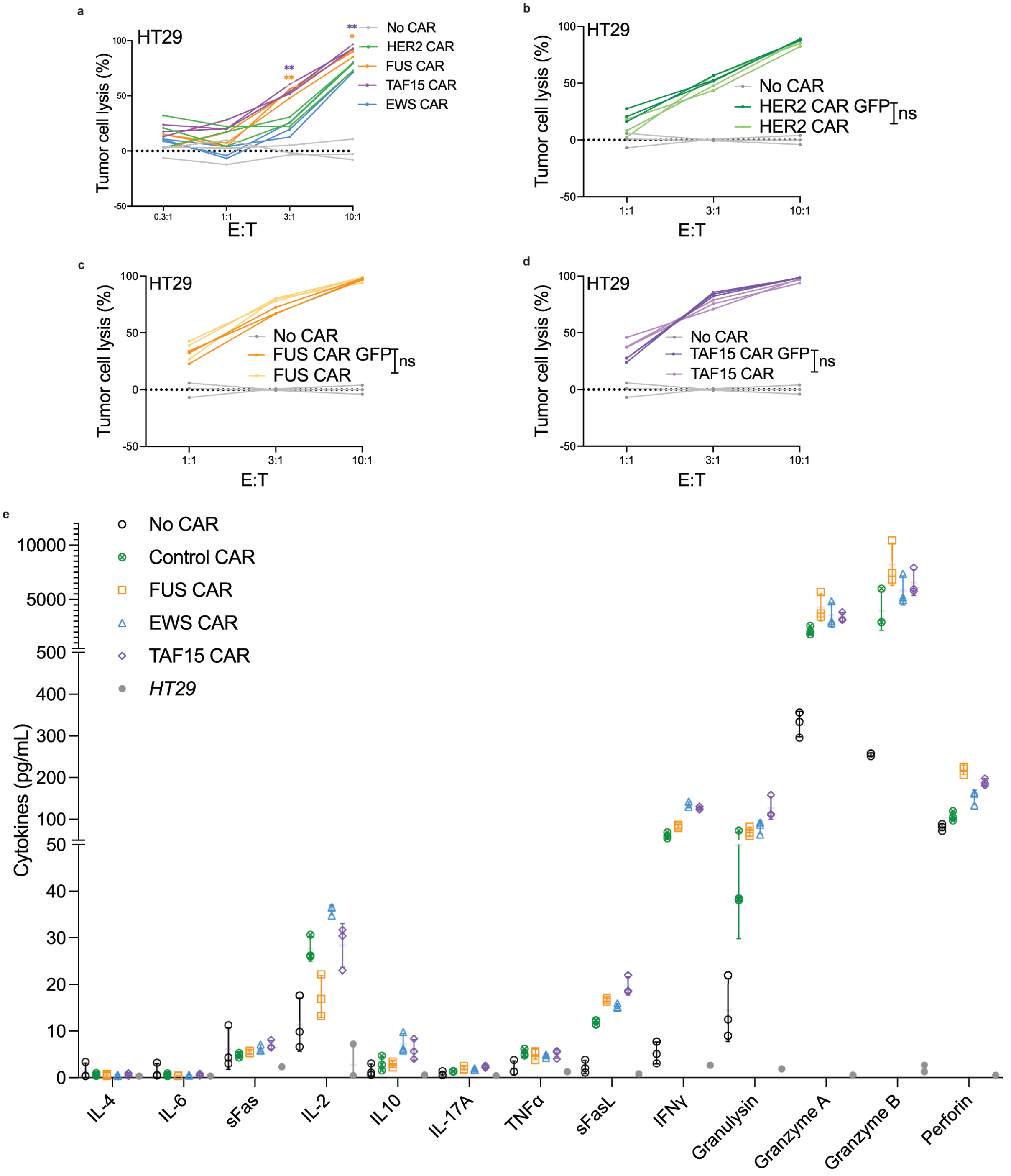

Supplementary Figure 6 | Individual cytotoxicity curve and cytokine panel dataset for HER2 CAR

a, Individual cytotoxicity curve for Extended Data Figure 6b.

b-d, Individual cytotoxicity curve for Extended Data Figure 6h-6j.

e, Individual cytokine panel data for Extended Data Figure 6c

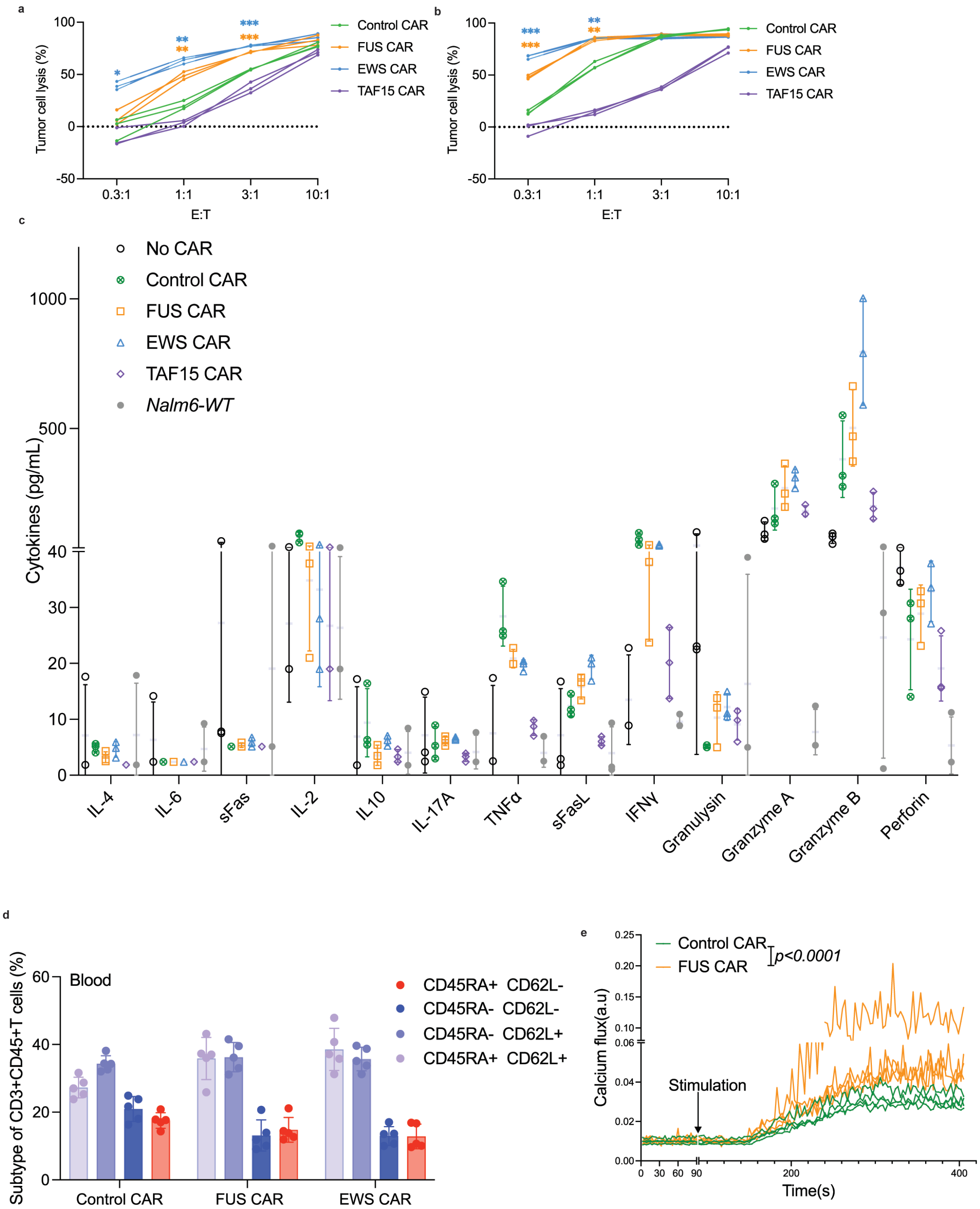

Supplementary Figure 7 | Individual cytotoxicity curve and cytokine panel dataset CD22 CAR

a-b, Individual cytotoxicity curve for Extended Data Figure 8b-8c.

c, Individual cytokine panel data for Extended Data Figure 8d.

d, Individual in vivo T cell differentiation for Extended Data Figure 8f.

e, Individual calcium flux dataset for Extended Data Figure 10b.

Supplementary Table 1. Reagents

Cell lines

Cell lines	Source	Additional infoamation
HEK293T	UCSF CCF	For lentivirus production
Raji B-ffLuc2-mCherry	UCSF CCF	For testing CD22 CAR
Raji B-CD19 high-ffLuc2-mCherry	This study	For testing CD19 CAR
Raji B-CD19 low-ffLuc2-mCherry	This Study	For testing CD19 CAR
Nalm6-CD19 high-ffLuc2-GFP	Robbie Majzner Lab	For testing CD19 CAR
Nalm6-CD19 low-ffLuc2-GFP	Robbie Majzner Lab	For testing CD19 CAR
Nalm6-ffLuc2-GFP	Sidi Chen Lab	For testing CD22 CAR
Nalm6-CD22 high-ffLuc2-mCherry	This Study	For testing CD22 CAR
Nalm6-CD22 low-ffLuc2-mCherry	This Study	For testing CD22 CAR
K562	Jun Lu Lab	
K562-HER2 high-ffLuc2-mCherry	This study	For testing CD22 CAR
K562-HER2 low-ffLuc2-mCherry	This study	For testing CD22 CAR
SKOV3	Elena Ratner Lab	
SKOV3-ffLuc2-mCherry	This study	For testing CD22 CAR
HT29	Sidi Chen Lab	
HT29-ffLuc2-mCherry	This Study	For testing CD22 CAR

Plasmids

1	pMD2.G	Vale Lab	Viral packaging
2	poPAX	Vale Lab	Viral packaging
3	pHR-mCherry-CAAX	This study (XSB473)	Lentiviral vector
4	pFUGW-FerH-ffLuc2-mCherry	This study (XSB887)	Lentiviral vector
5	pFUGW-FerH-ffLuc2-GFP	This study (XSB886)	Lentiviral vector
6	pHR-CD19-mCherry	This study (XSB875)	Lentiviral vector
7	pHAGE-ERBB2 (HER2)	This study (XSB1100)	Lentiviral vector
8	pHR NdeI-SP-CD19 ScFv-BamHI-myc-CD8 Stalk-CD8 TM-NruI-CD28-41BB-CD3z cyto-speI-sGFP	This study (XSB848)	Lentiviral vector
9	pHR NdeI-SP-CD19 ScFv-BamHI-myc-CD8 Stalk-CD8 TM-NruI-CD28-41BB-CD3z cyto-speI-FUS 1-214-sGFP	This study (XSB1261)	Lentiviral vector
10	pHR NdeI-SP-CD19 ScFv-BamHI-myc-CD8 Stalk-CD8 TM-NruI-CD28-41BB-CD3z cyto-speI-TAF15 1-180-sGFP	This study (XSB1315)	Lentiviral vector
11	pHR NdeI-SP-CD19 ScFv-BamHI-myc-CD8 Stalk-CD8 TM-NruI-CD28-41BB-CD3z cyto-speI-EWS 28-262-sGFP	This study (XSB1316)	Lentiviral vector

12	pHR NdeI-SP-CD19 ScFv-BamHI-myc-CD8 Stalk-CD8 TM-NruI-CD28-41BB-CD3z cyto-speI-Nup98 1-340-sGFP	This study (XZB101)	Lentiviral vector
13	pHR NdeI-SP-CD19 ScFv-BamHI-myc-CD8 Stalk-CD8 TM-NruI-CD28-41BB-CD3z cyto-TDP43 266-414 speI-sGFP	This study (XSB929)	Lentiviral vector
14	pHR NdeI-SP-CD19 ScFv-BamHI-myc-CD8 Stalk-CD8 TM-NruI-CD28-41BB-CD3z cyto-10xIDR-speI-sGFP	This study (XSB930)	Lentiviral vector
15	pHR NdeI-SP-CD19 ScFv-BamHI-myc-CD8 Stalk-CD8 TM-NruI-CD28-41BB-CD3z cyto-speI-FUS 1-214	This study (XSB1227)	Lentiviral vector
16	pHR NdeI-SP-CD19 ScFv-BamHI-myc-CD8 Stalk-CD8 TM-NruI-CD28-41BB-CD3z cyto-speI-TAF15 1-180	This study (XSB1228)	Lentiviral vector
17	pHR NdeI-SP-CD19 ScFv-BamHI-myc-CD8 Stalk-CD8 TM-NruI-CD28-41BB-CD3z cyto-speI-EWS 28-262	This study (XSB1229)	Lentiviral vector
18	pHR NdeI-SP-CD22 RFB4 ScFv-BamHI-myc-CD8 Stalk-CD8 TM-NruI-CD28-CD3z cyto-speI-sGFP	This study (XSB1176)	Lentiviral vector
19	pHR NdeI-SP-CD22 RFB4 ScFv-BamHI-myc-CD8 Stalk-CD8 TM-NruI-CD28-CD3z cyto-speI-FUS 1-214	This study (XSB1179)	Lentiviral vector
20	pHR NdeI-SP-CD22 RFB4 ScFv-BamHI-myc-CD8 Stalk-CD8 TM-NruI-CD28-CD3z cyto-speI-EWS 28-262	This study (XSB1226)	Lentiviral vector
21	pHR NdeI-SP-CD22 RFB4 ScFv-BamHI-myc-CD8 Stalk-CD8 TM-NruI-CD28-CD3z cyto-speI-TAF15 1-180	This study (XSB1226)	Lentiviral vector
22	pHR NdeI-SP- HER2 ScFv -BamHI-myc-CD8 Stalk-CD28 TM-NruI-CD28-41BB-CD3z cyto-sGFP	This study (XSB1094)	Lentiviral vector
23	pHR NdeI-SP-HER2 ScFv -BamHI-myc-CD8 Stalk-CD28 TM-NruI-CD28-41BB-CD3z cyto-speI-TAF15 1-180	This study (XSB1177)	Lentiviral vector
24	pHR NdeI-SP-HER2 ScFv-BamHI-myc-CD8 Stalk-CD28 TM-NruI-CD28-41BB-CD3z cyto-speI-FUS 1-214	This study (XSB1178)	Lentiviral vector
25	pHR NdeI-SP-HER2 ScFv-BamHI-myc-CD8 Stalk-CD28 TM-NruI-41BB-CD3z cyto-speI-EWS 28-262	This study (XSB1231)	Lentiviral vector
26	pHR NdeI-SP-CD19 ScFv-BamHI-myc-CD8 Stalk-CD8 TM-NruI-CD28-41BB-CD3z cyto-speI-EWS 28-262 Y to S-sGFP	This study (XSB1376)	Lentiviral vector
27	pHR NdeI-SP-CD19 ScFv-BamHI-myc-CD8 Stalk-CD8 TM-NruI-CD28-41BB-CD3z cyto-10xIDR 30Y speI-sGFP	This study (XSB1380)	Lentiviral vector

IDR sequence

1	**FUS**	MASND YTQQATQSYGAYPTQPGQGY SQQSSQPYGQQSYSGYSQST<br>DTSGYGQSSYSSYGQSQNTG YGTQSTPQGYGSTGGYGSSQ<br>SSQSSYGQQSSYPGYGQQPAPSSTSGSYGSSSQSSSYGQP<br>QSGSYSQQPSYGGQQQSYGQ QQSYNPPQGYGQQNQYNSSS<br>GGGGGGGGGGNYGQDQSSMS SGGGSGGGYGNQDQSGGGGS<br>GGYGQQDRG
2	**EWS**	GYAQTTQA YGQQSYGTYGQPTDVSYTQA QTTATYGQTAYATSYGQPPT<br>GYTTPTAPQAYSQPVQGYGT GAYDTTTATVTTTQASYAAQ<br>SAYGTQPAYPAYGQQPAATA PTRPQDGNKPTETSQPQSST<br>GGYNQPSLGYGQSNYSYPQV PGSYPMQPVTAPPSYPPTSY<br>SSTQPTSYDQSSYSQQNTYG QPSSYGQQSSYGQQSSYGQQ<br>PPTSYPPQTGSYSQAPSQYSQQSSSYG
3	**TAF15**	MSDSGSYG QSGGEQQSYSTYGNPGSQGY<br>GQASQSYSGYGQTTDSSYGQNYSGYSSYGQSYSQSYGGYE<br>NQKQSSYSQQPYNNQGQQQN MESSGSQGGRAPSYDQPDYG<br>QQDSYDQQSGYDQHQGSYDE QSNYDQQHDSYSQNQQSYHS<br>QRENYSHHTQDDRRDVSRYG EDNRGYGGSQGG
4	**NUP98**	FNKSFGTPFG GGTGGFGTTSTFGQNTGFGT TSGGAFGTSAFGSSNNTGGL<br>FGNSQTKPGGLFGTSSFSQP ATSTSTGFGFGTSTGTANTL<br>FGTASTGTSLFSSQNNAFAQ<br>NKPTGFGNFGTSTSSGGLFG TTNTTSNPFGSTSGSLFGPS<br>SFTAAPTGTTIKFNPPTGTD TMVKAGVSTNISTKHQCITA<br>MKEYESKSLEELRLEDYQAN RKGPQNQVGAGTTTGLFGSS<br>PATSSATGLFSSSTTNSGFA YGQNKTAFGTSTTGFGTNPG<br>GLFGQQNQQTTSLFSKPFGQ ATTTQNTGFSFGNTSTIGQP<br>STNTMGLFGVTQASQPGGLF GTATNTSTGT
5	**TDP43**	SNRQLERSGR FGGNPGGFGNQGGFGNSRGG GAGLGNNQGSNMGGGMNFGA<br>FSINPAMMAAAQAALQSSWG MMGMLASQQNQSGPSGNNQN<br>QGNMQREPNQAFGSGNNSYS GSNSGAAIGWGSASNAGSGS<br>GFNGGFGSSMDSKSSGWGM
6	**SynIDR**	SKGPGRGDSPY SGRGDSPYSGRGDSPYSGRG DSPYSGRGDSPYSGRGDSPY<br>SGRGDSPYSGRGDSPYSGRG<br>DSPYSGRGDSPYSGY
7	**EWS28-262 (Y to S)**	GSAQTTQASGQQSSGTSGQP TDVSSTQAQTTATSGQTASA<br>TSSGQPPTGSTTPTAPQASS QPVQGSGTGASDTTTATVTT<br>TQASSAAQSASGTQPASPAS GQQPAATAPTRPQDGNKPTE<br>TSQPQSSTGGSNQPSLGSGQ SNSSSPQVPGSSPMQPVTAP<br>PSSPPTSSSSTQPTSSDQSS SSQQNTSGQPSSSGQQSSSG<br>QQSSSGQQPPTSSPPQTGSS SQAPSQSSQQSSSSG
8	**SynIDR 30Y**	YSKGYPGRYGDSYPYSYGRG YDSPYYSGYRGDYSPYYSGR<br>YGDSYPYSYGRGYDSPYYSG YRGDYSPYYSGRYGDSYPYS<br>YGRGYDSPYYSGYRGDYSPY YSGRYGDSYPYSYGY

Antibodies

1	PE anti-human CD19 Antibody(4G7)	BioLegend	#392506
2	PE anti-human CD22 Antibody(S-HCL-1)	BioLegend	#363503
3	PE anti-human CD340 (erbB2/HER-2) Antibody(24D2)	BioLegend	#324405
4	APC anti-human CD19 Antibody(4G7)	BioLegend	#392504
5	APC anti-human CD22 Antibody(S-HCL-1)	BioLegend	#363506
6	Alexa Fluor® 647 anti-human CD340 (erbB2/HER-2) Antibody(24D2)	BioLegend	#324412
7	APC anti-human CD69 Antibody (FN50)	BioLegend	#310910
8	Brilliant Violet 421™ anti-human CD69 Antibody (FN50)	BioLegend	#310930
9	APC anti-human CD366 (TIM3) Monoclonal Antibody (F38-2E2)	Invitrogen	#17-3109-42
10	PE anti-human CD279 (PD-1) Monoclonal Antibody (MIH4)	Invitrogen	#12-9969-42
11	PE-Cyanine7 anti-human CD223 (LAG-3) Monoclonal Antibody (3DS223H)	Invitrogen	#25-2239-42
12	Pacific Blue™ Mouse Anti-Human CD3 (UCHT1)	BioLegend	#300417
13	PerCP/Cyanine5.5 anti-human CD45RA Antibody (HI100)	BioLegend	#304122
14	Pacific Blue™ anti-human CD4 Antibody (OKT4)	BioLegend	#317424
15	BD Horizon™ BUV496 Mouse Anti-Human CD4(SK3)	BDbiosciences	#612936
16	Brilliant Violet 785™ anti-human CD8 Antibody (SK1)	BioLegend	#344739
17	PE/Cy7 anti-human CD8a (RPA-T8)	BioLegend	#301012
18	APC/Fire™ 750 anti-human CD62L Antibody (DREG-56)	BioLegend	#304845
19	Brilliant Violet 750™ anti-human CD45RA Antibody (HI100)	BioLegend	#304166
20	BD Phosflow™ Alexa Fluor® 647 Mouse anti-CD247 (pY142) (K25-407.69)	BDbiosciences	#558489
21	Alexa Fluor® 647 anti-LAT Phospho (Tyr171) Antibody (A20005D)	BioLegend	#946603
22	Myc-Tag Mouse mAb (Alexa Fluor® 647 Conjugate) (9B11)	Cell Signaling Technology	#2233
23	APC/Fire™ 750 anti-human/mouse Granzyme B Recombinant Antibody (QA18A28)	BioLegend	#396417
24	PerCP anti-human CD45 Antibody (HI30)	BioLegend	#304026
25	APC anti-human CD45 Antibody (HI30)	BioLegend	#304012
26	Monoclonal Anti-FMC63 Antibody, Mouse IgG1 (Y45)	ACROBiosystems	#FM3-Y45
27	Phospho-Zap-70(Tyr319)/Syk (Tyr352) Antibody	Cell Signaling Technology	#2701
28	Phospho-LAT (Tyr191) antibody	Cell Signaling Technology	#3584s

29	Phospho-Lck (Tyr505) antibody	Cell Signaling Technology	#2751
30	Phospho-PLCγ1(Tyr783) antibody	Cell Signaling Technology	#2821
31	Phospho-BTK/ITK (Tyr551, Tyr511) antibody	eBioscience	#14-9015-82
32	Phospho-SLP-76 (Ser376) (E3G9U) antibody	Cell Signaling Technology	#76384
33	Phospho-p44/42 MAPK(Erk1/2) (Thr202/Tyr204) antibody	Cell Signaling Technology	#9101
34	Human Phospho-NFATC1 (S172) Antibody	R&D System	#MAB5640
35	Myc-Tag (9B11)	Cell Signaling Technology	#2276
36	Purified anti-GAPDH Antibody (FF26A/F9)	BioLegend	#649202

Others

1	EasySep™ Human T Cell Isolation Kit	Stem Cell	#17951
2	Human T-Activator CD3/CD28 Dynabeads	ThermoFisher	#11161D
3	Recomninant Interleukin-2	PEPROTECH	# 200-02
4	Human Recombinant Biotinylated Her2/ERBB2 protein	Sino Biological	#10004-H08H-B
5	Human Recombinant Biotinylated CD22 protein	Sino Biological	#11958-H41H-B
6	Recombinant Human ICAM-1/CD54 Protein (His Tag)	Sino Biological	# 10346-H08H
7	Streptavidin, Alexa Fluor™ 647 conjugate	Thermo Fisher Scientific	#S21374
8	Luciferase Assay System	Promega	# E1500
9	Cell lysis reagent	Promega	# 1531
10	Red Blood cell lysis buffer	BioLegend	#420301
11	D-Luciferin, Sodium Salt	Gold Biotechnology	#LUCNA-100
12	Fixation/Permeabilization Solution	BD Bioscience	# 554714
13	TNFα ELISA kit	BioLegend	# 430204
14	BD Quantibrite PE Phycoerythrin Fluorescence Quantitation Kit	BD Bioscience	#340495
15	LEGENDplex™ Human CD8/NK Panel (13-plex)	BioLegend	#741186
16	Chromium Next GEM Single Cell 3' Kit v3.1	10xGenomics	#PN-1000269
17	CellMask™ Plasma Membrane Stains	Invitrogen™	#10046

18	Indo-1, AM, cell permeant	Thermo Fisher Scientific	#I1223
19	Celltrace™ Far Red Cell Proliferation Kit	Thermo Fisher Scientific	# C34564
