# Supplementary material for: IDR-induced CAR condensation improves the cytotoxicity of CAR-Ts against low-antigen cancers
Source: Nat Chem Biol. 2025 Sep 29;22(3):379–91. doi: 10.1038/s41589-025-02031-x (PMC12825998; doi:10.1038/s41589-025-02031-x)
Supplement: Supplementary file 2 — Reporting Summary [file 41589_2025_2031_MOESM2_ESM.pdf]

nature portfolio

Corresponding author(s): Xiaolei Su

Last updated by author(s): Aug 20, 2025

Reporting Summary

Nature Portfolio wishes to improve the reproducibility of the work that we publish. This form provides structure for consistency and transparency in reporting. For further information on Nature Portfolio policies, see our Editorial Policies and the Editorial Policy Checklist.

Statistics

For all statistical analyses, confirm that the following items are present in the figure legend, table legend, main text, or Methods section.

n/a	Confirmed	
☐	☒	The exact sample size (*n*) for each experimental group/condition, given as a discrete number and unit of measurement
☐	☒	A statement on whether measurements were taken from distinct samples or whether the same sample was measured repeatedly
☐	☒	The statistical test(s) used AND whether they are one- or two-sided<br>*Only common tests should be described solely by name; describe more complex techniques in the Methods section.*
☒	☐	A description of all covariates tested
☒	☐	A description of any assumptions or corrections, such as tests of normality and adjustment for multiple comparisons
☒	☐	A full description of the statistical parameters including central tendency (e.g. means) or other basic estimates (e.g. regression coefficient) AND variation (e.g. standard deviation) or associated estimates of uncertainty (e.g. confidence intervals)
☒	☐	For null hypothesis testing, the test statistic (e.g. *F*, *t*, *r*) with confidence intervals, effect sizes, degrees of freedom and *P* value noted<br>*Give P values as exact values whenever suitable.*
☒	☐	For Bayesian analysis, information on the choice of priors and Markov chain Monte Carlo settings
☒	☐	For hierarchical and complex designs, identification of the appropriate level for tests and full reporting of outcomes
☒	☐	Estimates of effect sizes (e.g. Cohen's *d*, Pearson's *r*), indicating how they were calculated

Our web collection on statistics for biologists contains articles on many of the points above.

Software and code

Policy information about availability of computer code

Data collection	Single cell RNA-sequencing reads were processed using Cell Ranger software (10xGenomics,version 7.2.0) with standard parameters and aligned to human reference transcriptome(GRCh38), which was done by Yale Center for Genome Analysis (YCGA).
Data analysis	Gene expression comparison was performed using Loupe Browser (10xGenomics version 7.0.0) and cluster annotation was performed manually based on marker gene expression. The comparison between two groups was done with GraphPad Prism9.

For manuscripts utilizing custom algorithms or software that are central to the research but not yet described in published literature, software must be made available to editors and reviewers. We strongly encourage code deposition in a community repository (e.g. GitHub). See the Nature Portfolio guidelines for submitting code & software for further information.

Data

Policy information about availability of data

All manuscripts must include a data availability statement. This statement should provide the following information, where applicable:

- Accession codes, unique identifiers, or web links for publicly available datasets
- A description of any restrictions on data availability
- For clinical datasets or third party data, please ensure that the statement adheres to our policy

The data supporting the findings of this study are available in the paper and its Supplementary Information files. The data of single-cell sequencing can be anonymously accessed at NCBI GEO GSE272224. Raw flow cytometry data, raw image data are available through the Yale Dataverse59-62. The detailed raw source

data for each Figure and Extended Data Figure is as following: Source data for Figure 1, 2,4,5,6 are available in the Yale Dataverse repository at https://doi.org/10.60600/YU/8UUAHW. Source data for Figure 3 are available in the Yale Dataverse repository at https://doi.org/10.60600/YU/REY5VG. Source data for Extended Data Figure 1,2,3,5,6,8,9,10 are available in the Yale Dataverse repository at https://doi.org/10.60600/YU/H2SZ02. Source data for Extended Data Figure 4 are available in the Yale Dataverse repository at https://doi.org/10.60600/YU/E6ZDOY.

Research involving human participants, their data, or biological material

Policy information about studies with human participants or human data. See also policy information about sex, gender (identity/presentation), and sexual orientation and race, ethnicity and racism.

Reporting on sex and gender	NA
Reporting on race, ethnicity, or other socially relevant groupings	NA
Population characteristics	NA
Recruitment	NA
Ethics oversight	NA

Note that full information on the approval of the study protocol must also be provided in the manuscript.

Field-specific reporting

Please select the one below that is the best fit for your research. If you are not sure, read the appropriate sections before making your selection.

☒ Life sciences ☐ Behavioural & social sciences ☐ Ecological, evolutionary & environmental sciences

For a reference copy of the document with all sections, see nature.com/documents/nr-reporting-summary-flat.pdf

Life sciences study design

All studies must disclose on these points even when the disclosure is negative.

Sample size	Sample sizes for both in vitro and in vivo experiments were selected based on standard practices in the field. For each experiment, we ensured that the number of technical and biological replicates, as well as different experiment methods for validation was sufficient to observe consistent and statistically meaningful differences between groups, as assessed by (e.g., Student's t-test, ANOVA). Additionally, results were reproducible across independent experiments. Given the observed effect sizes and low variability within groups, we believe the chosen sample sizes are sufficient to support the conclusions of the study.
Data exclusions	In image analysis, cells with extremely high or low intensities were excluded because of potential technical issue including cell death. All the other experiment no data was excluded.
Replication	Biological or technical replicates were showed in the figures and described in the corresponding figure legend.
Randomization	Mice were randomized when receiving CAR-T cell treatment.
Blinding	NA

Reporting for specific materials, systems and methods

We require information from authors about some types of materials, experimental systems and methods used in many studies. Here, indicate whether each material, system or method listed is relevant to your study. If you are not sure if a list item applies to your research, read the appropriate section before selecting a response.

Materials & experimental systems

n/a	Involved in the study	
☐	☒	Antibodies
☐	☒	Eukaryotic cell lines
☒	☐	Palaeontology and archaeology
☐	☒	Animals and other organisms
☒	☐	Clinical data
☒	☐	Dual use research of concern
☒	☐	Plants

Methods

n/a	Involved in the study	
☒	☐	ChIP-seq
☐	☒	Flow cytometry
☒	☐	MRI-based neuroimaging

Antibodies

Antibodies used

1 PE anti-human CD19 Antibody(4G7) BioLegend #392506
2 PE anti-human CD22 Antibody(S-HCL-1) BioLegend #363503
3 PE anti-human CD340 (erbB2/HER-2) Antibody(24D2) BioLegend #324405
4 APC anti-human CD19 Antibody(4G7) BioLegend #392504
5 APC anti-human CD22 Antibody(S-HCL-1) BioLegend #363506
6 Alexa Fluor® 647 anti-human CD340 (erbB2/HER-2) Antibody(24D2) BioLegend #324412
7 APC anti-human CD69 Antibody (FN50) BioLegend #310910
8 Brilliant Violet 421™ anti-human CD69 Antibody (FN50) BioLegend #310930
9 APC anti-human CD366 (TIM3) Monoclonal Antibody (F38-2E2) Invitrogen #17-3109-42
10 PE anti-human CD279 (PD-1) Monoclonal Antibody (MIH4) Invitrogen #12-9969-42
11 PE-Cyanine7 anti-human CD223 (LAG-3) Monoclonal Antibody (3DS223H) Invitrogen #25-2239-42
12 Pacific Blue™ Mouse Anti-Human CD3 (UCHT1) BioLegend #300417
13 PerCP/Cyanine5.5 anti-human CD45RA Antibody (HI100) BioLegend #304122
14 Pacific Blue™ anti-human CD4 Antibody (OKT4) BioLegend #317424
15 BD Horizon™ BUV496 Mouse Anti-Human CD4(SK3) BDbiosciences #612936
16 Brilliant Violet 785™ anti-human CD8 Antibody (SK1) BioLegend #344739
17 PE/Cy7 anti-human CD8a (RPA-T8) BioLegend #301012
18 APC/Fire™ 750 anti-human CD62L Antibody (DREG-56) BioLegend #304845
19 Brilliant Violet 750™ anti-human CD45RA Antibody (HI100) BioLegend #304166
20 BD Phosflow™ Alexa Fluor® 647 Mouse anti-CD247 (pY142) (K25-407.69) BDbiosciences #558489
21 Alexa Fluor® 647 anti-LAT Phospho (Tyr171) Antibody (A20005D) BioLegend #946603
22 Myc-Tag Mouse mAb (Alexa Fluor® 647 Conjugate) (9B11) Cell Signaling Technology #2233
23 APC/Fire™ 750 anti-human/mouse Granzyme B Recombinant Antibody (QA18A28)
BioLegend #396417
24 PerCP anti-human CD45 Antibody (HI30) BioLegend #304026
25 APC anti-human CD45 Antibody (HI30) BioLegend #304012
26 Monoclonal Anti-FMC63 Antibody, Mouse IgG1 (Y45) ACROBiosystems #FM3-Y45
27 Phospho-Zap-70(Tyr319)/Syk (Tyr352)Antibody Cell Signaling Technology #2701
28 Phospho-LAT (Tyr191) antibody Cell Signaling Technology #3584s
29 Phospho-Lck (Tyr505) antibody Cell Signaling Technology #2751
30 Phospho-PLCγ (Tyr783) antibody Cell Signaling Technology #2821
31 Phospho-BTK/ITK(Tyr551, Tyr511) antibody eBioscience #14-9015-82
32 Phospho-SLP-76 (Ser376)(E3G9U) antibody Cell Signaling Technology #76384
33 Phospho-p44/42 MAPK(Erk1/2)(Thr202/Tyr204) antibody Cell Signaling Technology #9101
34 Human Phospho-NFATC1 (S172) Antibody R&D System #MAB5640
35 Myc-Tag (9B11) Cell Signaling Technology #2276
36 Purified anti-GAPDH Antibody (FF26A/F9) BioLegend #649202

Validation

All primary antibodies used in this study were validated by the manufacturer for the relevant applications (e.g., flow cytometry, western blotting, immunofluorescence). Where applicable, antibody specificity was further confirmed either by detection of a band or by detection of the fluorescence intensity at the expected rangeat the expected molecular weight, use of knockout or isotype controls, or consistency with published studies. Detailed information, including antibody source, catalog numbers, and validation references, is provided

Eukaryotic cell lines

Policy information about cell lines and Sex and Gender in Research

Cell line source(s)

1 HEK293T Human embryonic kidney For lentivirus production
2 Raji B B-lymphocytes of an 11-year-old Nigerian boy with Burkitt's lymphoma. Raji B-ffLuc2-mCherry UCSF CCF For testing CD22 CAR
3 Raji B-CD19 high-ffLuc2-mCherry For testing CD19 CAR
4 Raji B-CD19 low-ffLuc2-mCherry For testing CD19 CAR
5 Nalm6-CD19 high-ffLuc2-GFP The peripheral blood of a 19-year-old male patient with acute lymphoblastic leukemia (ALL) in relapse For testing CD19 CAR
6 Nalm6-CD19 Low-ffLuc2-GFP For testing CD19 CAR
7 Nalm6-ffLuc2-GFP For testing CD22 CAR

8 Nalm6-CD22 high-ffLuc2-mCherry For testing CD22 CAR
9 Nalm6-CD22 low-ffLuc2-mCherry For testing CD22 CAR
10 K562 a bone marrow sample taken from a 53-year-old female patient with chronic myelogenous leukemia (CML) in blast crisis
11 K562-HER2 high-ffLuc2-mCherry For testing HER2 CAR
12 K562-HER2 low-ffLuc2-mCherry For testing HER2 CAR
13 SKOV3 The ascites (fluid buildup) of a 64-year-old Caucasian female patient with ovarian adenocarcinoma
14 SKOV3-ffLuc2-mCherry For testing HER2 CAR
15 HT29 A primary tumor taken from a 44-year-old Caucasian female patient with colorectal adenocarcinoma
16 HT29-ffLuc2-mCherry For testing HER2 CAR

Authentication	We will perform the STR profiling on parental cell lines and get the most recent authentication by matching profiles to reference stocks.
Mycoplasma contamination	All cell lines are tested mycoplasma negative.
Commonly misidentified lines (See ICLAC register)	We verified all cell lines against the ICLAC Register of Misidentified Cell Lines (v13, released 26 April 2024). None of the cell lines used in this study—including HEK293T, Raji B, Nalm6 variants, K562, SKOV3, HT29, nor any of their engineered ffLuc or CAR expressing derivatives—appear on the ICLAC list of known misidentified or cross contaminated lines.

Animals and other research organisms

Policy information about studies involving animals; ARRIVE guidelines recommended for reporting animal research, and Sex and Gender in Research

Laboratory animals	NSG mice (NOD.Cg-Prkdc^scid Il2rg^tm1Wjl/SzJ; 6 weeks old; female; Mus musculus) were purchased from The Jackson Laboratory (Stock No. 005557) and housed under specific pathogen–free conditions at Yale Animal Resources Center (YARC).
Wild animals	The study did not involve wild animals.
Reporting on sex	All the mice used in this study are female.
Field-collected samples	This study did not involve sample collected from the field.
Ethics oversight	The animal protocol has been approved by Institutional Animal Care & Use Committee (IACUC) of Yale.

Note that full information on the approval of the study protocol must also be provided in the manuscript.

Plants

Seed stocks	NA
Novel plant genotypes	NA
Authentication	NA

Flow Cytometry

Plots

Confirm that:

☒ The axis labels state the marker and fluorochrome used (e.g. CD4-FITC).

☒ The axis scales are clearly visible. Include numbers along axes only for bottom left plot of group (a 'group' is an analysis of identical markers).

☒ All plots are contour plots with outliers or pseudocolor plots.

☒ A numerical value for number of cells or percentage (with statistics) is provided.

Methodology

Sample preparation	To determine the cell-surface expression, cells were collected and blocked with an anti-human Fc receptor binding inhibitory antibody in the staining buffer (PBS with 2% FBS and 1 mM EDTA) for 15 minutes at 4°C, which were further incubated with individual antibodies in the staining buffer for 30 minutes on ice. The stained cells were washed twice with the staining buffer

before sending for flow cytometry analysis. To determine the intracellular expression of targets of interest, cells were collected and fixed with Fixation/Permeabilization Solution (Cat# 554714) for 15 min on ice, blocked in the staining buffer for 30 min on ice, followed by antibody staining. To characterize T cells in the mice blood, blood was drawn from a tail cut and diluted into PBS supplemented with 3 mM EDTA. Red blood cells were lysed with a red blood cell lysis buffer. The rest of cells were stained as described above and followed by flow cytometry. To characterize tumor-infiltrating T cells, tumors were dissected and digested with RPMI medium containing 0.5 mg/mL Collagenase P and 1 ?g/mL DNase per 100 mg of tumor tissues for 30 min at 37°C on a shaker. The digested tumor tissues were further homogenized and passed through a 40 ?m strainer, followed by centrifugation to collect cell samples. Cells were further stained and analyzed by flow cytometry as described above.

Instrument	LSRII, BD Symphony, CytoFlex, and Bigfoot cell sorter from Yale Flow Cytometry Facility. Aurora from the current lab.
Software	FlowJo.
Cell population abundance	The abundance of the relevant cell populations within post-sort fractions is depends on various samples, the details have been showed in the figures and it was determined by specific antibody staining and negative control.
Gating strategy	The gating strategy starts from gating alive cells based on cell size, followed by single cell gating based on the major population in the plot of FSC-H vs FSC-A, the second time single cell gating based on the major population in the plot of SSC-H vs SSC-A.

☒ Tick this box to confirm that a figure exemplifying the gating strategy is provided in the Supplementary Information.
