# Supplementary material for: IDR-induced CAR condensation improves the cytotoxicity of CAR-Ts against low-antigen cancers
Source: Nat Chem Biol. 2025 Sep 29;22(3):379–91. doi: 10.1038/s41589-025-02031-x (PMC12825998; doi:10.1038/s41589-025-02031-x)

Uncropped IVIS Image for for Figure 2K d-1

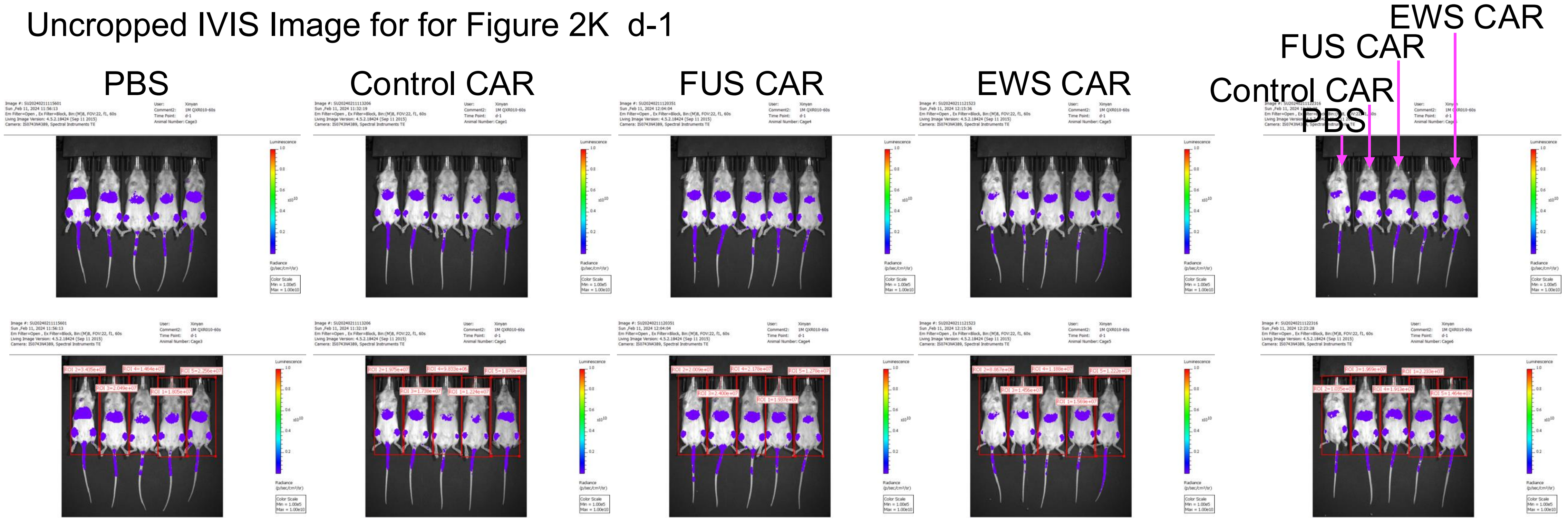

Uncropped IVIS Image for for Figure 2K d7

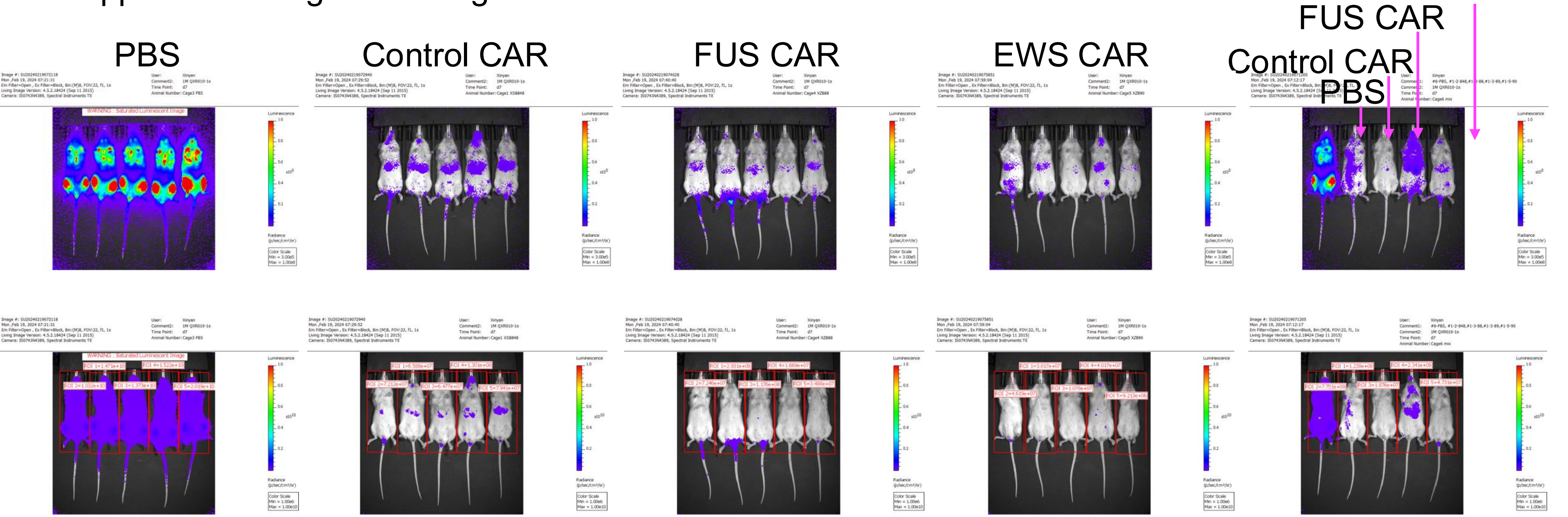

Uncropped IVIS Image for for Figure 2K d28

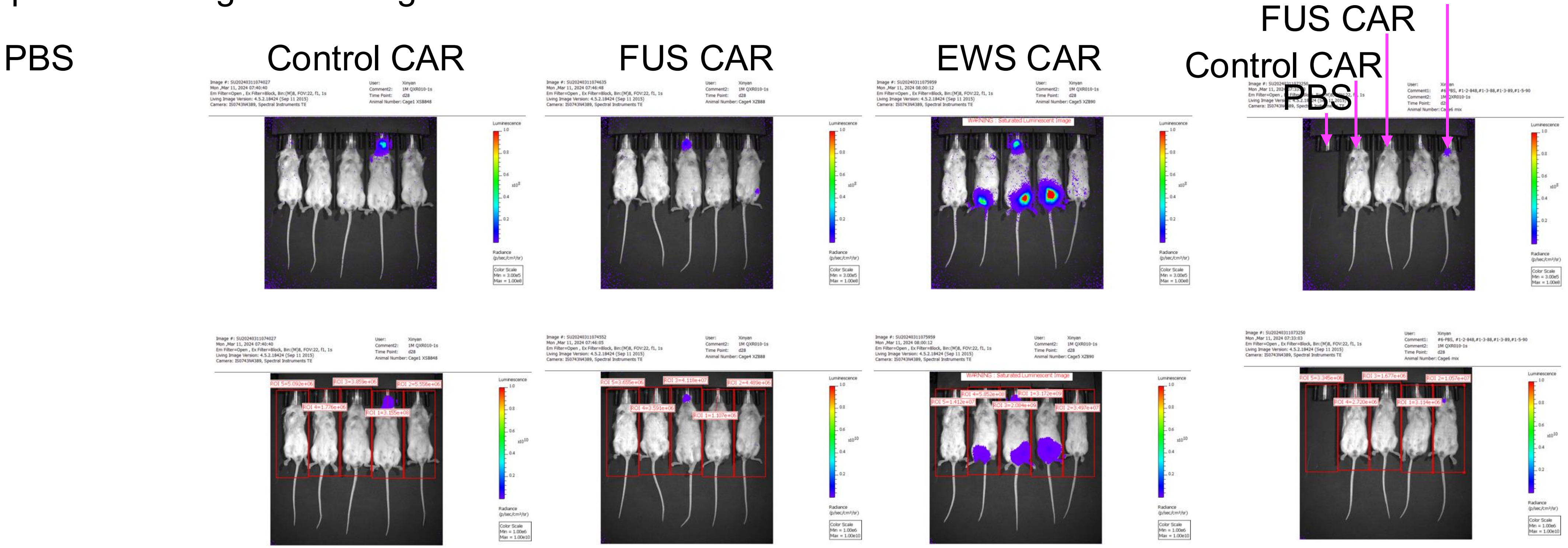

Uncropped IVIS Image for for Figure 2K d35

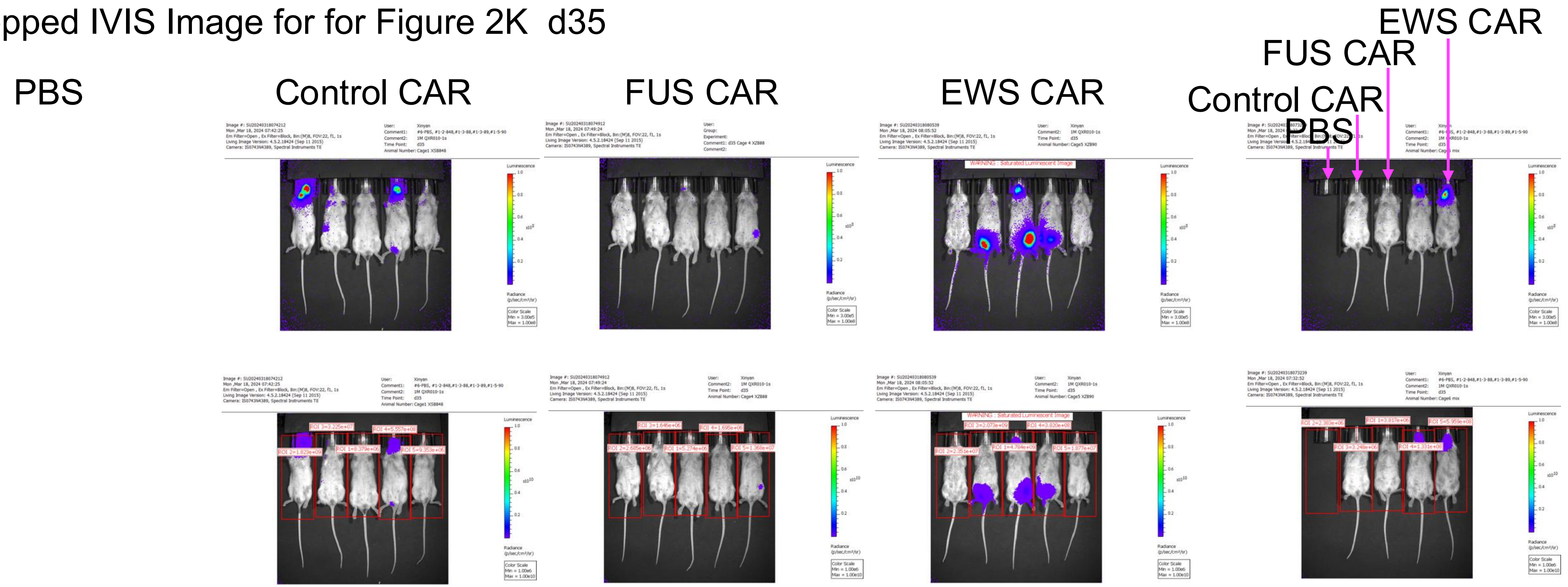

Uncropped IVIS Image for for Figure 2K d42

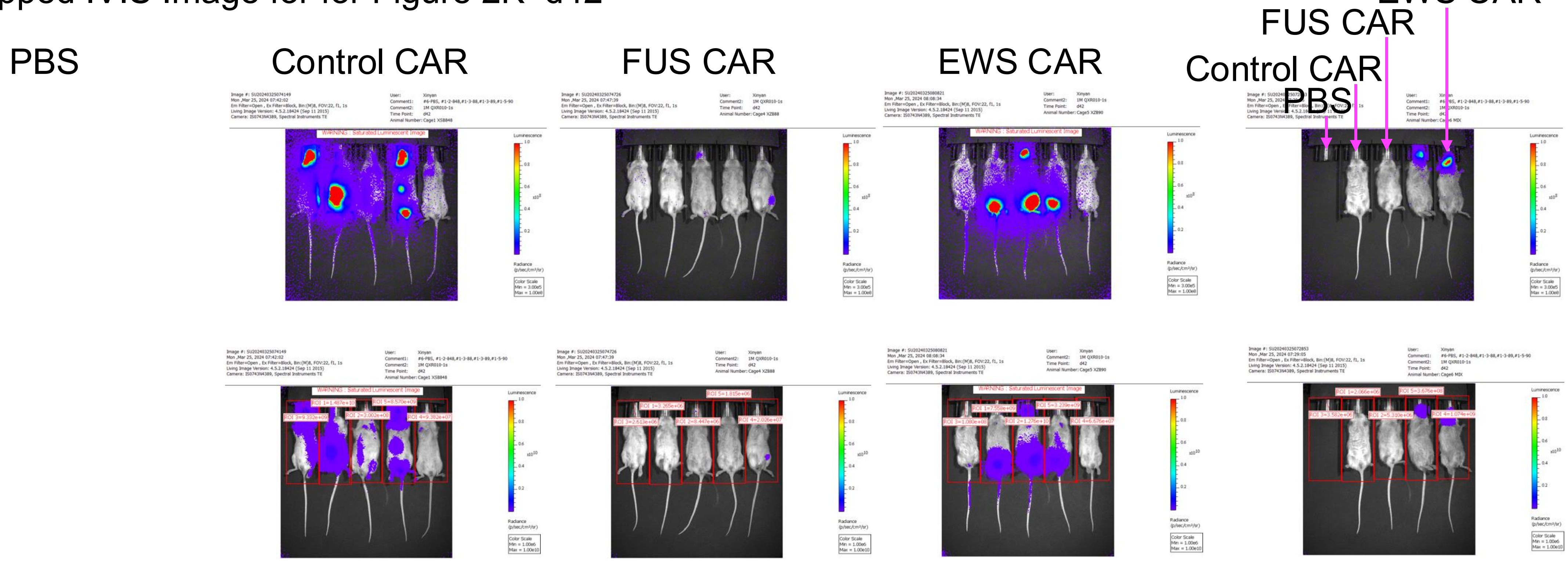

Uncropped IVIS Image for for Figure 4I   d-1

PBS | CD22 Control CAR | FUS CAR | EWS CAR

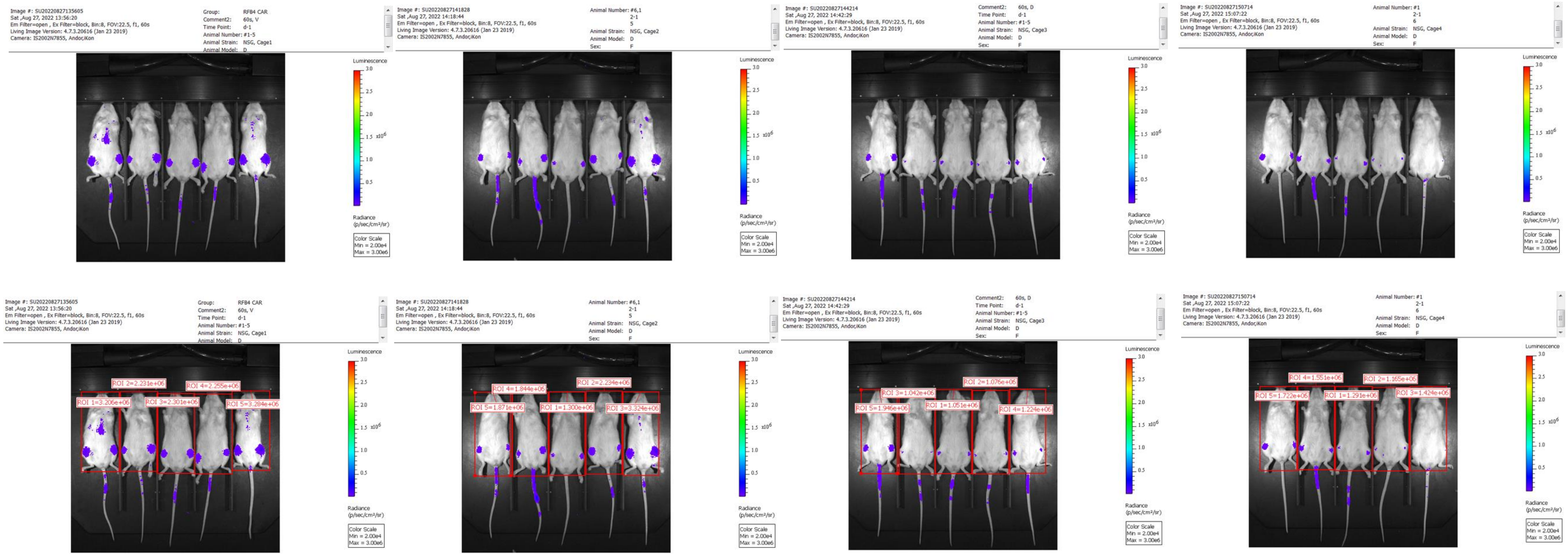

Uncropped IVIS Image for for Figure 4I   d5

PBS | CD22 Control CAR | FUS CAR | EWS CAR

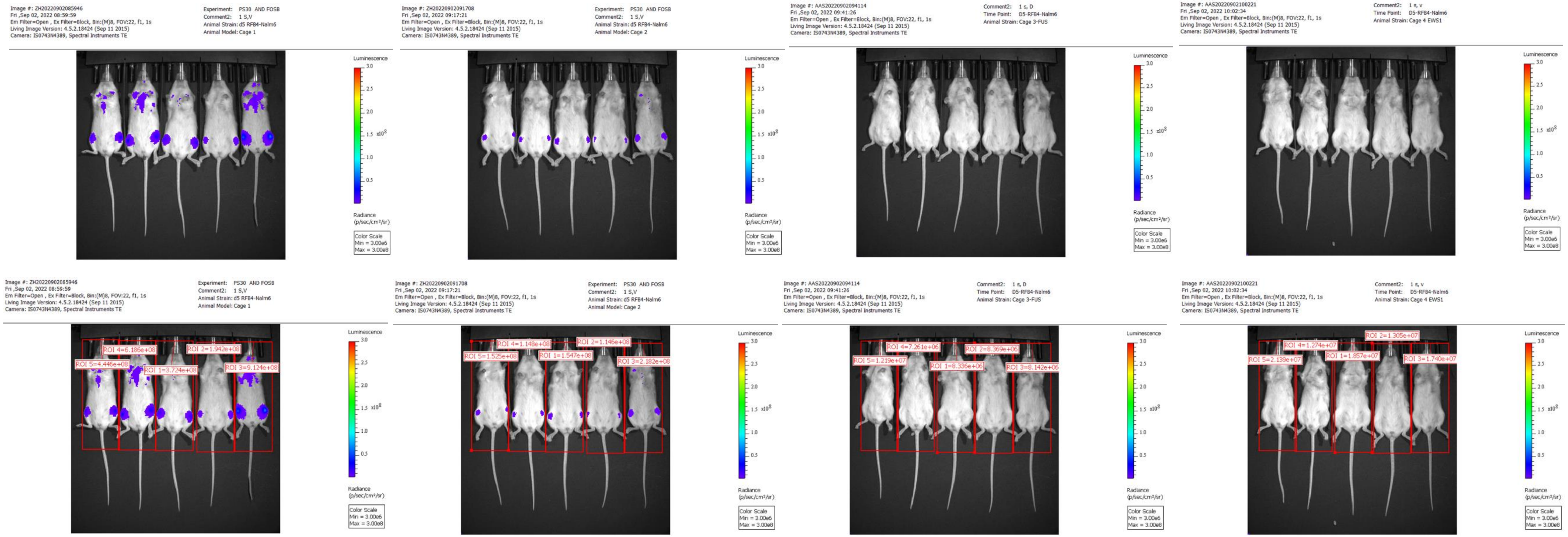

Uncropped IVIS Image for for Figure 4I d10

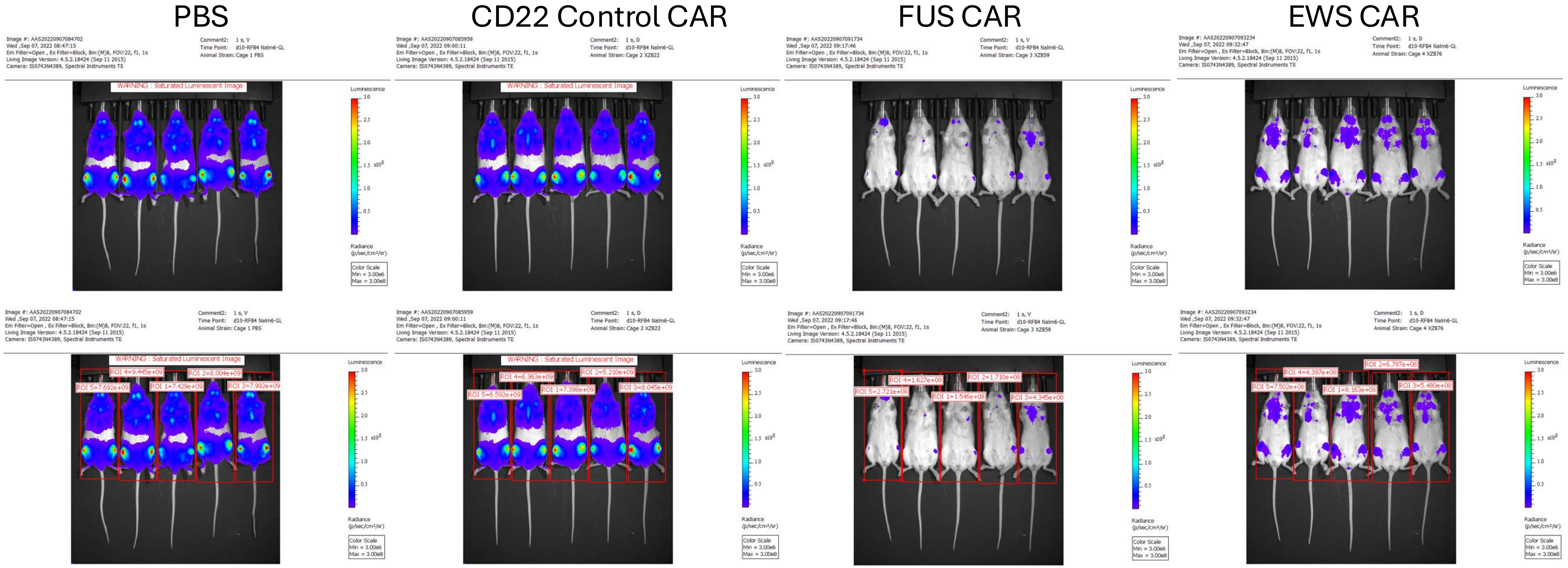

Uncropped IVIS Image for for Figure 4I d17

PBS | CD22 Control CAR | FUS CAR | EWS CAR

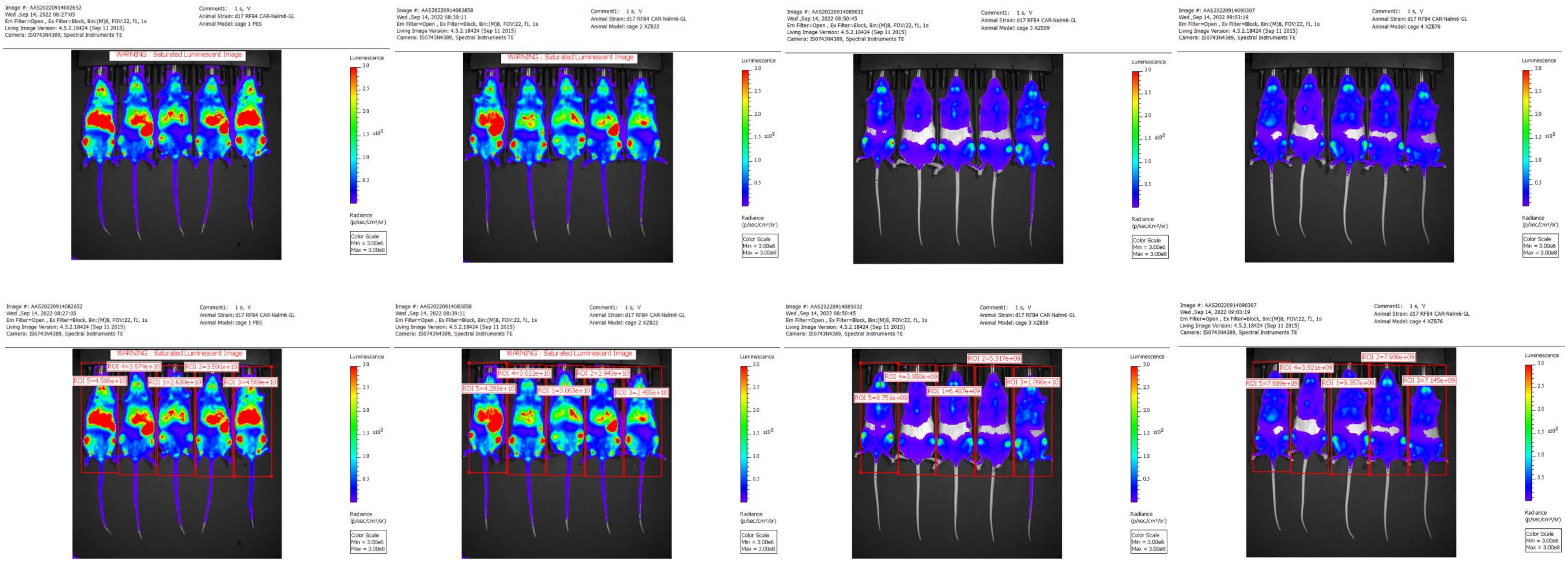

Uncropped IVIS Image for for Extended Data Figure 3b d-1

PBS | Control CAR | FUS CAR | EWS CAR

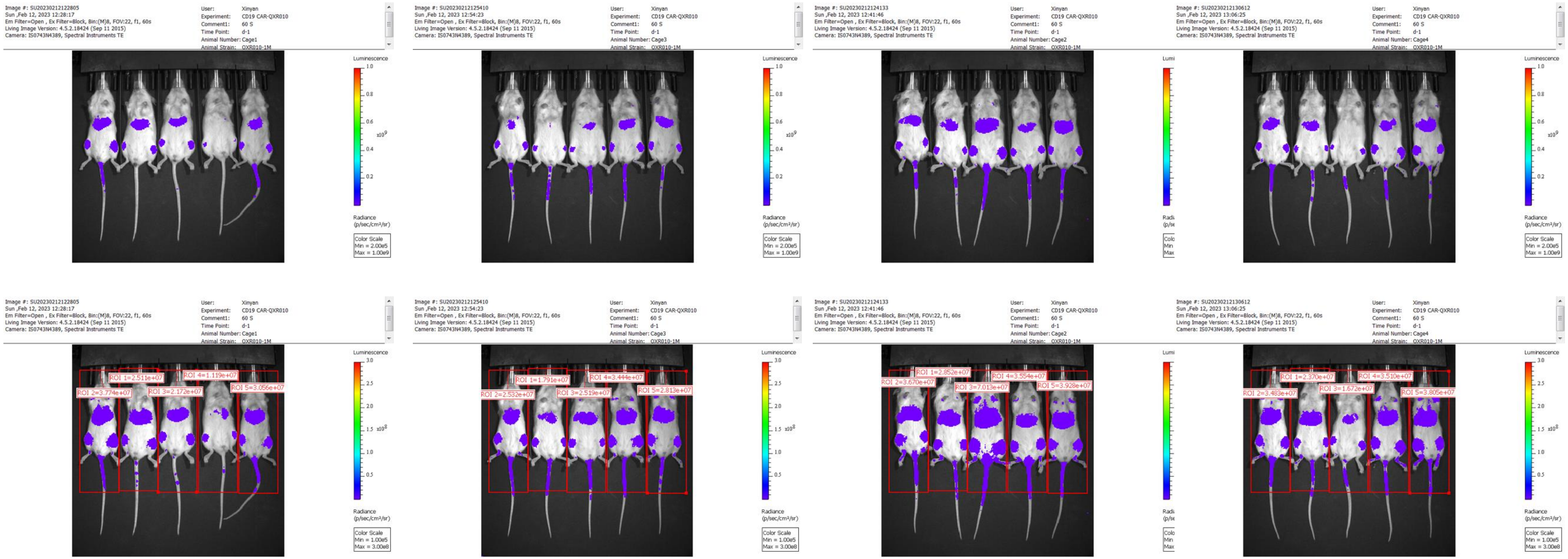

Uncropped IVIS Image for for Extended Data Figure 3b d8

PBS | Control CAR | FUS CAR | EWS CAR

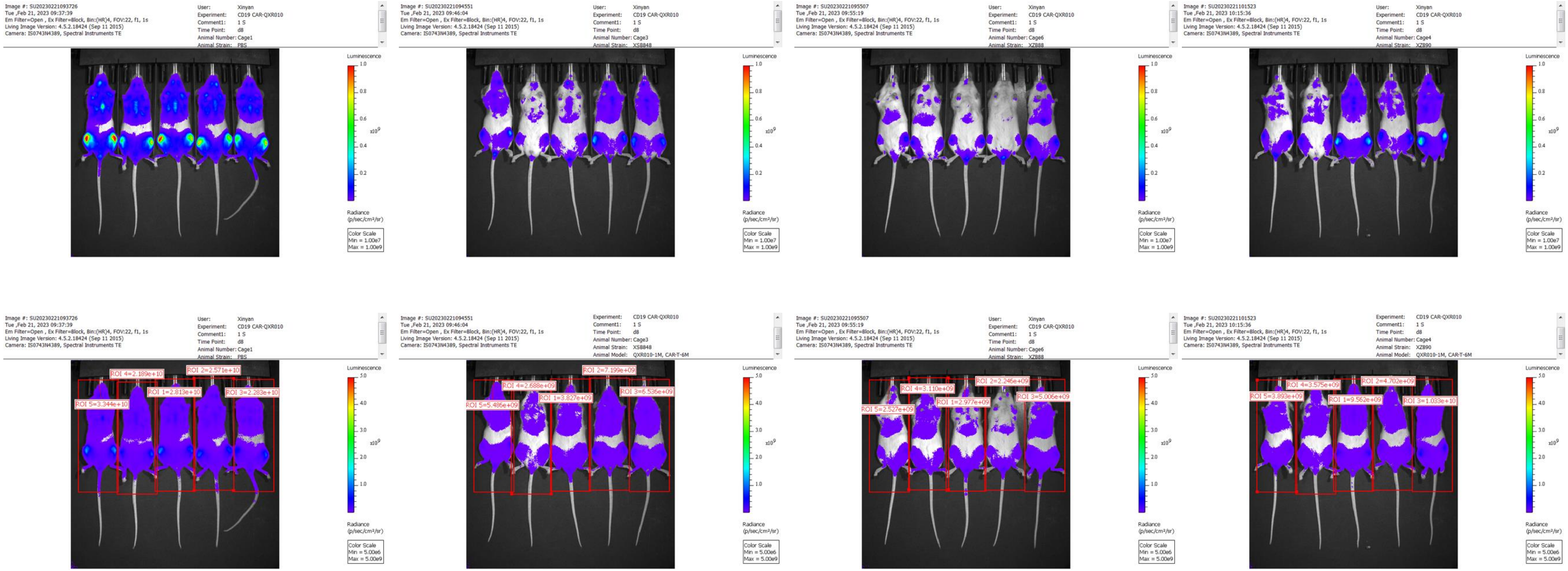

Uncropped IVIS Image for for Extended Data Figure 3b d12

PBS | Control CAR | FUS CAR | EWS CAR

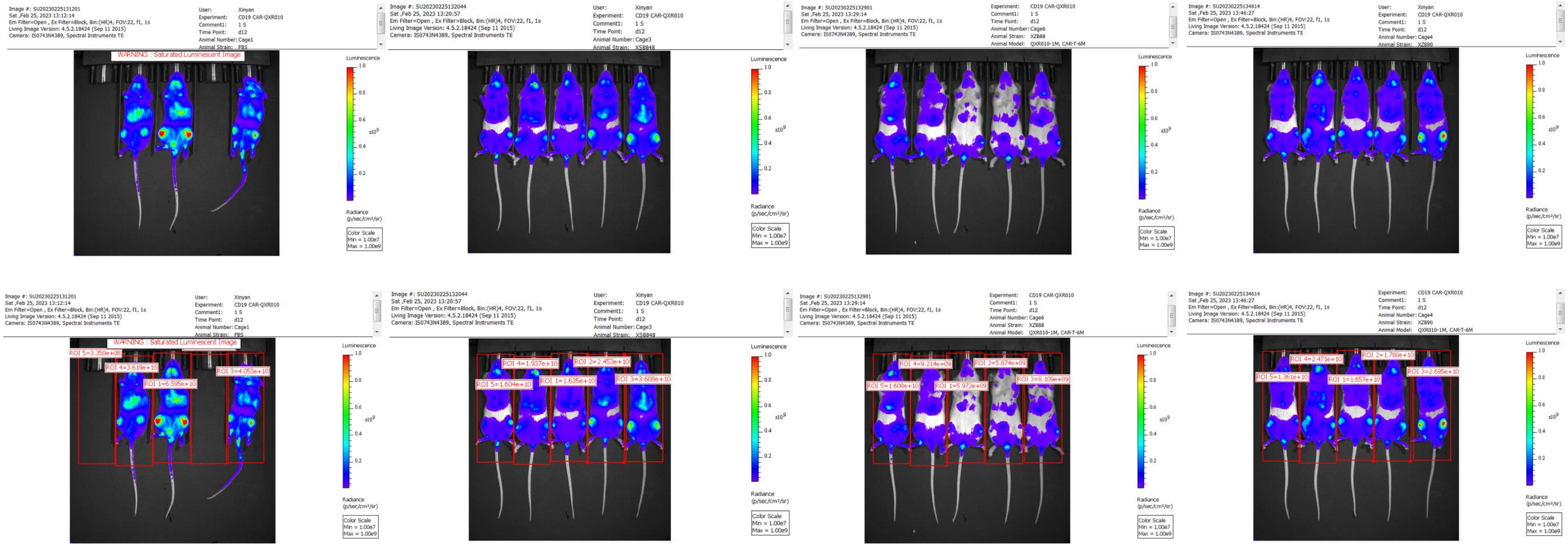

Uncropped IVIS Image for for Extended Data Figure 3b d16

PBS | Control CAR | FUS CAR | EWS CAR

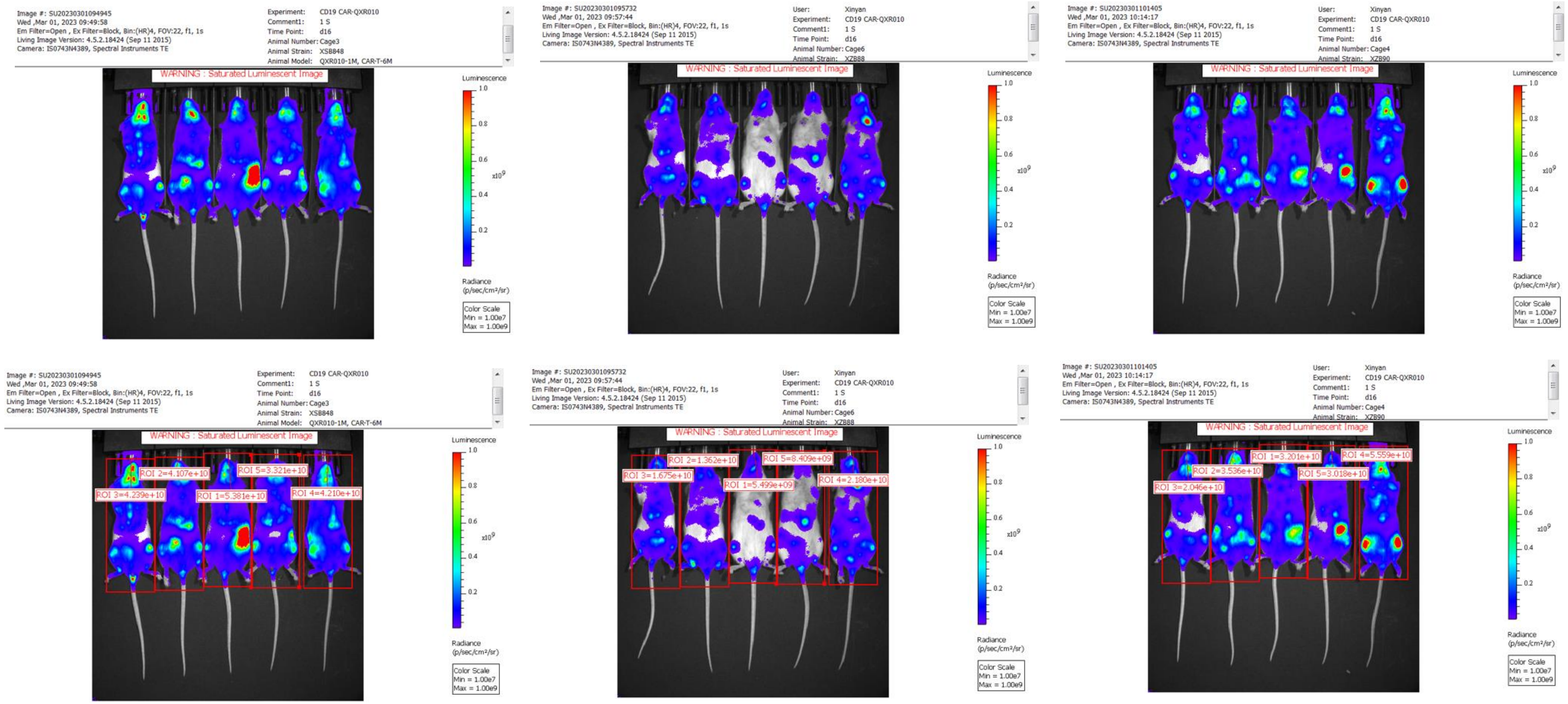

Uncropped IVIS Image for for Extended Data Figure 3b d35

PBS | Control CAR | FUS CAR | EWS CAR

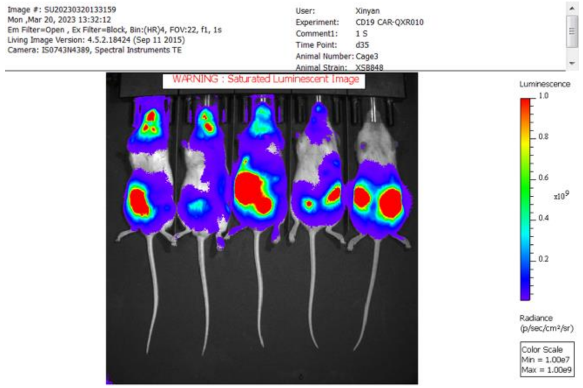

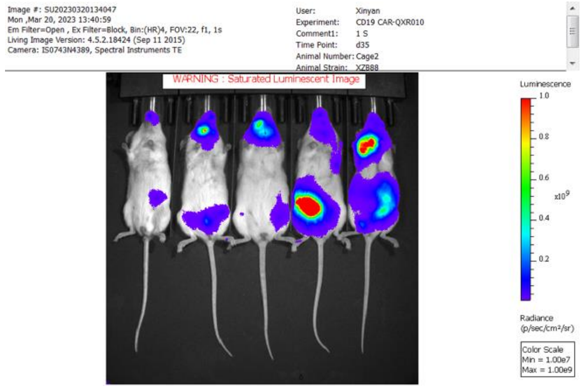

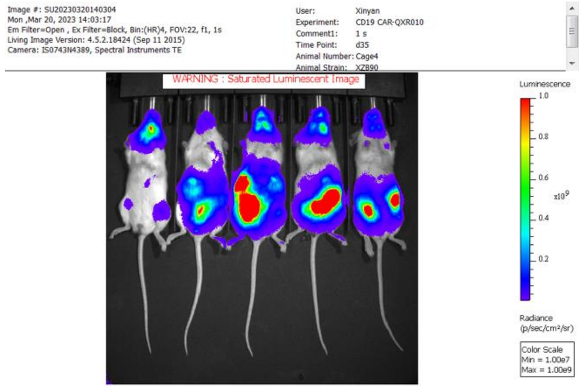

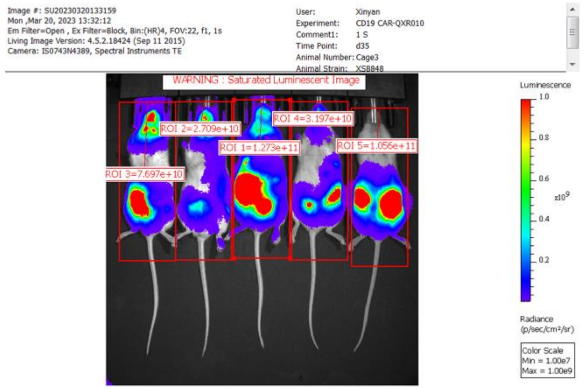

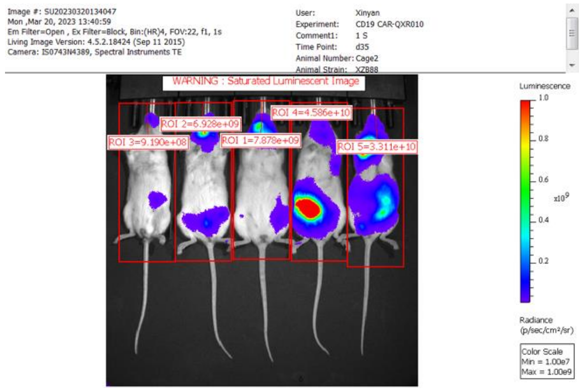

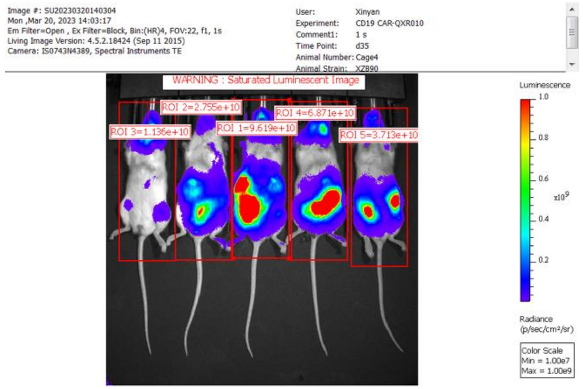

Uncropped IVIS Image for for Extended Data Figure 3b d58

PBS

Control CAR

FUS CAR

EWS CAR

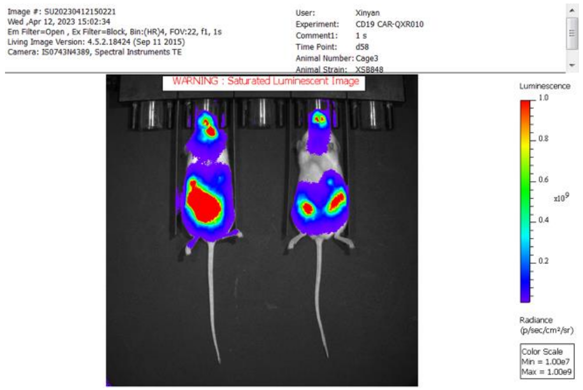

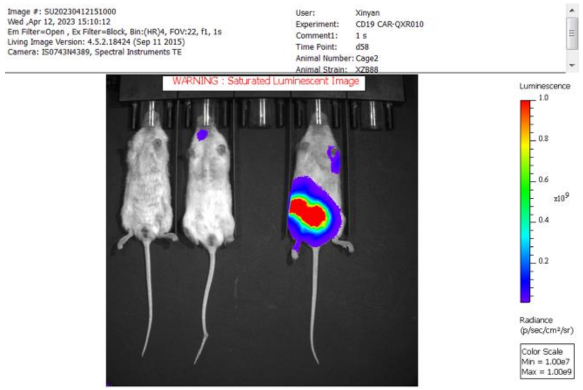

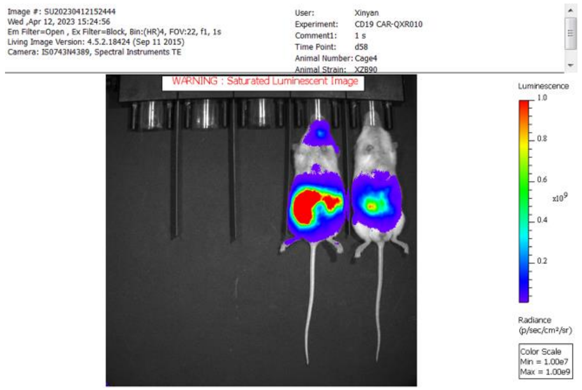

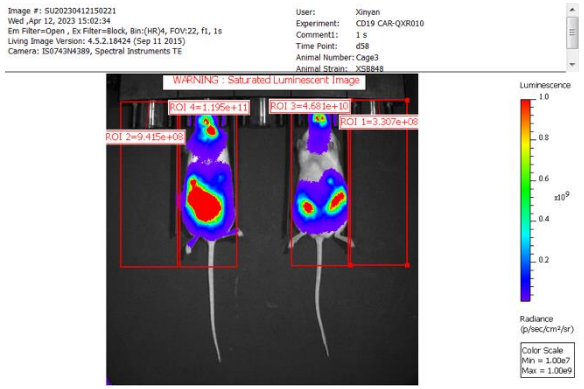

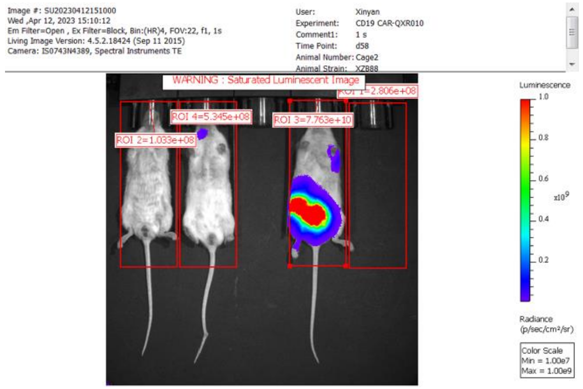

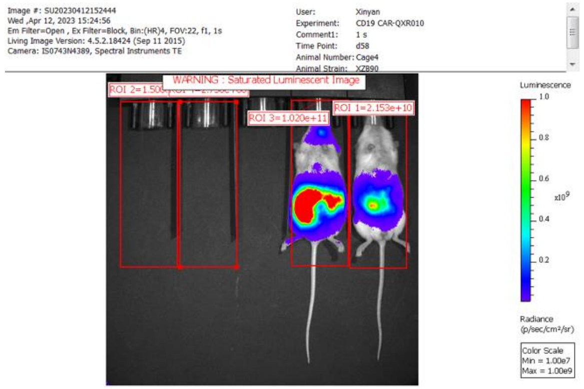

Supplement: Supplementary file 5 — Animal in vivo image source data. [file 41589_2025_2031_MOESM5_ESM.pdf]
