# Supplementary figures and images for: IDR-induced CAR condensation improves the cytotoxicity of CAR-Ts against low-antigen cancers
Source: Nat Chem Biol. 2025 Sep 29;22(3):379–91. doi: 10.1038/s41589-025-02031-x (PMC12825998; doi:10.1038/s41589-025-02031-x)

Uncropped WB image for Extended Data Figure 10a

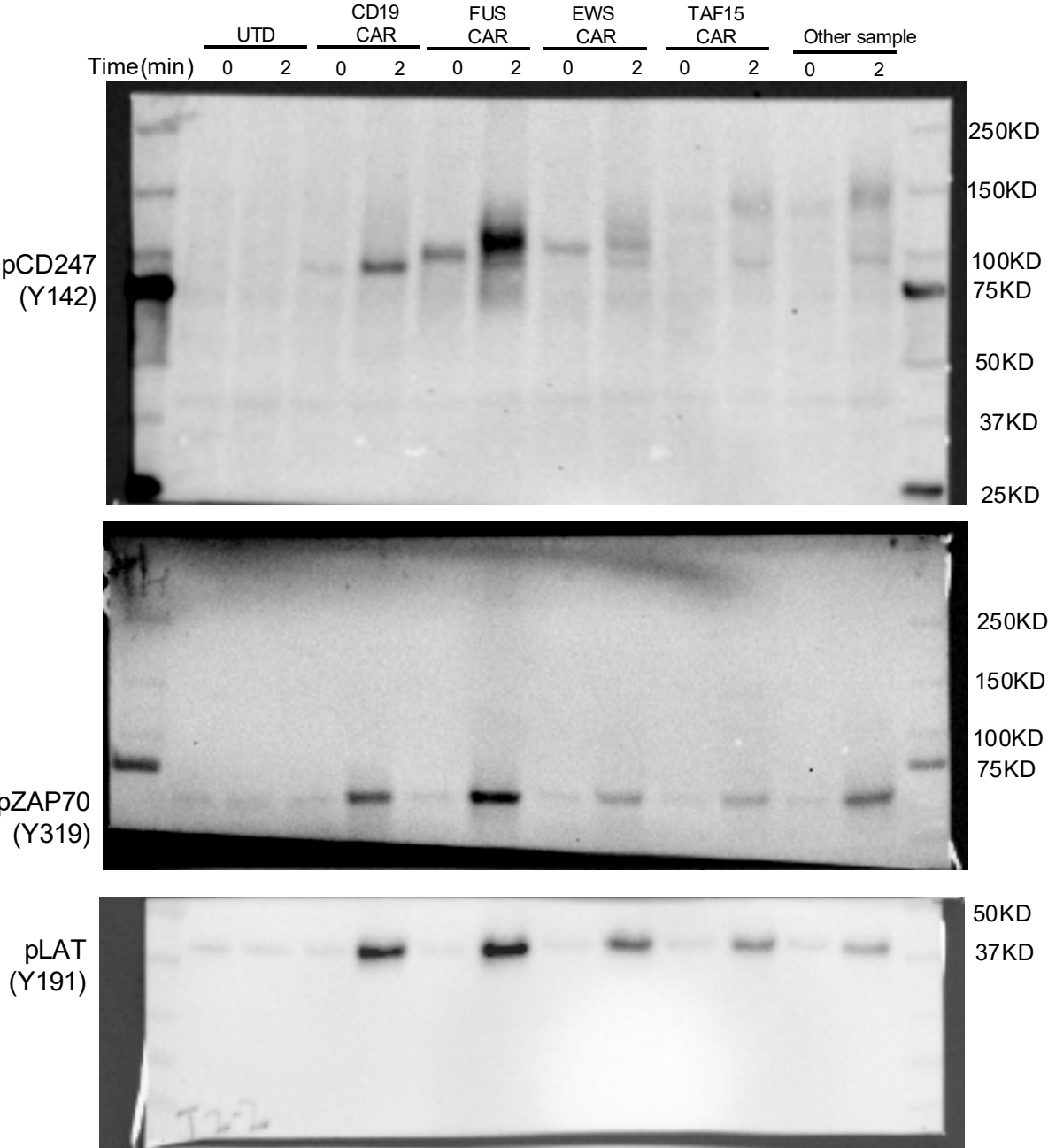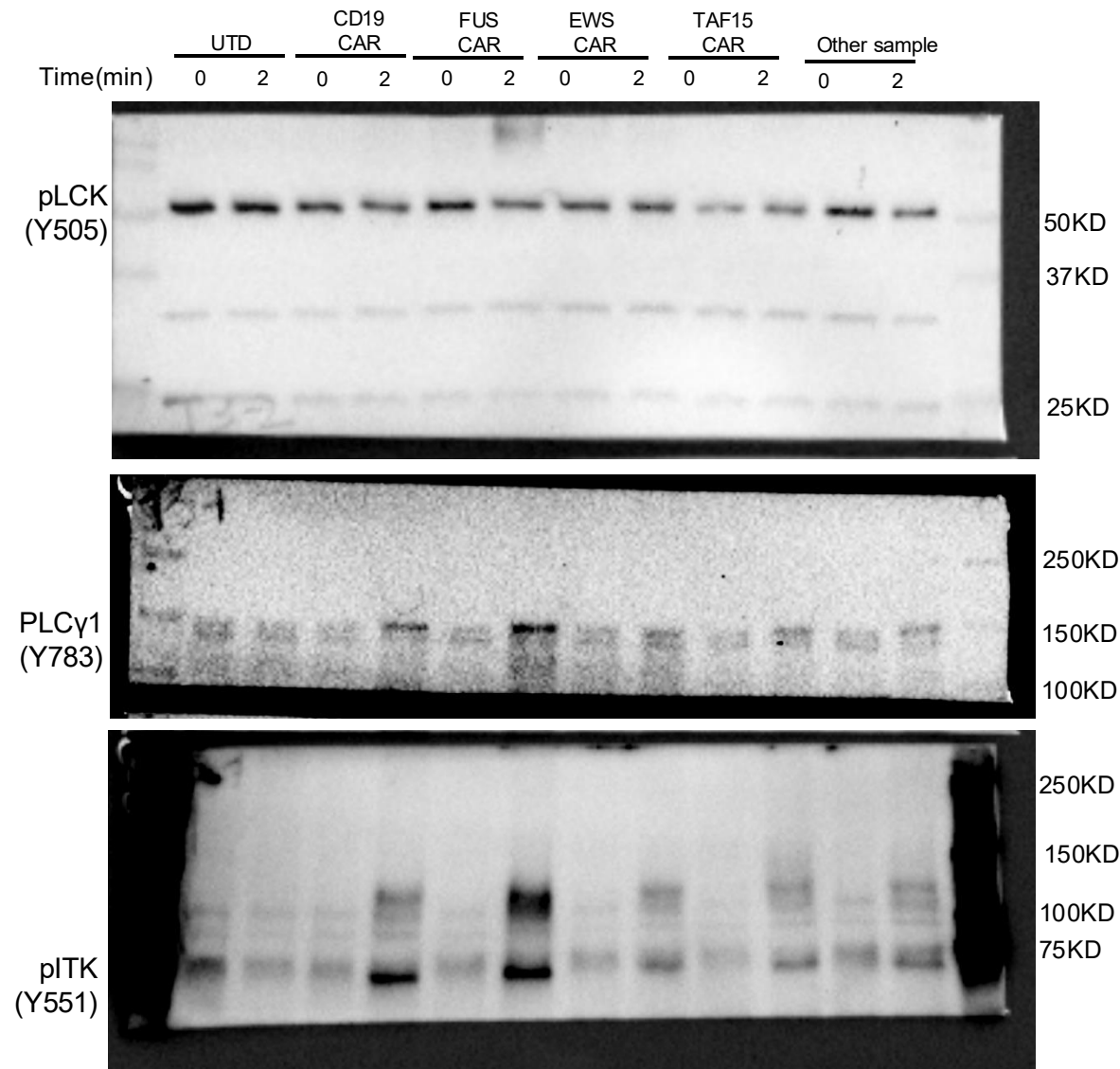

Uncropped WB image for Extended Data Figure 10a

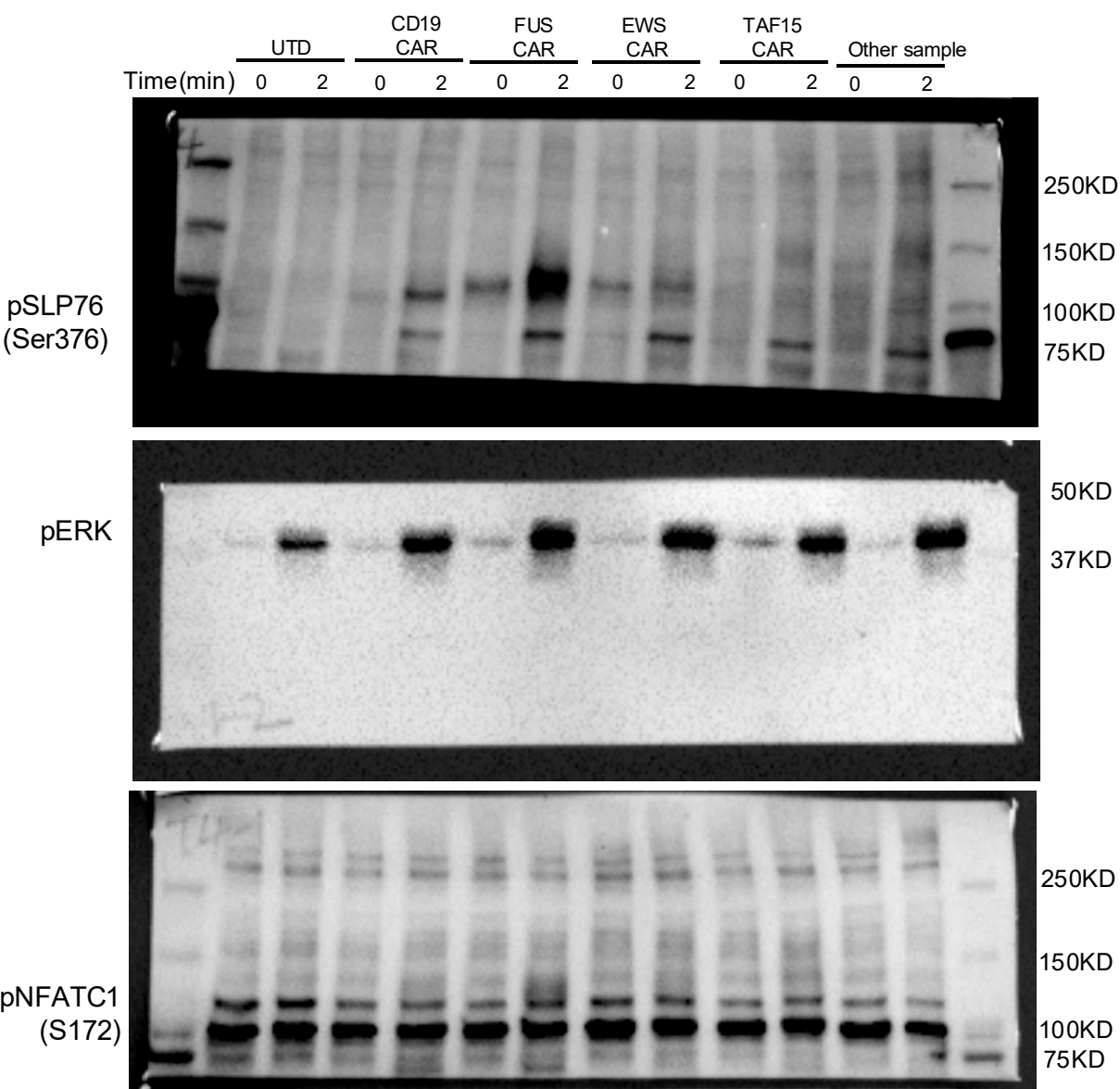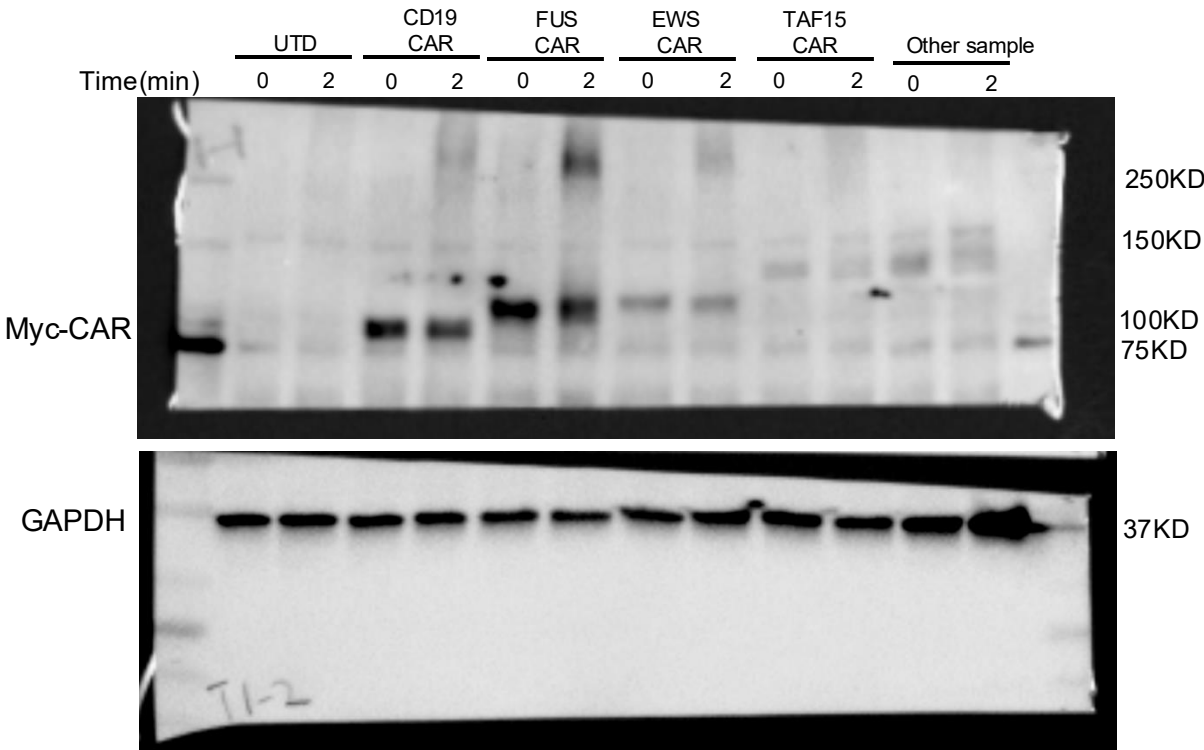

Supplement: Supplementary file 19 — Western blot uncropped image data. [file 41589_2025_2031_MOESM19_ESM.pdf]
