# Supplementary material for: IDR-induced CAR condensation improves the cytotoxicity of CAR-Ts against low-antigen cancers
Source: Nat Chem Biol. Author manuscript; Available in PMC 2026 Jan 23. (PMC12825998; doi:10.1038/s41589-025-02031-x)
Supplement: Supplementary file 1 [file NIHMS2123337-supplement-Table_S1.pdf]

Supplementary Table 1. Reagents

Cell lines

Cell lines	Source	Additional infoamation
HEK293T	UCSF CCF	For lentivirus production
Raji B-ffLuc2-mCherry	UCSF CCF	For testing CD22 CAR
Raji B-CD19 high-ffLuc2-mCherry	This study	For testing CD19 CAR
Raji B-CD19 low-ffLuc2-mCherry	This Study	For testing CD19 CAR
Nalm6-CD19 high-ffLuc2-GFP	Robbie Majzner Lab	For testing CD19 CAR
Nalm6-CD19 low-ffLuc2-GFP	Robbie Majzner Lab	For testing CD19 CAR
Nalm6-ffLuc2-GFP	Sidi Chen Lab	For testing CD22 CAR
Nalm6-CD22 high-ffLuc2-mCherry	This Study	For testing CD22 CAR
Nalm6-CD22 low-ffLuc2-mCherry	This Study	For testing CD22 CAR
K562	Jun Lu Lab	
K562-HER2 high-ffLuc2-mCherry	This study	For testing CD22 CAR
K562-HER2 low-ffLuc2-mCherry	This study	For testing CD22 CAR
SKOV3	Elena Ratner Lab	
SKOV3-ffLuc2-mCherry	This study	For testing CD22 CAR
HT29	Sidi Chen Lab	
HT29-ffLuc2-mCherry	This Study	For testing CD22 CAR

Plasmids

1	pMD2.G	Vale Lab	Viral packaging
2	poPAX	Vale Lab	Viral packaging
3	pHR-mCherry-CAAX	This study (XSB473)	Lentiviral vector
4	pFUGW-FerH-ffLuc2-mCherry	This study (XSB887)	Lentiviral vector
5	pFUGW-FerH-ffLuc2-GFP	This study (XSB886)	Lentiviral vector
6	pHR-CD19-mCherry	This study (XSB875)	Lentiviral vector
7	pHAGE-ERBB2 (HER2)	This study (XSB1100)	Lentiviral vector
8	pHR NdeI-SP-CD19 ScFv-BamHI-myc-CD8 Stalk-CD8 TM-NruI-CD28-41BB-CD3z cyto-speI-sGFP	This study (XSB848)	Lentiviral vector
9	pHR NdeI-SP-CD19 ScFv-BamHI-myc-CD8 Stalk-CD8 TM-NruI-CD28-41BB-CD3z cyto-speI-FUS 1-214-sGFP	This study (XSB1261)	Lentiviral vector
10	pHR NdeI-SP-CD19 ScFv-BamHI-myc-CD8 Stalk-CD8 TM-NruI-CD28-41BB-CD3z cyto-speI-TAF15 1-180-sGFP	This study (XSB1315)	Lentiviral vector
11	pHR NdeI-SP-CD19 ScFv-BamHI-myc-CD8 Stalk-CD8 TM-NruI-CD28-41BB-CD3z cyto-speI-EWS 28-262-sGFP	This study (XSB1316)	Lentiviral vector

12	pHR NdeI-SP-CD19 ScFv-BamHI-myc-CD8 Stalk-CD8 TM-NruI-CD28-41BB-CD3z cyto-speI-Nup98 1-340-sGFP	This study (XZB101)	Lentiviral vector
13	pHR NdeI-SP-CD19 ScFv-BamHI-myc-CD8 Stalk-CD8 TM-NruI-CD28-41BB-CD3z cyto-TDP43 266-414 speI-sGFP	This study (XSB929)	Lentiviral vector
14	pHR NdeI-SP-CD19 ScFv-BamHI-myc-CD8 Stalk-CD8 TM-NruI-CD28-41BB-CD3z cyto-10xIDR-speI-sGFP	This study (XSB930)	Lentiviral vector
15	pHR NdeI-SP-CD19 ScFv-BamHI-myc-CD8 Stalk-CD8 TM-NruI-CD28-41BB-CD3z cyto-speI-FUS 1-214	This study (XSB1227)	Lentiviral vector
16	pHR NdeI-SP-CD19 ScFv-BamHI-myc-CD8 Stalk-CD8 TM-NruI-CD28-41BB-CD3z cyto-speI-TAF15 1-180	This study (XSB1228)	Lentiviral vector
17	pHR NdeI-SP-CD19 ScFv-BamHI-myc-CD8 Stalk-CD8 TM-NruI-CD28-41BB-CD3z cyto-speI-EWS 28-262	This study (XSB1229)	Lentiviral vector
18	pHR NdeI-SP-CD22 RFB4 ScFv-BamHI-myc-CD8 Stalk-CD8 TM-NruI-CD28-CD3z cyto-speI-sGFP	This study (XSB1176)	Lentiviral vector
19	pHR NdeI-SP-CD22 RFB4 ScFv-BamHI-myc-CD8 Stalk-CD8 TM-NruI-CD28-CD3z cyto-speI-FUS 1-214	This study (XSB1179)	Lentiviral vector
20	pHR NdeI-SP-CD22 RFB4 ScFv-BamHI-myc-CD8 Stalk-CD8 TM-NruI-CD28-CD3z cyto-speI-EWS 28-262	This study (XSB1226)	Lentiviral vector
21	pHR NdeI-SP-CD22 RFB4 ScFv-BamHI-myc-CD8 Stalk-CD8 TM-NruI-CD28-CD3z cyto-speI-TAF15 1-180	This study (XSB1226)	Lentiviral vector
22	pHR NdeI-SP- HER2 ScFv -BamHI-myc-CD8 Stalk-CD28 TM-NruI-CD28-41BB-CD3z cyto-sGFP	This study (XSB1094)	Lentiviral vector
23	pHR NdeI-SP-HER2 ScFv -BamHI-myc-CD8 Stalk-CD28 TM-NruI-CD28-41BB-CD3z cyto-speI-TAF15 1-180	This study (XSB1177)	Lentiviral vector
24	pHR NdeI-SP-HER2 ScFv-BamHI-myc-CD8 Stalk-CD28 TM-NruI-CD28-41BB-CD3z cyto-speI-FUS 1-214	This study (XSB1178)	Lentiviral vector
25	pHR NdeI-SP-HER2 ScFv-BamHI-myc-CD8 Stalk-CD28 TM-NruI-41BB-CD3z cyto-speI-EWS 28-262	This study (XSB1231)	Lentiviral vector
26	pHR NdeI-SP-CD19 ScFv-BamHI-myc-CD8 Stalk-CD8 TM-NruI-CD28-41BB-CD3z cyto-speI-EWS 28-262 Y to S-sGFP	This study (XSB1376)	Lentiviral vector
27	pHR NdeI-SP-CD19 ScFv-BamHI-myc-CD8 Stalk-CD8 TM-NruI-CD28-41BB-CD3z cyto-10xIDR 30Y speI-sGFP	This study (XSB1380)	Lentiviral vector

IDR sequence

1	**FUS**	MASND YTQQATQSYGAYPTQPGQGY SQQSSQPYGQQSYSGYSQST<br>DTSGYGQSSYSSYGQSQNTG YGTQSTPQGYGSTGGYGSSQ<br>SSQSSYGQQSSYPGYGQQPAPSSTSGSYGSSSQSSSYGQP<br>QSGSYSQQPSYGGQQQSYGQ QQSYNPPQGYGQQNQYNSSS<br>GGGGGGGGGGNYGQDQSSMS SGGGSGGGYGNQDQSGGGGS<br>GGYGQQDRG
2	**EWS**	GYAQTTQA YGQQSYGTYGQPTDVSYTQA QTTATYGQTAYATSYGQPPT<br>GYTTPTAPQAYSQPVQGYGT GAYDTTTATVTTTQASYAAQ<br>SAYGTQPAYPAYGQQPAATA PTRPQDGNKPTETSQPQSST<br>GGYNQPSLGYGQSNYSYPQV PGSYPMQPVTAPPSYPPTSY<br>SSTQPTSYDQSSYSQQNTYG QPSSYGQQSSYGQQSSYGQQ<br>PPTSYPPQTGSYSQAPSQYSQQSSSYG
3	**TAF15**	MSDSGSYG QSGGEQQSYSTYGNPGSQGY<br>GQASQSYSGYGQTTDSSYGQNYSGYSSYGQSYSQSYGGYE<br>NQKQSSYSQQPYNNQGQQQN MESSGSQGGRAPSYDQPDYG<br>QQDSYDQQSGYDQHQGSYDE QSNYDQQHDSYSQNQQSYHS<br>QRENYSHHTQDDRRDVSRYG EDNRGYGGSQGG
4	**NUP98**	FNKSFGTPFG GGTGGFGTTSTFGQNTGFGT TSGGAFGTSAFGSSNNTGGL<br>FGNSQTKPGGLFGTSSFSQP ATSTSTGFGFGTSTGTANTL<br>FGTASTGTSLFSSQNNAFAQ<br>NKPTGFGNFGTSTSSGGLFG TTNTTSNPFGSTSGSLFGPS<br>SFTAAPTGTTIKFNPPTGTD TMVKAGVSTNISTKHQCITA<br>MKEYESKSLEELRLEDYQAN RKGPQNQVGAGTTTGLFGSS<br>PATSSATGLFSSSTTNSGFA YGQNKTAFGTSTTGFGTNPG<br>GLFGQQNQQTTSLFSKPFGQ ATTTQNTGFSFGNTSTIGQP<br>STNTMGLFGVTQASQPGGLF GTATNTSTGT
5	**TDP43**	SNRQLERSGR FGGNPGGFGNQGGFGNSRGG GAGLGNNQGSNMGGGMNFGA<br>FSINPAMMAAAQAALQSSWG MMGMLASQQNQSGPSGNNQN<br>QGNMQREPNQAFGSGNNSYS GSNSGAAIGWGSASNAGSGS<br>GFNGGFGSSMDSKSSGWGM
6	**SynIDR**	SKGPGRGDSPY SGRGDSPYSGRGDSPYSGRG DSPYSGRGDSPYSGRGDSPY<br>SGRGDSPYSGRGDSPYSGRG<br>DSPYSGRGDSPYSGY
7	**EWS28-262 (Y to S)**	GSAQTTQASGQQSSGTSGQP TDVSSTQAQTTATSGQTASA<br>TSSGQPPTGSTTPTAPQASS QPVQGSGTGASDTTTATVTT<br>TQASSAAQSASGTQPASPAS GQQPAATAPTRPQDGNKPTE<br>TSQPQSSTGGSNQPSLGSGQ SNSSSPQVPGSSPMQPVTAP<br>PSSPPTSSSSTQPTSSDQSS SSQQNTSGQPSSSGQQSSSG<br>QQSSSGQQPPTSSPPQTGSS SQAPSQSSQQSSSSG
8	**SynIDR 30Y**	YSKGYPGRYGDSYPYSYGRG YDSPYYSGYRGDYSPYYSGR<br>YGDSYPYSYGRGYDSPYYSG YRGDYSPYYSGRYGDSYPYS<br>YGRGYDSPYYSGYRGDYSPY YSGRYGDSYPYSYGY

Antibodies

1	PE anti-human CD19 Antibody(4G7)	BioLegend	#392506
2	PE anti-human CD22 Antibody(S-HCL-1)	BioLegend	#363503
3	PE anti-human CD340 (erbB2/HER-2) Antibody(24D2)	BioLegend	#324405
4	APC anti-human CD19 Antibody(4G7)	BioLegend	#392504
5	APC anti-human CD22 Antibody(S-HCL-1)	BioLegend	#363506
6	Alexa Fluor® 647 anti-human CD340 (erbB2/HER-2) Antibody(24D2)	BioLegend	#324412
7	APC anti-human CD69 Antibody (FN50)	BioLegend	#310910
8	Brilliant Violet 421™ anti-human CD69 Antibody (FN50)	BioLegend	#310930
9	APC anti-human CD366 (TIM3) Monoclonal Antibody (F38-2E2)	Invitrogen	#17-3109-42
10	PE anti-human CD279 (PD-1) Monoclonal Antibody (MIH4)	Invitrogen	#12-9969-42
11	PE-Cyanine7 anti-human CD223 (LAG-3) Monoclonal Antibody (3DS223H)	Invitrogen	#25-2239-42
12	Pacific Blue™ Mouse Anti-Human CD3 (UCHT1)	BioLegend	#300417
13	PerCP/Cyanine5.5 anti-human CD45RA Antibody (HI100)	BioLegend	#304122
14	Pacific Blue™ anti-human CD4 Antibody (OKT4)	BioLegend	#317424
15	BD Horizon™ BUV496 Mouse Anti-Human CD4(SK3)	BDbiosciences	#612936
16	Brilliant Violet 785™ anti-human CD8 Antibody (SK1)	BioLegend	#344739
17	PE/Cy7 anti-human CD8a (RPA-T8)	BioLegend	#301012
18	APC/Fire™ 750 anti-human CD62L Antibody (DREG-56)	BioLegend	#304845
19	Brilliant Violet 750™ anti-human CD45RA Antibody (HI100)	BioLegend	#304166
20	BD Phosflow™ Alexa Fluor® 647 Mouse anti-CD247 (pY142) (K25-407.69)	BDbiosciences	#558489
21	Alexa Fluor® 647 anti-LAT Phospho (Tyr171) Antibody (A20005D)	BioLegend	#946603
22	Myc-Tag Mouse mAb (Alexa Fluor® 647 Conjugate) (9B11)	Cell Signaling Technology	#2233
23	APC/Fire™ 750 anti-human/mouse Granzyme B Recombinant Antibody (QA18A28)	BioLegend	#396417
24	PerCP anti-human CD45 Antibody (HI30)	BioLegend	#304026
25	APC anti-human CD45 Antibody (HI30)	BioLegend	#304012
26	Monoclonal Anti-FMC63 Antibody, Mouse IgG1 (Y45)	ACROBiosystems	#FM3-Y45
27	Phospho-Zap-70(Tyr319)/Syk (Tyr352) Antibody	Cell Signaling Technology	#2701
28	Phospho-LAT (Tyr191) antibody	Cell Signaling Technology	#3584s

29	Phospho-Lck (Tyr505) antibody	Cell Signaling Technology	#2751
30	Phospho-PLCγ1(Tyr783) antibody	Cell Signaling Technology	#2821
31	Phospho-BTK/ITK (Tyr551, Tyr511) antibody	eBioscience	#14-9015-82
32	Phospho-SLP-76 (Ser376) (E3G9U) antibody	Cell Signaling Technology	#76384
33	Phospho-p44/42 MAPK(Erk1/2) (Thr202/Tyr204) antibody	Cell Signaling Technology	#9101
34	Human Phospho-NFATC1 (S172) Antibody	R&D System	#MAB5640
35	Myc-Tag (9B11)	Cell Signaling Technology	#2276
36	Purified anti-GAPDH Antibody (FF26A/F9)	BioLegend	#649202

Others

1	EasySep™ Human T Cell Isolation Kit	Stem Cell	#17951
2	Human T-Activator CD3/CD28 Dynabeads	ThermoFisher	#11161D
3	Recomninant Interleukin-2	PEPROTECH	# 200-02
4	Human Recombinant Biotinylated Her2/ERBB2 protein	Sino Biological	#10004-H08H-B
5	Human Recombinant Biotinylated CD22 protein	Sino Biological	#11958-H41H-B
6	Recombinant Human ICAM-1/CD54 Protein (His Tag)	Sino Biological	# 10346-H08H
7	Streptavidin, Alexa Fluor™ 647 conjugate	Thermo Fisher Scientific	#S21374
8	Luciferase Assay System	Promega	# E1500
9	Cell lysis reagent	Promega	# 1531
10	Red Blood cell lysis buffer	BioLegend	#420301
11	D-Luciferin, Sodium Salt	Gold Biotechnology	#LUCNA-100
12	Fixation/Permeabilization Solution	BD Bioscience	# 554714
13	TNFα ELISA kit	BioLegend	# 430204
14	BD Quantibrite PE Phycoerythrin Fluorescence Quantitation Kit	BD Bioscience	#340495
15	LEGENDplex™ Human CD8/NK Panel (13-plex)	BioLegend	#741186
16	Chromium Next GEM Single Cell 3' Kit v3.1	10xGenomics	#PN-1000269
17	CellMask™ Plasma Membrane Stains	Invitrogen™	#10046

18	Indo-1, AM, cell permeant	Thermo Fisher Scientific	#I1223
19	Celltrace™ Far Red Cell Proliferation Kit	Thermo Fisher Scientific	# C34564
